# Classification of Isolates from the *Pseudomonas fluorescens* Complex into Phylogenomic Groups Based in Group-Specific Markers

**DOI:** 10.3389/fmicb.2017.00413

**Published:** 2017-03-15

**Authors:** Daniel Garrido-Sanz, Eva Arrebola, Francisco Martínez-Granero, Sonia García-Méndez, Candela Muriel, Esther Blanco-Romero, Marta Martín, Rafael Rivilla, Miguel Redondo-Nieto

**Affiliations:** Departamento de Biología, Facultad de Ciencias, Universidad Autónoma de MadridMadrid, Spain

**Keywords:** *Pseudomonas fluroescens* complex, PCR, phylogroups, classification

## Abstract

The *Pseudomonas fluorescens* complex of species includes plant-associated bacteria with potential biotechnological applications in agriculture and environmental protection. Many of these bacteria can promote plant growth by different means, including modification of plant hormonal balance and biocontrol. The *P. fluorescens* group is currently divided into eight major subgroups in which these properties and many other ecophysiological traits are phylogenetically distributed. Therefore, a rapid phylogroup assignment for a particular isolate could be useful to simplify the screening of putative inoculants. By using comparative genomics on 71 *P. fluorescens* genomes, we have identified nine markers which allow classification of any isolate into these eight subgroups, by a presence/absence PCR test. Nine primer pairs were developed for the amplification of these markers. The specificity and sensitivity of these primer pairs were assessed on 28 field isolates, environmental samples from soil and rhizosphere and tested by *in silico* PCR on 421 genomes. Phylogenomic analysis validated the results: the PCR-based system for classification of *P. fluorescens* isolates has a 98.34% of accuracy and it could be used as a rapid and simple assay to evaluate the potential of any *P. fluorescens* complex strain.

## Introduction

The *Pseudomonas fluorescens* complex of species is one of the most diverse groups within the *Pseudomonas* genus, comprising more than fifty validly named species and many unclassified isolates. Members of this group have been isolated from diverse habitats, including water (Mirand and Zemelman, 2002), soil (Andersen et al., 2000), plant tissues (Brown et al., 2012), fungi (Rainey et al., 1993), animals (Vela et al., 2006), and humans (Scales et al., 2015). Many *P. fluorescens* strains that have been isolated from plant-related environments are described as plant growth-promoting rhizobacteria (PGPR) due to their ability to influence plant hormonal balance (Kang et al., 2006) and improve plant fitness by minimizing the effects of phytopathogens (Raaijmakers et al., 2009), for which they are of great biotechnological interest. Pseudomonads are also known for the utilization of diverse organic compounds as energy and carbon sources (Lessie and Phibbs, 1984), making them also suited for bioremediation of polluted environments (Wasi et al., 2013). Despite their beneficial role as PGPR, certain species within the *P. fluorescens* complex are pathogens, including *Pseudomonas corrugata* and *Pseudomonas mediterranea*, the causal agents of pith necrosis in tomato (Catara, 2007; Trantas et al., 2015) and *Pseudomonas tolaasii*, which causes brown blotch disease on cultivated mushrooms (Rainey et al., 1993).

An analysis of the *P. fluorescens* complex carried out 25 years ago identified several biotypes by using different taxonomic criteria (Stanier et al., 1966; Palleroni, 1991). More recently multilocus sequence analysis (MLSA) has divided the *P. fluorescens* complex into a varying number of groups depending on the number and identity of the genomes included in the study (Mulet et al., 2010; Gomila et al., 2015; Garrido-Sanz et al., 2016). These MLSA-based analysis have shown good concordance with phylogenomics and comparative genomics (Garrido-Sanz et al., 2016), in which eight phylogroups have been identified: *P*. *mandelii, P. jessenii, P. koreensis, P. corrugata, P. fluorescens, P. gessardii, P. chlororaphis*, and *Pseudomonas protegens*.

We have previously shown that many ecophysiological traits that are important for biocontrol, plant growth-promotion and bioremediation are distributed phylogenetically among the main groups within the *P. fluorescens* complex (Garrido-Sanz et al., 2016). For instance, strains within the *P. corrugata, P. chlororaphis*, and *P. protegens* groups produce an array of secondary metabolites with antifungal properties, such as 2,4-diacetylphloroglucinol, 2-hexyl, 5-propyl resorcinol, phenazines, and other siderophore-based antibiotics such as pyrrolnitrin and pyoluteorin (Nowak-Thompson et al., 1999; Raaijmakers and Weller, 2001; Mavrodi et al., 2006; Ramette et al., 2011; Loper et al., 2012; Calderón et al., 2013). It has also been reported that insecticidal activity of *P. fluorescens* complex strains also follows a phylogenetic distribution, being present in *P. protegens* and *P. chlororaphis* phylogroups (Flury et al., 2016). On the other hand, strains from *P. chlororaphis* and *P. koreensis* also carry the biosynthetic gene cluster for indole-3-acetic acid metabolism (Loper et al., 2012; Garrido-Sanz et al., 2016), which enhances plant root system and thus increase the nutrient uptake by the plant (Spaepen et al., 2007). Therefore, routinely phylogroup identification of isolates from the *P. fluorescens* complex could be a preliminary step toward obtaining strains with potential biotechnological applications.

Scarpellini et al. (2004) first developed a 16S rRNA PCR-based assay to identify *P. fluorescens* isolates and their corresponding biotype. Several genotipes on DAPG-producing fluorescent pseudomonads were also identified by PCR using the BOX1AR primer (BOX-PCR; Weller et al., 2007). Presently, the increase in the number of full sequenced genomes allows the use of phylogenetic and comparative genomic methods, which have provided a more robust insight regarding the delineation of groups within the *P. fluorescens* complex of species (Mulet et al., 2010; Gomila et al., 2015; Garrido-Sanz et al., 2016). Different PCR-based systems have been developed for the identification of pathogenic *P. fluorescens* complex species, such as *P. tolaasii* (Lee et al., 2002), *P. corrugata*, and *P. mediterranea* (Catara et al., 2000; Licciarcdello et al., 2011). Although a *rpoD*-based PCR procedure also exists for *Pseudomonas* species identification (Mulet et al., 2009), given that currently the *P. fluorescens* complex is composed by more of 50 named species and that most of the phylogroups contain several species (Garrido-Sanz et al., 2016) it is necessary to develop a system that allows the identification of these mayor *P. fluorescens* complex phylogroups, as group adscription can provide insights into the potential biotechnological uses of a particular isolate.

The present study aims to develop a rapid PCR-based system assay for routinely classification of *P. fluorescens* isolates into the eight phylogroups in which it is currently divided (Garrido-Sanz et al., 2016). For this purpose, we have used comparative genomics to identify specific markers of these groups and develop sets of primers for their amplification. We have tested this system on classified and unclassified *Pseudomonas* strains along with environmental and rhizosphere samples. Finally, we performed *in silico* PCR (*is*PCR) and phylogenomic analysis to theoretically test and validate the system in all the sequenced genomes available.

## Materials and methods

### Datasets, markers identification, and primer design

Seventy-one genomes and proteomes previously reported of belonging to the *P. fluorescens* complex (Garrido-Sanz et al., 2016) were downloaded from the NCBI FTP server (ftp.ncbi.nih.gov) in February 2015 (Supplementary Table 1). Orthologous groups were identified by comparing all-against-all using BLASTP (Camacho et al., 2009) and processed with OrthoMCL v4 pipeline (Li et al., 2003), using default settings, 50% alignment coverage cut-off and 1e-5 *e*-value. The results were stored in a relational database for further analysis and filtered with own designed SQL queries and Python scripts to obtain the CDSs from the protein entries that appeared in all the genomes of each group but not in the remaining genomes. These CDSs were additionally filtered by 500 pb minimum length and blasted against all the genomes. The sequences with no hits across genomes outside the groups and high homology within genomes of the group were selected. The selected markers sequences were then retrieved from all the group's genomes and aligned using Clustal Omega (Sievers and Higgins, 2014). Conserved regions were used to design primers. Degenerated bases were introduced to guarantee annealing in all the target genomes. Melting temperature of the primers, absence of dimerization and hairpin formation and lack of secondary priming sites were assessed with OligoAnalyzer 3.1 (https://eu.idtdna.com/calc/analyzer).

### *In silico* PCR (*is*PCR) and phylogenomic analysis

Sequences of the nine selected markers plus 100 nts from each end were blasted against nt and wgs NCBI databases on March 2016. Blast hits were processed and reviewed to test primer specificity based on mismatches, specially in 3′ and predicted amplicon length.

**Figure 1:**
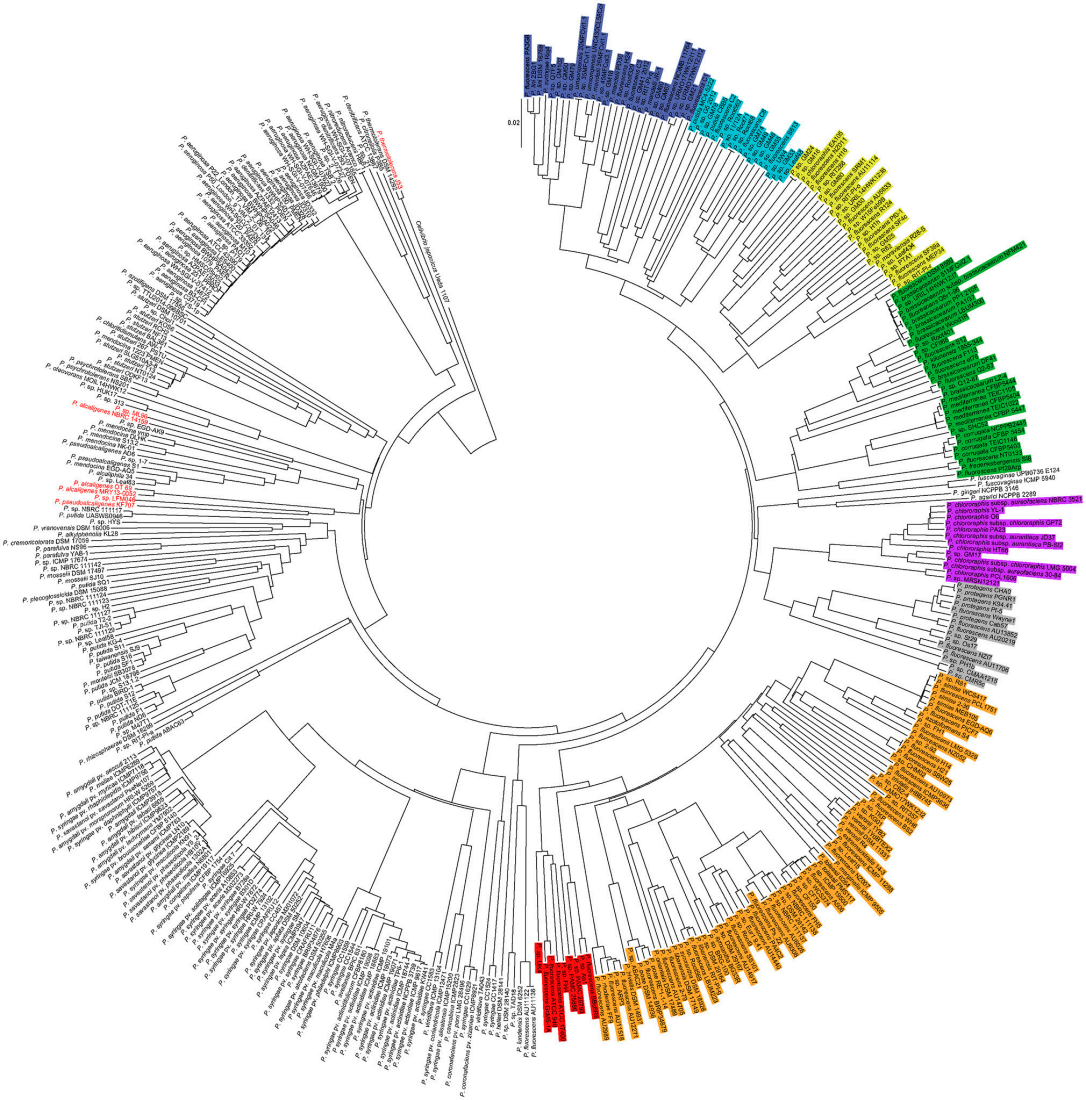
**Phylogenomic tree of 421 Pseudomonas genomes**. In different colors are highlighted the *P. fluorescens* complex phylogroups (according to Garrido-Sanz et al., 2016). Blue, *P*. *mandelii*; Light blue, *P*. *jessenii;* Yellow, *P*. *koreensis*; Green, *P*. *corrugate*; Orange, *P*. *fluorescens*; Red, *P*. *gessardii*; Purple, *P*. *chlororaphis*; Gray, *P*. *protegens*. Genomes in red indicate false positives. *Cellvibrio japonicus* Ueda 107 was used as outgroup.

The 225 genomes selected from *is*PCR as being part of any of the 8 *P. fluorescens* phylogroups (Figure 1) along with 196 randomly sampled genomes from the remaining *Pseudomonas* genomes (retrieved from NCBI ftp server on March, 2016; Supplementary Table 1) were compared all-against-all using the Genome-to-genome distance calculator (GGDC) 2.1 web service at http://ggdc.dsmz.de (Meier-Kolthoff et al., 2013). Resulting sets of intergenomic distances (Supplementary File 1) were used to construct a distance matrix with own designed Python scripts. Phylogenomic trees were built using Neighbor-joining with MEGA v7 software (Kumar et al., 2016).

### *Pseudomonas* strains isolation

Rhizospheric and endophytic pseudomonads were isolated from pepper (*C. annuum*), tomato (*S. lycopersicum*), lettuce (*L. sativa*), and pumpkin (*Cucurbita* sp.). Non-lignified roots were collected, cleaned out from soil remains, introduced in sterile tubes with 25 ml of saline solution (NaCl 0.85%) and vortexed for 5 min to detach soil residues. For rhizospheric pseudomonads isolation, 100 μl of serial dilutions from the saline solution resuspension were plated in SA medium (Scher and Baker, 1982) supplemented with MgSO_4_, ampicillin (100 μg/ml) and cycloheximide (100 μg/ml) and incubated at 28°C for 24 h. For endophytic pseudomonads isolation, roots vortexed in saline solution were further surface-sterilized with 10% sodium hypochlorite for 5 min, then 10% ethanol for 5 min and finally rinsed five times with sterile distilled water for 3 min each. To check complete disinfection, 100 μl from the last wash water was plated on LB medium. Disinfected roots were macerated aseptically in saline solution and then filtered. One hundred microliters of the extract were spreaded on SA plates supplemented with MgSO_4_, ampicillin (100 μg/ml) and cycloheximide (100 μg/ml) and incubated at 28°C for 72 h. Fluorescent colonies under UV light from both rhizospheric and endophytic protocols were re-plated and grown at 28 and 37°C for 24 h. Those unable to grow at 37°C were selected. Additionally, rhizosphere samples 1 and 2 where collected from onion (*Allium cepa*) and lettuce respectively, and were obtained as above but instead of isolates, the whole plates where swept, followed by total DNA extraction (see below).

Strains 7.3 and 3.2 were isolated by standard enrichment culture techniques, using an inoculum of 1 g of soil collected in a petrol station located in Tres Cantos (Madrid, Spain). A mineral medium (Brazil et al., 1995), containing biphenyl as the sole carbon source was used for enrichment and purification of the culture as described previously (Chang et al., 2013).

### Strains cultures, DNA extraction, and PCR conditions

All the strains used in this work were grown in LB medium (Luria-Bertrani) at 28°C, 1.5% (w/v) purified agar for solid medium. DNA extraction from strains cultures was carried out using the Realpure Genomic DNA Extraction Kit (Durviz, Spain). Metagenomic DNA from 1 g soil was extracted using the FastDNA® SPIN Kit for Soil (MP Biomedicals, USA).

PCRs were carried out in a total volume of 25 μl containing 2.5 μl of 10X PCR buffer MgCl_2_ free, 1 μl MgCl_2_ 50 mM, 0.5 μl dNTP mix 10 mM (2.5 μM each), 1 μl of each primer at 10 μM, 1 μl of *Taq* DNA polymerase 1 U/μl (Biotools) and 1 μl of DNA template 30–50 ng/μl. The cycling conditions consisted in a first denaturation step at 95°C for 5 min followed by 30 cycles of amplification (30 s denaturation at 95°C, 30 s of primer annealing at 64°C for DGPf_0, DGPf_1, DGPf_2, DGPf_3, DGPf_4, and DGPf_7 or 67°C for DGPf_5, DGPf_6 and DGPf_8 primer sets, and an elongation step at 72°C for 1.5 min) followed by a final elongation step at 72°C for 7 min. For the metagenomic DNA of environmental samples, the number of cycles was increased to 40. PCR products were electrophoretically separated in 1.5% (w/v) agarose gels and post-dyed with GelRed.

### 16S rDNA cloning

16S rDNA from the strains described in Supplementary File 2, was amplified using the universal primers 27F (5′-AGAGTTTGATCMTGGCTCAG-3′) and 1492R (5′-CTACGRRTACCTTGTTACGAC-3′). PCR conditions as described above, using an annealing temperature of 56°C, 25 cycles, and using 1 μl of Ultratools DNA polymerase (Biotools) 1 U/μl. PCR results were cloned into pGEM®-T Easy Vector System I (Promega) and transformed into *E. coli* DH5α. Plasmid DNA was extracted using the kit Wizard® Plus SV Minipreps DNA Purification System (Promega). Inserts were sequenced using the universal primers T7 and SP6. All the 16S rDNA sequences obtained in this study were submitted to the GenBank and are available under the accession numbers specified in Supplementary File 2.

## Results and discussion

### Nine markers are sufficient to identify the eight *P. fluorescens* phylogroups

In order to identify markers that allow the classification of a given strain into one of the eight *P. fluorescens* phylogroups, all-against-all genome comparisons were performed on 71 *P. fluorescens* complex genomes (Supplementary Table 1) representing all these groups. The subsequent filtering for highly conserved coding sequences belonging to the core genome of each of the eight phylogroups revealed that the combination of nine markers in a pattern of presence and/or absence are enough for their discrimination (Table 1). Interestingly, the putative function of most of these genes, three of which are transcriptional regulators, does not seem to confer any distinctive group phenotype, but are rather involved in general functions. It is important to notice that adscription of any strain to a given group requires, at least, two positive reactions.

**Table 1:** *****P. fluorescens*** complex specific markers and their distribution within the phylogroups**.

**Name**	**Gene name (accs. no.)^b^**	***P. fluorescens*** **complex group**^a^							
		***P. corrugata***	***P. koreensis***	***P. jessenii***	***P. mandelii***	***P. gessardii***	***P. fluorescens***	***P. protegens***	***P. chlororaphis***
DGPf_0	Hypothetical protein (PSF113_RS556935)	+	+	±	+	−	−	±	−
DGPf_1	Type I secretion target (PSF113_RS30625)	+	−	−	−	−	−	−	−
DGPf_2	FAD dependent oxidoreductase (PFL01_RS10805)	−	+	+	−	−	−	+	+
DGPf_3	Glutamine synthetase (PputUW4_01890)	−	−	+	+	−	−	−	−
DGPf_4	KWG repeat-containing protein (PFL_RS20920)	−	−	−	−	−	−	+	−
DGPf_5	3-phosphoshikimate 1-carboxyvinyltransferase (PFLA506_RS14455)	−	−	−	−	+	+	−	−
DGPf_6	LysR family transcriptional regulator (PFL_RS18605)	−	−	−	−	+	−	±	−
DGPf_7	LysR family transcriptional regulator (PFLU_RS11255)	−	−	−	−	−	+	−	−
DGPf_8	LuxR family transcriptional regulator (PCL1606_12410)	−	−	−	−	−	−	−	+

*The presence of the marker in all the strains of a group is indicated with a plus (+) while absence is indicated with a minus (−). Groups in which the marker can be present or absent depending on the strain is indicated with plus/minus (±)*.

a *Groups according to Garrido-Sanz et al. (2016)*.

b *Accession number of the most representative gene sequence within each group*.

Strains from the *P. corrugata* phylogroup can be identified with the markers DGPf_0 and DGPf_1 (Table 1). While DGPf_0 codifies for a hypothetical protein (PSF113_RS56935) that is also present in *P. koreensis, P. mandelii*, and some strains from *P. jessenii* and *P. protegens* phylogroups, DGPf_1 codifies for a type I secretion target (PSF113_RS30625) that was only found in strains belonging to the *P. corrugata* group. Primer pairs for both markers were designed to amplify the regions between nucleotides 139 to 750 for DGPf_0 and from 632 to 1,316 for DGPf_1, resulting in amplicons of 621 and 685 bp respectively (Table 2).

**Table 2:** **Primers designed in this work**.

**Marker name**	**Primer name**	**Position^a^**	**Sequence (5′-3′)**	**Tm (°C)**	**Length (bp)**
DGPf_0	DGPf_0F	139..750	CATCGCAATCGCAC**R**ATGAT**Y**	64	612
	DGPf_0R		GAAAGTCTTGACCAGCA**RV**AG		
DGPf_1	DGPf_1F	632..1316	TGCAGG**R**AGACGG**S**AA**R**G	64	685
	DGPf_1R		CC**R**AGGAAGCCCAGGGA**N**		
DGPf_2	DGPf_2F	88..1088	GT**R**GTSTTCATCGG**B**GG**H**GG	64	1001
	DGPf_2R		TGGCA**R**TACCAGACGTT**R**TCCG		
DGPf_3	DGPf_3R	671..1351	CCATGGCCGACCACCACGTCATCATCAA**R**C	64	681
	DGPf_3R		GCAGTTCCCAGTCGGT**K**AT**B**CG**Y**CGGTCG		
DGPf_4	DGPf_4F	32..1103	CGCTGATCCTCTCGTTGTCTGC	64	1072
	DGPf_4R		ACGCCCTTGTCCACATCG		
DGPf_5	DGPf_5F	3..1117	CGGCGTGGGTGTCGATC**RR**	67	1115
	DGPf_5R		GAGTTCGCAGAAAACCGTGACCG		
DGPf_6	DGPf_6F	28..707	GC**S**TTGCG**H**TA**Y**TTCCACGAGG	67	680
	DGPf_6R		GCCAGGCT**Y**TTCTGCAC**Y**TCC		
DGPf_7	DGPf_7F	11..755	C**Y**GA**R**ATCGAGGGGCT**K**TGGA	64	745
	DGPf_7R		GCTGAA**R**TCTGG**V**AGCAGGGC		
DGPf_8	DGPf_8F	127..787	CCCACCGACAGCCAGCAACG	67	661
	DGPf_8R		CGGTCTTGTCGCTGATGCCG		

*Degenerated bases are indicated in bold*.

a *Target marker position for the primers according to genes described in Table 1*.

For the *P. koreensis* phylogroup, aside from DGPf_0, the marker DGPf_2 was identified (Table 1). This second marker encodes for a FAD dependent oxydoreductase (PFL01_RS10805) and primer pair was designed to amplify the region from nucleotides 88 to 1,088, producing a fragment of 1,001 bp (Table 2). DGPf_2 was also present in all the strains from *P. jessenii, P. protegens* and *P. chlororaphis* phylogroups.

For the *P. jessenii* phylogroup, the markers DGPf_2 and DGPf_3 were designed to identify this group strains (Table 1). DGPf_3 encodes for a glutamine synthetase (PputUW4_01890) which primer pair was designed in the 671 to 1,351 region, resulting in a 681 bp amplicon (Table 2). Additionally, marker DGPf_0 was detected in most of the strains from this group (i.e., *P*. sp. UW4, *P*. sp. GM33, *P*. sp. GM55, *P*. sp. GM49, *P*. sp. GM74, *P*. sp. GM78 and *P*. sp. GM48, and *P*. sp. G5). In any case, either if a certain strain results positive for the amplification of markers DGPf_2 and DGPf_3, or for both of them and DGPf_0, there is no other phylogroup that can be misidentified with these markers combinations (Table 1).

The *P. mandellii* phylogroup can be identified with the combination of DGPf_0 and DGPf_3 markers as described above, and the lack of the DGPf_2 one (Table 1).

For the *P. protegens* phylogroup, aside from DGPf_2, the marker DGPf_4 was identified (Table 1). The DGPf_4 marker encodes for a KWG repeat-containing protein (PFL_RS20920) and primer set was developed to amplify the region between nucleotides 32 and 1,103, which amplifies a fragment of 1,072 bp (Table 2). Additionally, markers DGPf_0 and DGPf_6 were also present on some of the strains from this group (Table 1).

For the *P. gessardii* phylogroup, markers DGPf_5 and DGPf_6 were identified (Table 1). The marker DGPF_5 encodes for a 3-phosphoshikimate 1-carboxyvinyltransferase (PFLA506_RS14455) whose primer pair was developed to amplify the region between nucleotides 3 and 1,117 pb, resulting in an amplicon of 1,115 bp (Table 2). This marker is also present in all the strains from *P. fluorescens* phylogroup. On the other hand, DGPf_6 encodes for a LysR family transcriptional regulator (PFL_RS18605) and aside from all the *P. gessardii* strains it is also present in some *P. protegens* phylogroup strains. Primer set was designed to amplify a 680 bp fragment from nucleotide 28 to nucleotide 707 pb of this gene (Table 2).

For the *P. fluorescens* phylogroup, aside from DGPf_5, the specific DGPf_7 marker was identified (Table 1). This marker encodes for another LysR family transcriptional regulator (PFLU_RS11255) and primer set was designed in the 11 to 755 pb region, resulting in a 745 bp fragment amplification (Table 2).

Finally, for the *P. chlororaphis* phylogroup, the combination of DGPf_2 and the specific DGPf_8 markers are able to identify strains belonging to this group. DGPf_8 encodes for a LuxR family transcriptional regulator (PCL1606_12410) and primer set was designed to amplify the region between 127 to 787 pb (Table 2).

As expected, given the genetic heterogeneity of the *P. fluorescens* complex genomes, we did not find any highly conserved specific marker for the *P. koreensis, P. jessenii, P. mandelii*, and *P. gessardii* phylogroups. However, the combination of two different markers allows their correct affiliation. On the other hand, *P. corrugata, P. fluorescens, P. protegens*, and *P. chlororaphis* groups do have markers that are exclusive to them. Therefore, the combination of the presence and/or absence of these nine markers allows the classification of a given strain into one of these groups with at least two different markers present in each of them (Table 1).

Primer sequences were aligned against all the genomes from their group. When mismatches where detected, degenerated bases were introduced to ensure optimal primer hybridization. Primer sequences and their marker target regions for PCR amplification are listed in Table 2. After *in silico* PCR (see below), primer sequences were checked again and degenerated bases were introduced to maximize theoretical hybridization in the 147 additional *P. fluorescens* complex genomes identified.

### PCR test of model *P. fluorescens* strains

Primer sets and annealing temperatures were empirically tested in gradient temperature PCRs in nine model *P. fluorescens* complex strains (*P. fluorescens* F113, *P. fluorescens* Q2-87, *P. fluorescens* Q8r1-96 (renamed *P. brassicacearum* Q8r1-96), *P. brassicacearum* NFM421, *P. chlororaphis* PCL 1319, *P. chlororaphis* PCL1606, *P. protegens* Pf-5, *P. fluorescens* Pf0-1, and *P. fluorescens* SBW25) belonging to five different phylogroups (*P. corrugata, P. chlororaphis, P. protegens, P. koreensis*, and *P. fluorescens* respectively). The optimal annealing temperatures are specified in Table 2. Additionally, three other *Pseudomonas* model strains, outside the *P. fluorescens* complex were tested as negative controls (*P. putida* KT2440, *P. syringae* pv. *tomato* DC3000, and *P. aeruginosa* PAO1) along with *Escherichia coli* DH5α. All the PCRs for the *P. fluorescens* complex strains resulted in positive amplification for the markers of the groups they belong to (Table 3), with amplicon sizes congruent with the theoretically expected. For the negative controls, no amplification was observed with the exception of the DGPf_2 primer set in *P. putida* KT2440 (Table 3) that does not allow the classification of the strain as belonging to any *P. fluorescens* phylogroup.

**Table 3:** *****P. fluorescens*** complex strains and other ***Pseudomonas*** tested for their ***P. fluorescens*** complex phylogroup affiliation**.

**Strain tested**	**Markers**									
	**DGPf_0**	**DGPf_1**	**DGPf_2**	**DGPf_3**	**DGPf_4**	**DGPf_5**	**DGPf_6**	**DGPf_7**	**DGPf_8**	***P. fluorescens* phylogroup identified**
*P. fluorescens* F113	+	+	−	−	−	−	−	−	−	*P. corrugata*
*P. fluorescens* Q2-87	+	+	−	−	−	−	−	−	−	*P. corrugata*
*P. fluorescens* Q8r1-96	+	+	−	−	−	−	−	−	−	*P. corrugata*
*P. brassicacearum* NFM421	+	+	−	−	−	−	−	−	−	*P. corrugata*
*P. chlororaphis* PCL 1319	−	−	+	−	−	−	−	−	+	*P. chlororaphis*
*P. chlororaphis* PCL1606	−	−	+	−	−	−	−	−	+	*P. chlororaphis*
*P. protegens* Pf-5	−	−	+	−	+	−	+	−	−	*P. protegens*
*P. fluorescens* Pf0-1	+	−	+	−	−	−	−	−	−	*P. koreensis*
*P. fluorescens* SBW25	−	−	−	−	−	+	−	+	−	*P. fluorescens*
*P. putida* KT2440	−	−	+	−	−	−	−	−	−	−
*P. syringae* pv. *tomato* DC3000	−	−	−	−	−	−	−	−	−	−
*P. aeruginosa* PAO1	−	−	−	−	−	−	−	−	−	−
*E. coli* DH5α	−	−	−	−	−	−	−	−	−	−

*PCR positive result is indicated with a plus (+) while a negative result is indicated with a minus (−)*.

### Blind PCR test of *P. fluorescens* isolates and environmental samples

Seventeen putative *P. fluorescens* isolates from rhizosphere and endosphere of pepper (*Capsicum annuum*), tomato (*Solanum lycopersicum*), lettuce (*Lactuca sativa*), and pumpkin (*Cucurbita* sp.) along with two other soil isolates (Supplementary Table 2) were tested for their phylogroup affiliation. PCR results unequivocally assigned each isolate to a single phylogroup. Six (31.6%) of the isolates belonged to the *P. koreensis* phylogroup, another six (31.6%) for the *P. jessenii* phylogroup, five (26.3%) isolates were placed in the *P. fluorescens* phylogroup, and two (10.5%) were classified in *P. corrugata* phylogroup (Table 4). No isolate from *P. chlororaphis, P. protegens, P. mandelii*, or *P. gessardii* groups were found. 16S rDNA sequence of 10 of these strains was obtained (EMC3, EMC5, RMT7, 3.2, RMT1, RMT2, EMC7, RMT4, RMP9, and 7.3) and showed high sequence identity with strains from the phylogroups they were assigned to by the PCR system (Supplementary File 2). Additionally, whole-genome sequence of four of these isolates (EMC3, 3.2, RMP9, and 7.3) and subsequent phylogenomic analysis validated their correct group affiliation (data not shown).

**Table 4:** *****Pseudomonas*** isolates and environmental samples affiliation to the different ***P. fluorescens*** complex phylogroups**.

**Isolate/sample tested**	**Markers**									
	**DGPf_0**	**DGPf_1**	**DGPf_2**	**DGPf_3**	**DGPf_4**	**DGPf_5**	**DGPf_6**	**DGPf_7**	**DGPf_8**	***P. fluorescens* phylogroup identified**
EMC3^a^^,^^b^	+	−	+	−	−	−	−	−	−	*P. koreensis*
EMC5^a^	+	−	+	−	−	−	−	−	−	*P. koreensis*
RMT7^a^	+	−	+	−	−	−	−	−	−	*P. koreensis*
RMC4	+	−	+	−	−	−	−	−	−	*P. koreensis*
RMC9	+	−	+	−	−	−	−	−	−	*P. koreensis*
3.2^ab^	+	−	+	−	−	−	−	−	−	*P. koreensis*
EMC11	+	−	+	+	−	−	−	−	−	*P. jessenii*
RMT1^a^	+	−	+	+	−	−	−	−	−	*P. jessenii*
RMT2^a^	+	−	+	+	−	−	−	−	−	*P. jessenii*
RMC8	+	−	+	+	−	−	−	−	−	*P. jessenii*
HFL1	+	−	+	+	−	−	−	−	−	*P. jessenii*
HFL4	+	−	+	+	−	−	−	−	−	*P. jessenii*
EMC7^a^	−	−	−	−	−	+	−	+	−	*P. fluorescens*
EMT2	−	−	−	−	−	+	−	+	−	*P. fluorescens*
EMT8	−	−	−	−	−	+	−	+	−	*P. fluorescens*
RMT4^a^	−	−	−	−	−	+	−	+	−	*P. fluorescens*
RMT12	−	−	−	−	−	+	−	+	−	*P. fluorescens*
RMP9^a^^,^^b^	+	+	−	−	−	−	−	−	−	*P. corrugata*
7.3^a^^,^^b^	+	+	−	−	−	−	−	−	−	*P. corrugata*
Soil sample	+	−	−	+	−	+	−	+	−	*P. mandelii* and *P. fluorescens*
Rhizosphere sample 1	+	+	+	+	−	+	−	+	−	*P. corrugata, P. koreensis, P. jessenii, P. mandelii*, and *P. fluorescens*
Rhizosphere sample 2	+	−	+	+	−	+	−	+	+	*P. koreensis, P. jessenii, P. mandelii, P. fluorescens*, and *P. chlororaphis*

a *Group adscription confirmed by 16S rDNA sequence*.

b *Group adscription confirmed by whole-genome sequence*.

An environmental soil sample was also tested. Here, four primer sets gave positive results; DGPf_0, DGPf_3, DGPf_5, and DGPf_7 (Table 4), suggesting the presence of DNA from *P. mandelii* and *P. fluorescens* phylogroups. Two additional rhizosphere samples (1 and 2) where tested, resulting in amplification for primer sets DGPf_0, DGPf_1, DGPf_2, DGPf_3, DGPf_5, and DGPf_7 for sample 1 and DGPf_0, DGPf_2, DPGf_3, DGPf_5, DGPf_7, and DGPf_8 for sample 2 (Table 4). These primer combination suggest the presence of DNA from *P. corrugata, P. koreensis, P. jessenii, P. mandelii*, and *P. fluorescens* in sample 1 and *P. koreensis, P. jessenii, P. mandelii, P. fluorescens*, and *P. chlororaphis* in sample 2, although other sources cannot be dismissed. On the other hand, the absence of certain markers in these samples strongly suggests lack of certain phylogroups. For instance, negative amplification of markers DGPf_1, DGPf_2, DGPf_4, DGPf_6, and DGPf_8 in the soil sample makes the presence of *P. corrugata, P. koreensis, P. jessenii, P. gessardii, P. protegens*, and *P. chlororaphis* phylogroups unlikely. However, in rhizosphere samples, where most of strains from the *P. fluorescens* complex phylogroups can be found, the number of primer sets resulting in negative amplification are reduced. This finding makes the system not only suited for testing isolates, but also for testing environmental samples in order to assess the diversity of *P. fluorescens* complex phylogroups that might be present.

### *In silico* PCR and phylogenomics of *Pseudomonas* genomes

To test the extent of the PCR-based system to reliably characterize new isolates, *is*PCR was assessed by blasting the nine markers against the whole NCBI nt and wgs databases on March 2016, which included more than 65,000 prokariotic genomes, and checking for the marker combination presence, primer mismatches (specially in 3′) and theoretical amplicon length in all genomes. *is*PCR identified 225 genomes which gave a positive identification pattern (see in detail in Supplementary File 3). This dataset also included the 71 genomes used for generating the markers set. Thirty-two of these genomes (14.22%) belonged to the *P. corrugata* phylogroup, 28 (12.44%) to the *P. koreensis* phylogroup, 23 (10.22%) to the *P. jessenii* phylogroup, 28 (12.44%) to the *P. mandelii* phylogroup, 9 (4%) to the *P. gessardii* phylogroup, 77 (34.22%) to the *P. fluorescens* phylogroup, 15 (6.67%) to the *P. protegens* phylogroup and 13 (5.78%) to the *P. chlororaphis* phylogroup.

These 225 genomes were further analyzed by phylogenomics in order to corroborate their classification. Another 196 genomes randomly sampled from the remaining 2,310 *Pseudomonas* genomes available at the NCBI ftp server (ftp.ncbi.nih.gov, March 2016) were also included as negative control. As shown in Figure 1, from the 225 genomes identified as belonging to the *P. fluorescens* complex by *is*PCR, 218 (96.9%) showed total correlation between the PCR data and phylogenomic assignment, indicating that they were correctly placed within each group by the PCR method. The remaining seven genomes (3.1%), were false positives and they all belonged to the *P. aeruginosa* lineage (Garrido-Sanz et al., 2016). Two of these genomes (*P*. sp. ML96, and *P. pseudoalcaligenes* KF707) were wrongly identified by *is*PCR as belonging to the *P. koreensis* phylogroup, while the other four (*P. alcaligenes* NBCRC 14159, *P*. sp. LFMO46, *P. thermotolerans* J53, *P. alcaligenes* OT 69 and *P. alcaligenes* MRY13-0052) were wrongly identified as belonging to the *P. jessenii* phylogroup (Supplementary File 3). On the other hand, from the 196 randomly sampled genomes, none of them were shown to be false negatives. In consequence, the *is*PCR has shown that the system has a 98.34% of accuracy, as from the 421 genomes tested, just seven were misidentified.

Interestingly, the phylogenomic analysis has also shown the presence of two more phylogroups within the same branch that leads to the *P. fluorescens* complex (Figure 1). The most distal one includes *P. lundensis* DSM 6252, the novel species *P. helleri* (von Neubeck et al., 2016) and other isolates. *P. lundensis* has been previously reported to belong to the *P. fragi* phylogroup within the *P. fluorescens* complex (Mulet et al., 2010; Gomila et al., 2015; Garrido-Sanz et al., 2016). However, this phylogroup has only been identified based on MLSA, as no sequenced genome was available at the time of these analysis. For the same reason, this group was not included for the markers identification. The other group, identified as *P. asplenii* group, is shown to be within the branch that leads into the *P. chlororaphis* and *P. protegens* phylogroups. However, none of the previous studies of the *Pseudomonas* genus place it inside of the *P. fluorescens* complex (Mulet et al., 2010; Gomila et al., 2015; Garrido-Sanz et al., 2016). These discrepancies with previous studies could be due to the larger number of genomes included here. The three species within the *P. asplenii* phylogroup (*P. asplenii, P. fuscovaginae*, and *P. agarici*) are known pathogens of fern, rice and mushrooms respectively (Ark and Tompkins, 1946; Batoko et al., 1994; Gill and Cole, 2000), therefore their adscription to the *P. fluorescens* complex should be further analyzed. In any case, none of the genomes from these groups resulted in positive amplification for any of the markers. More worrying is the finding that several strains have been wrongly classified at the species level. This is the case of *P. syringae* Riq4, placed now within the *P. mandelii* phylogroup, *P. putida* strains MC4-5222 and CBB5, which really belong to the *P. jessenii* phylogroup, *P. chlororaphis* EA105, in the *P. koreensis* phylogroup and *P. frederiksbergensis* SI8 in the *P. corrugata* phylogroup (Figure 1). All these strains clearly do not belong to the species they have been assigned to, highlighting that more efforts should be done to avoid inaccurate and misleading species naming and the need for databases curing.

## Conclusions

The results presented here provide an accurate, easy to perform and cheap method for the initial profiling of strains belonging to the *P. fluorescens* complex of species and isolated from plant material or soil. The method allows the placement of each isolate unequivocally in one of the eight phylogroups that have been previously described by MLSA and digital DNA-DNA hybridization. It also allows the assessment of the presence/absence of specific phylogroups in complex samples (metagenomic DNA from soil or plant samples). Considering the phylogenetic distribution of biotechnology relevant traits, the method could be included as one of the first steps in protocols that require the screening of a large number of pseudomonads isolates in the search for inoculants for agriculture or environmental technologies.

## Author contributions

Performed experiments and Bioinformatic analysis: DGS, MR. Strains Isolation and Characterization: DGS, EA, FM, SG, CM, EBR, RR. Manuscript Drafting: DGS, MM, RR, MR. Experimental Design and Supervision: MM, RR, MR.

### Conflict of interest statement

The authors declare that the research was conducted in the absence of any commercial or financial relationships that could be construed as a potential conflict of interest.

## Supplementary material

Supplementary Table 1 **List of genomes used in this study**.

Supplementary Table 2 *****Pseudomonas*** isolates used for blind test**.

Supplementary File 1 **GGDC intergenomic distances of 421 ***Pseudomonas*** genomes**.

Supplementary File 2 **Closest-related strain and phylogroup adscription according to 16S sequence identity**.

Supplementary File 3 *****In silico*** PCR results**.
