# Supplementary material for: Classification of Isolates from the Pseudomonas fluorescens Complex into Phylogenomic Groups Based in Group-Specific Markers
Source: Front Microbiol. 2017 Mar 15;8:413. doi: 10.3389/fmicb.2017.00413 (PMC5350142; doi:10.3389/fmicb.2017.00413)
Supplement: Supplementary Table 1 — List of genomes used in this study. [file Table1.PDF]

Supplementary table 1. General features of the genomes used in this study.

Genome name	Assembly	Size (MB)	GC%	Ascc. No.	Scaffolds	Gene	Protein	*Pseudomonas* phylogroup[1]
P. fluorescens PA3G8	GCA_000800625.1	6.3916	58.80	JBOO01	9	5689	5551	*P. fluorescens / P. mandelii*
P. lini ZBG1	GCA_001238395.1	6.54508	58.70	LFQO01	327	5865	5721	*P. fluorescens / P. mandelii*
P. lini DSM 16768	GCA_001042905.1	6.4955	58.80	JYLB01	26	5804	5675	*P. fluorescens / P. mandelii*
P. syringae Riq4	GCA_001238485.1	6.36114	58.80	LFQK01	131	5709	5559	*P. fluorescens / P. mandelii*
P. sp. QTF5	GCA_000512695.1	6.03936	58.70	AZRW01	110	5481	5319	*P. fluorescens / P. mandelii*
P. sp. GM102	GCA_000282555.1	6.65735	59.00	AKJB01	159	5945	5773	*P. fluorescens / P. mandelii*
P. sp. GM50	GCA_000282375.1	6.69214	59.00	AKJK01	155	5984	5840	*P. fluorescens / P. mandelii*
P. sp. GM79	GCA_000282495.1	6.70701	58.80	AKJE01	126	5987	5834	*P. fluorescens / P. mandelii*
P. umsongensis 20MFCvi1.1	GCA_000377725.1	6.45866	59.40	ARIW01	27	5829	5725	*P. fluorescens / P. mandelii*
P. sp. 35MFCvi1.1	GCA_000378525.1	6.45755	59.40	ARKL01	26	5839	5733	*P. fluorescens / P. mandelii*
P. umsongensis UNC430CL58Col	GCA_000620285.1	6.45617	59.30	JHVT01	28	5823	5704	*P. fluorescens / P. mandelii*
P. mandelii 36MFCvi1.1	GCA_000381285.1	6.55399	59.20	ARLP01	29	5886	5768	*P. fluorescens / P. mandelii*
P. sp. 45MFCol3.1	GCA_000382025.1	6.56265	59.40	ARMZ01	21	5877	5769	*P. fluorescens / P. mandelii*
P. sp. GM18	GCA_000282195.1	6.29762	59.50	AKJT01	140	5659	5516	*P. fluorescens / P. mandelii*
P. mandelii PD30	GCA_000690555.2	6.68584	59.00	AZQQ01	109	6058	5942	*P. fluorescens / P. mandelii*
P. fluorescens H24	GCA_000968015.1	6.8991	58.80	LACH01	168	6197	6059	*P. fluorescens / P. mandelii*
P. sp. Root329	GCA_001424925.1	6.73355	59.10	LMCV01	51	6121	5987	*P. fluorescens / P. mandelii*
P. fluorescens C3	GCA_000967955.1	6.70557	59.00	LACD01	76	6069	5942	*P. fluorescens / P. mandelii*
P. sp. GM41(2012)	GCA_000282315.2	6.68503	59.00	AKJN02	13	5985	5864	*P. fluorescens / P. mandelii*
P. sp. RIT-PI-q	GCA_001297125.1	7.44602	59.10	LHPC01	82	6659	6503	*P. fluorescens / P. mandelii*
P. mandelii JR-1	GCA_000257545.3	7.18856	58.97	NZ_CP005960.1	-	6551	6377	*P. fluorescens / P. mandelii*
P. sp. GM60	GCA_000282415.1	6.42424	59.60	AKJI01	181	5850	5704	*P. fluorescens / P. mandelii*

P. sp. GM67	GCA_000282435.1	6.50211	59.60	AKJH01	183	5909	5797	*P. fluorescens / P. mandelii*
P. fluorescens NCIMB 11764	GCA_000293885.3	6.99815	59.00	NZ_CP010945.1	-	6354	6205	*P. fluorescens / P. mandelii*
P. sp. URMO17WK12:I11	GCA_001511755.1	6.37444	59.20	NZ_LN854573.1	-	5728	5582	*P. fluorescens / P. mandelii*
P. sp. URMO17WK12:I12	GCA_000514395.1	6.56843	59.10	AZVV01	32	5981	5849	*P. fluorescens / P. mandelii*
P. sp. GM21	GCA_000282215.1	6.60927	58.50	AKJS01	210	5951	5769	*P. fluorescens / P. mandelii*
P. fluorescens C1	GCA_000967945.1	6.29633	58.90	LACE01	47	5735	5616	*P. fluorescens / P. mandelii*
P. putida MC4-5222	GCA_000729805.1	6.8581	59.70	JOJW01	1152	6239	6026	*P. fluorescens / P. jessenii*
P. sp. G5(2012)	GCA_000408945.1	7.20456	59.30	APIO01	223	6548	6404	*P. fluorescens / P. jessenii*
P. sp. GM78	GCA_000282475.1	7.28756	60.20	AKJF01	235	6609	6406	*P. fluorescens / P. jessenii*
P. putida CBB5	GCA_001006135.1	6.81706	60.00	JTEN01	146	6082	5918	*P. fluorescens / P. jessenii*
P. fluorescens C2	GCA_000802965.1	6.42011	59.00	JSAK01	64	5808	5665	*P. fluorescens / P. jessenii*
P. sp. Root562	GCA_001427125.1	6.21602	59.00	LMGK01	26	5573	5444	*P. fluorescens / P. jessenii*
P. sp. 11/12A	GCA_000800055.1	6.77845	59.60	JUGV01	2	6164	5994	*P. fluorescens / P. jessenii*
P. sp. Root71	GCA_001429045.1	6.24993	60.00	LMHY01	30	5629	5481	*P. fluorescens / P. jessenii*
P. sp. Root68	GCA_001427765.1	6.25093	60.00	LMHI01	25	5628	5482	*P. fluorescens / P. jessenii*
P. fluorescens C8	GCA_000967965.1	6.28701	60.00	LACC01	90	5641	5518	*P. fluorescens / P. jessenii*
P. sp. GM74	GCA_000282455.1	6.10428	60.10	AKJG01	180	5504	5360	*P. fluorescens / P. jessenii*
P. sp. GM49	GCA_000282355.1	6.58989	59.60	AKJL01	345	6082	5759	*P. fluorescens / P. jessenii*
P. sp. GM48	GCA_000282335.1	6.44379	59.30	AKJM01	200	5789	5628	*P. fluorescens / P. jessenii*
P. sp. GM55	GCA_000282395.1	6.48933	59.70	AKJJ01	163	5856	5700	*P. fluorescens / P. jessenii*
P. fluorescens S613	GCA_001317545.1	6.7339	59.60	LJXB01	94	6056	5862	*P. fluorescens / P. jessenii*
P. sp. UW4	GCA_000316175.1	6.18339	60.10	NC_019670.1	-	5511	5376	*P. fluorescens / P. jessenii*
P. sp. GM33	GCA_000282295.1	6.72722	60.10	AKJO01	205	5982	5844	*P. fluorescens / P. jessenii*
P. sp. Leaf48	GCA_001421885.1	5.68945	59.40	LMLH01	33	5083	4947	*P. fluorescens / P. jessenii*

P. sp. GM24	GCA_000282235.1	6.51706	59.10	AKJR01	399	5794	5671	*P. fluorescens / P. koreensis*
P. sp. GM16	GCA_000282155.1	6.55052	59.20	AKJV01	128	5810	5618	*P. fluorescens / P. koreensis*
P. chlororaphis EA105	GCA_000783395.1	6.59558	59.20	JSFK01	74	5788	5672	*P. fluorescens / P. koreensis*
P. fluorescens NZ011	GCA_000276585.1	6.81443	58.50	AJXJ01	973	5955	5623	*P. fluorescens / P. koreensis*
P. fluorescens H16	GCA_000802985.1	6.80779	59.10	JSAL01	78	6098	5971	*P. fluorescens / P. koreensis*
P. sp. RIT288	GCA_000631985.1	6.27329	60.00	JFYN01	44	5581	5452	*P. fluorescens / P. koreensis*
P. sp. GM80	GCA_000282515.1	6.78616	59.20	AKJD01	282	6144	5983	*P. fluorescens / P. koreensis*
P. fluorescens SMR1	GCA_000827755.2	6.35298	59.4	CDMF01	332	-	5829	*P. fluorescens / P. koreensis*
P. fluorescens AU11114	GCA_001020875.1	6.20469	58.80	LCZE01	64	5600	5444	*P. fluorescens / P. koreensis*
P. sp. RIT-PI-o	GCA_001297215.1	6.10366	60.00	LHPA01	54	5374	5251	*P. fluorescens / P. koreensis*
P. sp. URIL14HWK12:I6	GCA_000514195.1	6.33775	60.00	AZVL01	61	5653	5522	*P. fluorescens / P. koreensis*
P. sp. GM30	GCA_000282275.2	6.15244	60.30	AKJP02	32	5526	5422	*P. fluorescens / P. koreensis*
P. fluorescens AU5633	GCA_001020865.1	6.0834	60.30	LCZD01	56	5471	5340	*P. fluorescens / P. koreensis*
P. sp. W15Feb9B	GCA_000828175.1	6.03159	60.40	JTKF01	102	5331	5257	*P. fluorescens / P. koreensis*
P. fluorescens R124	GCA_000292795.1	6.30049	60.25	NZ_CM001561.1 /ALYL01	2	5601	5461	*P. fluorescens / P. koreensis*
P. sp. H1h	GCA_000633255.1	6.36815	60.30	AYMJ01	78	5677	5549	*P. fluorescens / P. koreensis*
P. fluorescens Pf0-1	GCA_000012445.1	6.43841	60.50	NC_007492.2	-	5773	5631	*P. fluorescens / P. koreensis*
P. fluorescens SF4c	GCA_000817905.1	6.50701	60.50	JTGH01	47	5812	5695	*P. fluorescens / P. koreensis*
P. sp. GM25	GCA_000282255.1	6.35061	60.90	AKJQ01	91	5692	5565	*P. fluorescens / P. koreensis*
P. sp. R62	GCA_000257605.1	6.58357	58.90	AHZM01	192	5662	4884	*P. fluorescens / P. koreensis*
P. moraviensis R28-S	GCA_000512275.1	6.30832	59.76	NZ_CM002330.1 /AYMZ01	2	5669	5523	*P. fluorescens / P. koreensis*
P. sp. Leaf434	GCA_001425545.1	5.86803	60.10	LMQZ01	14	5207	5093	*P. fluorescens / P. koreensis*
P. sp. PTA1	GCA_000745605.1	6.09429	59.70	JQJQ01	42	5459	5307	*P. fluorescens / P. koreensis*
P. fluorescens SF39a	GCA_000817895.1	5.88423	60.00	JTGG01	50	5191	5085	*P. fluorescens / P. koreensis*

P. fluorescens MEP34	GCA_000834545.1	5.91424	60.20	JXQY01	58	5152	5047	*P. fluorescens / P. koreensis*
P. sp. RIT-PI-r	GCA_001297015.1	6.51898	59.30	LIGE01	49	5832	5675	*P. fluorescens / P. koreensis*
P. fluorescens DSM 8569	GCA_000876175.1	6.63984	61.00	JXOE01	135	5895	5766	*P. fluorescens / P. corrugata*
P. brassicacearum 51MFCVI2.1	GCA_000510785.1	6.56887	61.00	AZOC01	49	5834	5725	*P. fluorescens / P. corrugata*
P. sp. URIL14HWK12:I7	GCA_000514275.1	6.88333	60.70	AZVP01	78	6080	5958	*P. fluorescens / P. corrugata*
P. brassicacearum subsp. *brassicacearum* NFM421	GCA_000194805.1	6.84325	60.80	NC_015379.1	-	6047	5916	*P. fluorescens / P. corrugata*
P. fluorescens Q8r1-96	GCA_000263695.2	6.60261	61.00	NZ_CM001512.1 /AHPO01	1	5808	5697	*P. fluorescens / P. corrugata*
P. brassicacearum PP1_210F	GCA_000785375.1	6.77162	60.80	AYJR01	5	5982	5710	*P. fluorescens / P. corrugata*
P. brassicacearum PA1G7	GCA_000800585.1	6.78944	60.80	JBON01	8	6025	5912	*P. fluorescens / P. corrugata*
P. brassicacearum LBUM300	GCA_001449085.1	6.97676	60.80	NZ_CP012680.1	-	6126	5998	*P. fluorescens / P. corrugata*
P. fluorescens Wood1R	GCA_000285615.1	6.68132	60.80	CAFF01	1437	5967	5710	*P. fluorescens / P. corrugata*
P. sp. Root401	GCA_001425105.1	7.02955	60.50	LMDO01	66	6120	5982	*P. fluorescens / P. corrugata*
P. sp. CFII68	GCA_000416195.1	5.9196	60.80	ATLN01	128	5231	5089	*P. fluorescens / P. corrugata*
P. fluorescens S12	GCA_000498415.1	6.36933	60.70	AVFN01	332	5626	5469	*P. fluorescens / P. corrugata*
P. kilonensis 1855-344	GCA_000968575.1	6.84372	60.70	JZXC01	73	5995	5853	*P. fluorescens / P. corrugata*
P. fluorescens F113	GCA_000237065.1	6.84583	60.80	NC_016830.1	-	6034	5898	*P. fluorescens / P. corrugata*
P. fluorescens et76	GCA_001468775.2	6.68165	60.70	LNAB02	68	5790	5646	*P. fluorescens / P. corrugata*
P. brassicacearum DF41	GCA_000585995.1	6.6524	60.50	NZ_CP007410.1	-	5736	5554	*P. fluorescens / P. corrugata*
P. fluorescens Q2-87	GCA_000281895.1	6.36817	60.60	NZ_CM001558.1 /AGBM01	1	5655	5496	*P. fluorescens / P. corrugata*
P. sp. Q12-87	GCA_001269755.1	6.30362	60.70	LHVI01	45	-	-	*P. fluorescens / P. corrugata*
P. brassicacearum LZ-4	GCA_001017815.1	6.21787	60.10	JNCR01	111	5534	5386	*P. fluorescens / P. corrugata*
P. mediterranea CFBP5444	GCA_001412045.1	6.29886	61.20	LIHG01	89	5463	5345	*P. fluorescens / P. corrugata*
P. mediterranea TEIC1105	GCA_001412185.1	6.29933	61.20	LIGN01	91	5459	5341	*P. fluorescens / P. corrugata*
P. mediterranea CFBP5404	GCA_001411985.1	6.28154	61.30	LIGZ01	150	5451	5324	*P. fluorescens / P. corrugata*

P. mediterranea TEIC1022	GCA_001412005.1	6.23124	61.30	LJWU01	101	5425	5249	*P. fluorescens / P. corrugata*
P. mediterranea CFBP 5447	GCA_000774145.1	6.31969	61.20	AUPB01	32	5526	5414	*P. fluorescens / P. corrugata*
P. sp. SHC52	GCA_000801235.1	6.31259	61.00	CBLV01	384	5600	5285	*P. fluorescens / P. corrugata*
P. corrugata NCPPB2445	GCA_001411965.1	6.08394	60.60	LIGR01	104	5277	5139	*P. fluorescens / P. corrugata*
P. corrugata CFBP 5454	GCA_000522485.1	6.19389	60.50	ATKI01	157	5422	5292	*P. fluorescens / P. corrugata*
P. corrugata TEIC1148	GCA_001412065.1	6.26658	60.30	LIHH01	431	5544	5290	*P. fluorescens / P. corrugata*
P. corrugata CFBP5403	GCA_001412195.1	6.16658	60.40	LIGO01	442	5503	5062	*P. fluorescens / P. corrugata*
P. fluorescens NT0133	GCA_000952735.1	6.12486	60.70	JYHW01	110	5477	5288	*P. fluorescens / P. corrugata*
P. frederiksbergensis S18	GCA_000802155.2	6.57	60.5	JQGJ01	65	5.922	5721	*P. fluorescens / P. corrugata*
P. fluorescens Pf29Arp	GCA_000346775.1	6.1318	60.90	ANOR01	69	5519	5272	*P. fluorescens / P. corrugata*
P. fuscovaginae UPB0736	GCA_000251185.1	6.70843	60.80	AIEU01	102	5641	5424	*P. asplenii*
P. fuscovaginae ICMP 5940	GCA_000467065.1	6.37897	61.20	BATG01	459	5888	4039	*P. asplenii*
P. gingeri NCPPB 3146	GCA_000280765.1	7.66664	-	AKBP01	1036	-	-	*P. asplenii*
P. agarici NCPPB 2289	GCA_000280785.1	5.51139	58.90	AKBQ01	119	4864	4398	*P. asplenii*
P. chlororaphis subsp. *aureofaciens* NBRC 3521	GCA_000813225.1	6.97334	62.80	BBQB01	59	6258	6132	*P. fluorescens / P. chlororaphis*
P. chlororaphis YL-1	GCA_000512485.1	6.80098	63.10	AWWJ01	82	6090	5978	*P. fluorescens / P. chlororaphis*
P. chlororaphis O6	GCA_000264555.1	6.98025	62.90	NZ_CM001490.1 /AHOT01	1	6232	6101	*P. fluorescens / P. chlororaphis*
P. chlororaphis subsp. *chlororaphis* GP72	GCA_000237045.2	6.62988	63.10	AHAY01	347	6008	5774	*P. fluorescens / P. chlororaphis*
P. chlororaphis PA23	GCA_000698865.1	7.12217	62.60	NZ_CP008696.1	-	6358	6233	*P. fluorescens / P. chlororaphis*
P. chlororaphis subsp. *aurantiaca* JD37	GCA_000761195.1	6.70206	62.80	NZ_CP009290.1	-	5984	5815	*P. fluorescens / P. chlororaphis*
P. chlororaphis subsp. *aurantiaca* PB-St2	GCA_000506385.1	6.59092	63.20	AYUD01	23	5837	5662	*P. fluorescens / P. chlororaphis*
P. chlororaphis HT66	GCA_000597925.1	7.29862	62.60	ATBG01	50	6491	6340	*P. fluorescens / P. chlororaphis*
P. sp. GM17	GCA_000282175.1	6.78696	62.80	AKJU01	280	6017	5828	*P. fluorescens / P. chlororaphis*
P. chlororaphis subsp. *chlororaphis* LMG 5004	GCA_001269625.1	6.79247	63.00	LHVC01	15	-	-	*P. fluorescens / P. chlororaphis*

P. chlororaphis subsp. *aureofaciens* 30-84	GCA_000281915.1	6.66632	62.90	NZ_CM001559.1 /AHHJ01	1	5891	5764	*P. fluorescens* / *P. chlororaphis*
P. chlororaphis PCL1606	GCA_000963835.1	6.6629	63.98	NZ_CP011110.1	-	5946	5816	*P. fluorescens* / *P. chlororaphis*
P. sp. MRSN12121	GCA_000931465.1	6.98676	63.75	NZ_CP010892.1	-	6380	6249	*P. fluorescens* / *P. chlororaphis*
P. protegens CHA0	GCA_000397205.1	6.86798	63.40	NC_021237.1	-	6169	6070	*P. fluorescens* / *P. protegens*
P. protegens PGNR1	GCA_001269475.1	6.85402	63.40	LHUV01	15	-	-	*P. fluorescens* / *P. protegens*
P. protegens K94.41	GCA_001269485.1	6.95966	63.30	LHUU01	17	-	-	*P. fluorescens* / *P. protegens*
P. protegens Pf-5	GCA_000012265.1	7.07489	63.30	NC_004129.6	-	-	6108	*P. fluorescens* / *P. protegens*
P. fluorescens Wayne1	GCA_000285355.1	6.8158	63.40	CADW01	337	6168	5867	*P. fluorescens* / *P. protegens*
P. protegens Cab57	GCA_000828695.1	6.82789	63.30	NZ_AP014522.1	-	6163	6056	*P. fluorescens* / *P. protegens*
P. fluorescens AU13852	GCA_001020815.1	6.67892	63.30	LCZC01	45	6062	5933	*P. fluorescens* / *P. protegens*
P. fluorescens AU20219	GCA_001021695.1	7.27564	62.80	LDET01	98	6592	6427	*P. fluorescens* / *P. protegens*
P. sp. St29	GCA_001547915.1	6.83312	63.30	NZ_AP014628.1	-	6124	5987	*P. fluorescens* / *P. protegens*
P. sp. Os17	GCA_001547895.1	6.88546	63.50	NZ_AP014627.1	-	6127	5986	*P. fluorescens* / *P. protegens*
P. fluorescens NZI7	GCA_000275905.1	6.81409	63.20	AJXF01	1032	-	-	*P. fluorescens* / *P. protegens*
P. fluorescens AU11706	GCA_001020715.1	7.2569	62.90	LCZB01	137	6511	6338	*P. fluorescens* / *P. protegens*
P. sp. PH1b	GCA_000633395.1	7.43014	62.90	AYMU01	89	6620	6444	*P. fluorescens* / *P. protegens*
P. sp. CMAA1215	GCA_000474765.1	6.65824	63.80	AVOY01	224	6380	6111	*P. fluorescens* / *P. protegens*
P. sp. CMR5c	GCA_001269545.1	6.75558	63.60	LHUY01	44	-	-	*P. fluorescens* / *P. protegens*
P. sp. R81	GCA_000257625.1	6.22577	60.30	AHZN01	8	5689	5469	*P. fluorescens* / *P. fluorescens*
P. simiae WCS417	GCA_000698265.1	6.16907	60.30	NZ_CP007637.1	1	5616	5502	*P. fluorescens* / *P. fluorescens*
P. fluorescens PCL1751	GCA_000934565.1	6.14395	60.40	NZ_CP010896.1	-	5593	5466	*P. fluorescens* / *P. fluorescens*
P. simiae 2-36	GCA_000785125.1	6.39566	60.30	JRMC01	20	5803	5684	*P. fluorescens* / *P. fluorescens*
P. simiae MEB105	GCA_000834615.1	6.10549	60.40	JXQT01	44	5595	5500	*P. fluorescens* / *P. fluorescens*
P. fluorescens EGD-AQ6	GCA_000465595.1	6.08626	60.50	AVQG01	58	5572	5480	*P. fluorescens* / *P. fluorescens*

P. fluorescens PICF7	GCA_000963495.1	6.13673	60.40	NZ_CP005975.1	-	5609	5459	*P. fluorescens / P. fluorescens*
P. azotoformans S4	GCA_001579805.1	6.85962	60.30	NZ_CP014546.1	-	6324	5991	*P. fluorescens / P. fluorescens*
P. sp. FH1	GCA_000510895.2	7.06304	60.10	AOHM01	146	6556	6378	*P. fluorescens / P. fluorescens*
P. fluorescens LMG 5329	GCA_000411675.1	6.87002	60.50	ASGY01	253	6313	6165	*P. fluorescens / P. fluorescens*
P. fluorescens NZ052	GCA_000275925.1	6.82765	60.10	AJXH01	440	6092	5851	*P. fluorescens / P. fluorescens*
P. sp. 2-92(2010)	GCA_000503215.1	6.41961	60.40	AYTD01	19	5860	5730	*P. fluorescens / P. fluorescens*
P. fluorescens H14	GCA_000968025.1	6.67941	60.50	LACG01	77	5974	5859	*P. fluorescens / P. fluorescens*
P. fluorescens H21	GCA_000967935.1	6.68033	60.50	LACF01	76	5963	5860	*P. fluorescens / P. fluorescens*
P. fluorescens SBW25	GCA_000009225.1	6.72254	60.50	NC_012660.1	-	6034	5900	*P. fluorescens / P. fluorescens*
P. sp. CHM02	GCA_000612585.1	6.6578	60.90	JFCA01	132	6064	5936	*P. fluorescens / P. fluorescens*
P. fluorescens AU10973	GCA_000801835.1	6.13111	60.80	JRXV01	23	5528	5418	*P. fluorescens / P. fluorescens*
P. fluorescens ICMP3636	GCA_001466915.1	6.59103	60.90	LKEI01	101	5948	5820	*P. fluorescens / P. fluorescens*
P. trivialis IHBB745	GCA_001186335.1	6.4528	59.90	NZ_CP011507.1	-	5879	5731	*P. fluorescens / P. fluorescens*
P. sp. CBZ-4	GCA_000346755.1	6.49733	61.20	ANNV01	186	5782	5665	*P. fluorescens / P. fluorescens*
P. sp. LAMO17WK12:I2	GCA_000514375.1	6.92894	61.00	AZVU01	34	6287	6159	*P. fluorescens / P. fluorescens*
P. sp. RIT357	GCA_000632245.1	6.17847	59.80	JFYX01	49	5559	5445	*P. fluorescens / P. fluorescens*
P. fluorescens WH6	GCA_000166515.1	6.27087	60.60	NZ_CM001025.1 /AEAZ01	1	5724	5367	*P. fluorescens / P. fluorescens*
P. fluorescens BS2	GCA_000308175.1	6.12326	60.60	AMZG01	122	5554	5415	*P. fluorescens / P. fluorescens*
P. sp. TKP	GCA_000508205.1	7.01267	60.50	NC_023064.1	-	6253	6113	*P. fluorescens / P. fluorescens*
P. sp. KG01	GCA_001050345.1	6.31335	60.10	LFMW01	47	5760	5592	*P. fluorescens / P. fluorescens*
P. veronii 1YB2	GCA_000982395.1	7.64907	60.30	JGYI01	115	6969	6734	*P. fluorescens / P. fluorescens*
P. veronii 1YdBTEX2	GCA_000350565.1	6.68004	60.70	AOUH01	63	6000	5822	*P. fluorescens / P. fluorescens*
P. veronii DSM 11331	GCA_001439695.1	6.99787	60.70	JYLL01	95	6213	6049	*P. fluorescens / P. fluorescens*
P. veronii R4	GCA_000836475.2	6.64982	61.10	JXWQ02	2	5944	5820	*P. fluorescens / P. fluorescens*

P. extremaustralis 14-3	GCA_000242115.2	6.58624	60.60	AHIP01	135	-	5845	*P. fluorescens / P. fluorescens*
P. fluorescens ICMP 11288	GCA_001466835.1	6.52316	60.20	LKEF01	106	5868	5715	*P. fluorescens / P. fluorescens*
P. sp. Leaf15	GCA_001421425.1	6.55679	60.20	LMKI01	37	5923	5801	*P. fluorescens / P. fluorescens*
P. fluorescens NZ007	GCA_000280805.1	6.54229	59.80	AKBR01	141	5826	5492	*P. fluorescens / P. fluorescens*
P. marginalis ICMP 9505	GCA_001467265.1	5.89355	59.60	LKGY01	144	5287	5098	*P. fluorescens / P. fluorescens*
P. tolaasii 6264	GCA_000316215.1	6.23381	61.00	AKYY01	688	5844	5234	*P. fluorescens / P. fluorescens*
P. tolaasii PMS117	GCA_000276565.1	7.00782	60.20	AJXG01	357	6267	5944	*P. fluorescens / P. fluorescens*
P. sp. ICMP 19500	GCA_001467145.1	6.43781	59.90	LKBK01	144	5879	5706	*P. fluorescens / P. fluorescens*
P. fluorescens A506	GCA_000262325.2	6.01955	59.92	NC_017911.1	-	5437	5304	*P. fluorescens / P. fluorescens*
P. sp. WCS374	GCA_000698295.1	6.08505	60.00	NZ_CP007638.1	1	5479	5344	*P. fluorescens / P. fluorescens*
P. sp. CFT9	GCA_000416255.1	6.20533	59.80	ATLM01	67	5657	5522	*P. fluorescens / P. fluorescens*
P. sp. CF150	GCA_000416175.1	6.08791	59.80	ATLQ01	95	5485	5343	*P. fluorescens / P. fluorescens*
P. fluorescens FH5	GCA_000511155.2	6.17843	60.00	AOJA01	87	5550	5432	*P. fluorescens / P. fluorescens*
P. sp. NBRC 111138	GCA_001320835.1	6.06288	60.00	BCBO01	195	5520	5303	*P. fluorescens / P. fluorescens*
P. sp. DSM 28142	GCA_001439745.1	6.73032	59.50	JYLP01	60	6064	5888	*P. fluorescens / P. fluorescens*
P. sp. NBRC 111137	GCA_001320785.1	6.71386	59.80	BCBN01	125	6133	5957	*P. fluorescens / P. fluorescens*
P. fluorescens AU6026	GCA_000801795.1	6.11281	60.00	JRXU01	34	5466	5328	*P. fluorescens / P. fluorescens*
P. fluorescens AU6308	GCA_000801815.1	6.11645	60.00	JRXZ01	29	5466	5334	*P. fluorescens / P. fluorescens*
P. fluorescens Ps_22	GCA_001542715.1	6.42981	59.50	LCYA01	357	6347	4992	*P. fluorescens / P. fluorescens*
P. fluorescens PA4C2	GCA_000785395.1	6.21086	60.10	AXDA01	5	5441	5322	*P. fluorescens / P. fluorescens*
P. fluorescens AU14440	GCA_000801875.1	6.88089	59.50	JRXX01	109	6181	6003	*P. fluorescens / P. fluorescens*
P. sp. Eur1 9.41	GCA_000744215.1	6.33108	60.00	JQLM01	2	5579	5434	*P. fluorescens / P. fluorescens*
P. sp. Root9	GCA_001429205.1	6.55448	59.70	LMIY01	41	5844	5697	*P. fluorescens / P. fluorescens*
P. fluorescens SS101	GCA_000263675.2	6.17954	60.00	NZ_CM001513.1 /AHPN01	1	5446	5319	*P. fluorescens / P. fluorescens*

P. fluorescens AU14917	GCA_000803005.1	6.31814	60.10	JRXY01	36	5684	5541	*P. fluorescens* / *P. fluorescens*
P. sp. DSM 29167	GCA_001439845.1	6.7331	60.00	JYLO01	42	6020	5886	*P. fluorescens* / *P. fluorescens*
P. synxantha BG33R	GCA_000263715.2	6.29749	59.70	NZ_CM001514.1 /AHPP01	1	5570	5415	*P. fluorescens* / *P. fluorescens*
P. sp. BRG-100	GCA_000737955.1	6.24666	59.60	JPRX01	4	5505	5371	*P. fluorescens* / *P. fluorescens*
P. sp. DSM 29164	GCA_001439735.1	6.01521	59.70	JYLN01	37	5318	5208	*P. fluorescens* / *P. fluorescens*
P. libanensis RIT-PI-g	GCA_001297075.1	6.16447	60.00	LHOY01	54	5534	5403	*P. fluorescens* / *P. fluorescens*
P. fluorescens LBUM223	GCA_000968415.1	6.6942	59.40	NZ_CP011117.1	-	6037	5838	*P. fluorescens* / *P. fluorescens*
P. sp. Root569	GCA_001427465.1	6.22867	59.90	LMGQ01	29	5599	5476	*P. fluorescens* / *P. fluorescens*
P. synxantha DSM 18928	GCA_001439725.1	6.79298	59.70	JYLJ01	64	6145	5990	*P. fluorescens* / *P. fluorescens*
P. libanensis DSM 17149	GCA_001439685.1	6.21423	60.20	JYLH01	43	5568	5452	*P. fluorescens* / *P. fluorescens*
P. fluorescens 2-79	GCA_000876155.1	6.35818	59.80	JXCQ01	143	5648	5530	*P. fluorescens* / *P. fluorescens*
P. fluorescens AU14705	GCA_000801895.1	6.10709	59.90	JRYA01	97	5433	5306	*P. fluorescens* / *P. fluorescens*
P. orientalis DSM 17489	GCA_001439815.1	6.18864	-	JYLM01	39	5484	5291	*P. fluorescens* / *P. fluorescens*
P. poae RE*1-1-14	GCA_000336465.1	5.51224	60.80	NC_020209.1	-	4820	4694	*P. fluorescens* / *P. fluorescens*
P. fluorescens BRIP34879	GCA_000334015.1	5.52529	60.80	AMZW01	110	4831	4689	*P. fluorescens* / *P. fluorescens*
P. poae DSM 14936	GCA_001439785.1	5.88389	60.50	JYLI01	56	5218	5066	*P. fluorescens* / *P. fluorescens*
P. sp. ABAC21	GCA_001467155.1	5.86456	60.80	LKBL01	2861	5377	4149	*P. fluorescens* / *P. fluorescens*
P. trivialis DSM 14937	GCA_001439805.1	5.58791	61.00	JYLK01	46	4969	4817	*P. fluorescens* / *P. fluorescens*
P. fluorescens AU12271	GCA_000801915.1	5.8219	60.40	JRYP01	24	5272	5148	*P. fluorescens* / *P. fluorescens*
P. sp. ARP3	GCA_001029595.1	6.12771	60.30	LEKF01	63	5594	5449	*P. fluorescens* / *P. fluorescens*
P. fluorescens AU11518	GCA_000801855.1	6.31529	60.30	JRXW01	61	5774	5613	*P. fluorescens* / *P. fluorescens*
P. rhodesiae FF9	GCA_000821225.1	6.05006	-	CCYI01	14	-	-	*P. fluorescens* / *P. fluorescens*
P. fluorescens AU2989	GCA_000801775.1	6.20768	60.40	JRXT01	49	5591	5449	*P. fluorescens* / *P. fluorescens*
P. fluorescens BBc6R8	GCA_000297195.2	6.94648	61.00	AKXH02	153	6351	6219	*P. fluorescens* / *P. gessardii*

P. sp. Ag1	GCA_000278565.1	7.25236	60.50	AKVH01	113	6561	6457	*P. fluorescens / P. gessardii*
P. sp. PAMC 26793	GCA_000313235.1	6.77179	60.60	AMXG01	58	6166	6035	*P. fluorescens / P. gessardii*
P. sp. PAMC 25886	GCA_000242655.2	7.02184	61.20	AHHC01	95	6358	6221	*P. fluorescens / P. gessardii*
P. sp. FH4	GCA_000510915.2	6.02409	60.10	AOHN01	63	5449	5326	*P. fluorescens / P. gessardii*
P. fluorescens ATCC 17400	GCA_000708695.2	6.24744	60.50	JENC01	74	5553	5420	*P. fluorescens / P. gessardii*
P. fluorescens ATCC 948	GCA_001414295.1	5.9276	60.50	JSFM01	52	-	-	*P. fluorescens / P. gessardii*
P. fluorescens GcM5-1A	GCA_001444295.1	6.00758	60.50	JJOE01	94	5403	5267	*P. fluorescens / P. gessardii*
P. sp. UK4	GCA_000174915.1	5.75922	60.40	ACOQ01	2971	-	-	*P. fluorescens / P. gessardii*
P. fluorescens AU11136	GCA_001020805.1	4.83766	58.60	LCYY01	61	4406	4268	*P. fluorescens / P. fragi*
P. fluorescens AU11122	GCA_001020785.1	4.8399	58.60	LCYV01	58	4405	4276	*P. fluorescens / P. fragi*
P. lundensis DSM 6252	GCA_001042985.1	4.99250	-	JYKY01	92	4609	4365	*P. fluorescens / P. fragi*
P. sp. TAD18	GCA_001529305.1	4.91759	57.20	LLWI01	82	4333	4220	*P. fluorescens / P. fragi*
P. sp. DSM 28140	GCA_001042975.1	5.00439	57.10	JYLG01	47	4587	4431	*P. fluorescens / P. fragi*
P. helleri DSM 28141	GCA_001043065.1	5.86526	58.30	JYLE01	167	5263	5072	*P. fluorescens / P. fragi*
P. syringae CC1417	GCA_000452825.2	5.64846	59.30	AVEO02	210	5055	4932	*P. syringae*
P. syringae CC1524	GCA_000452745.2	5.82837	59.10	AVEK02	264	5228	5075	*P. syringae*
P. viridiflava TA043	GCA_000452485.1	5.97921	59.20	AVDV01	218	5282	5180	*P. syringae*
P. coronafaciens pv. *zizaniae* ICMP8921	GCA_001401235.1	5.72917	57.80	LJRT01	152	5148	4943	*P. syringae*
P. syringae CC1629	GCA_000452645.2	5.93293	57.70	AVEE02	261	5420	5163	*P. syringae*
P. coronafaciens pv. *porri* LMG 28496	GCA_001275735.1	6.24006	57.40	JUEU01	369	5688	5403	*P. syringae*
P. cannabina ICMP2823	GCA_001400175.1	5.91654	-	LJPX01	733	-	-	*P. syringae*
P. syringae pv. *alisalensis* ICMP15200	GCA_001401375.1	6.1021	58.70	LJPP01	400	5541	5204	*P. syringae*
P. syringae pv. *coriandricola* ICMP12471	GCA_001400185.1	5.84104	58.60	LJPZ01	425	5316	4993	*P. syringae*
P. viridiflava ICMP 13104	GCA_001466975.1	5.54718	58.90	LKEJ01	204	4916	4394	*P. syringae*

P. syringae CC1583	GCA_000452665.2	5.5072	59.00	AVEF02	308	4908	4761	*P. syringae*
P. syringae pv. *actinidiae* KW41	GCA_000245435.1	5.92183	58.70	AGNP01	428	5330	5019	*P. syringae*
P. syringae pv. *actinidiae* ICMP 9617	GCA_000658965.1	6.4977	57.98	NZ_CM002753.1 /AOKP01	2	5299	5074	*P. syringae*
P. syringae pv. *actinidiae* NCPPB 3739	GCA_000233835.2	5.9279	58.80	AFTH01	815	5447	5038	*P. syringae*
P. syringae pv. *actinidiae* ICMP 18744	GCA_000342185.1	6.23561	58.50	ANGD01	442	5572	5344	*P. syringae*
P. syringae pv. *actinidiae* TP6-1	GCA_000344455.1	6.2241	58.50	ANJH01	483	5584	5347	*P. syringae*
P. syringae pv. *actinidiae* ICMP 19071	GCA_000416485.1	6.02137	58.80	AOJS01	391	5479	5145	*P. syringae*
P. syringae pv. *actinidiae* ICMP 19073	GCA_000416505.1	5.97455	58.80	AOJR01	745	5422	5045	*P. syringae*
P. syringae pv. *actinidiae* ICMP 19101	GCA_000416585.1	6.0038	58.40	AOKM01	1851	5821	4737	*P. syringae*
P. syringae pv. *actinidiae* ICMP 18883	GCA_000416785.2	6.30976	58.70	AOKH01	415	5578	5300	*P. syringae*
P. syringae pv. *actinidiae* ICMP 19098	GCA_000416545.1	6.30819	58.70	AOKE01	315	5546	5337	*P. syringae*
P. syringae pv. *actinidifoliorum* CFBP8180	GCA_001497465.1	6.26167	58.70	LJFN01	256	5433	5297	*P. syringae*
P. avellanae BPIC 631	GCA_000444135.1	5.96274	58.50	ATDK01	611	5300	4990	*P. syringae*
P. syringae CC1544	GCA_000452905.2	5.88355	59.10	AVEI02	376	5198	5050	*P. syringae*
P. syringae CC1559	GCA_000452685.2	5.85835	58.90	AVEG02	365	5194	5015	*P. syringae*
P. syringae pv. *philadelphi* ICMP8903	GCA_001400595.1	6.15027	58.50	LJQY01	479	5488	5155	*P. syringae*
P. syringae pv. *maculicola* M4a	GCA_001294305.1	6.43579	58.40	LGLE01	44	5823	5640	*P. syringae*
P. syringae pv. *maculicola* H7608	GCA_001293925.1	6.30459	58.40	LGLG01	461	5800	5517	*P. syringae*
P. syringae pv. *atrofaciens* DSM 50255	GCA_000498595.1	5.77727	59.20	AWUI01	669	5040	4872	*P. syringae*
P. syringae BRIP34876	GCA_000334035.1	6.01523	58.90	AMXK01	99	5096	4955	*P. syringae*
P. syringae pv. *syringae* CRAFRU11	GCA_000972155.1	5.8593	59.10	ATSU01	179	5016	4893	*P. syringae*
P. syringae DSM 10604	GCA_000597765.1	6.07245	59.00	JALK01	84	5213	5050	*P. syringae*
P. syringae pv. *lapsa* ICMP3947	GCA_001400495.1	5.85043	59.20	LJQQ01	118	5052	4923	*P. syringae*
P. syringae pv. *syringae* SM	GCA_000412165.1	6.1241	58.97	NZ_CM001986.1 /APWT01	2	5314	5135	*P. syringae*

P. syringae pv. *aptata* DSM 50252	GCA_000145905.1	6.36468	58.70	AEAN01	2562	6402	6368	*P. syringae*
P. syringae pv. *japonica* M301072	GCA_000145785.1	6.51632	58.50	AEAH01	3304	8834	8796	*P. syringae*
P. syringae CC457	GCA_000452585.2	5.84269	59.10	AVEB02	356	5069	4915	*P. syringae*
P. syringae pv. *syringae* CRAFRU12	GCA_000972195.1	5.93313	59.40	ATSV01	246	5146	4982	*P. syringae*
P. syringae ICMP 13102	GCA_001466945.1	5.95475	59.30	LKEO01	158	5119	4929	*P. syringae*
P. syringae pv. *syringae* HRI-W 7924	GCA_001535945.1	6.23965	59.10	LIHR01	130	5415	5177	*P. syringae*
P. syringae pv. *syringae* PD2774	GCA_001466875.1	6.35739	59.20	LKEL01	180	5537	5293	*P. syringae*
P. syringae pv. *syringae* HRI-W 7872	GCA_001535875.1	5.8998	59.30	LIHS01	105	5043	4861	*P. syringae*
P. syringae pv. *syringae* B301D	GCA_000988485.1	6.09482	59.20	NZ_CP005969.1	-	5262	5067	*P. syringae*
P. syringae pv. *syringae* B728a	GCA_000012245.1	6.0937	59.20	NC_007005.1	-	5220	5089	*P. syringae*
P. syringae pv. *aceris* M302273	GCA_000145925.1	6.25057	58.90	AEAO01	511	5681	5111	*P. syringae*
P. syringae pv. *aceris* A10853	GCA_001270465.1	6.2852	59.20	LGAR01	196	5483	5275	*P. syringae*
P. syringae pv. *solidagae* ICMP16925	GCA_001401055.1	5.98044	59.20	LJRH01	382	5211	4735	*P. syringae*
P. syringae Cit 7	GCA_000145825.1	6.17205	59.10	AEAJ01	1686	7184	7145	*P. syringae*
P. syringae pv. *papulans* CFBP 1754	GCA_000935795.1	6.11238	59.00	JYHI01	182	5527	5244	*P. syringae*
P. congelans ICMP19117	GCA_001400775.1	5.63065	-	LJQB01	94	-	-	*P. syringae*
P. amygdali pv. *mellea* N6801	GCA_001293975.1	6.10766	57.70	LGLB01	124	5472	5114	*P. syringae*
P. savastanoi pv. *phaseolicola* 1302A	GCA_001294025.1	6.15461	57.70	LGKY01	96	5486	5111	*P. syringae*
P. savastanoi pv. *phaseolicola* HB10Y	GCA_001294035.1	5.94447	57.80	LGKX01	44	5275	4934	*P. syringae*
P. savastanoi pv. *phaseolicola* Y5_2	GCA_001294065.1	6.2057	57.40	LGKV01	50	5228	4885	*P. syringae*
P. syringae pv. *maculicola* KN91	GCA_001294185.1	5.9428	57.80	LGLF01	55	5171	4787	*P. syringae*
P. savastanoi pv. *glycinea* ICMP2189	GCA_001400375.1	5.98352	57.90	LJQL01	430	5411	5037	*P. syringae*
P. savastanoi pv. *glycinea* LN10	GCA_001293765.1	6.2135	57.60	LGLM01	128	5424	5056	*P. syringae*
P. amygdali pv. *sesami* ICMP763	GCA_001401265.1	6.02868	58.10	LJRG01	462	5434	5151	*P. syringae*

P. syringae pv. *broussonetiae* CFBP 5140	GCA_001538205.1	6.10687	57.80	LIII01	357	5633	5121	*P. syringae*
P. amygdali pv. *lachrymans* YM7902	GCA_001293845.1	6.46976	57.70	LGLI01	95	5968	5753	*P. syringae*
P. amygdali pv. *hibisci* ICMP9623	GCA_001400395.1	6.11148	58.40	LJQN01	156	5432	5235	*P. syringae*
P. amygdali pv. *tabaci* 6605	GCA_000275945.1	6.15929	58.00	AJXI01	283	5460	5114	*P. syringae*
P. amygdali ICMP3918	GCA_001400115.1	5.93478	58.20	LJPQ01	222	5288	5051	*P. syringae*
P. syringae pv. *daphniphylli* ICMP9757	GCA_001400275.1	5.93336	58.20	LJQF01	528	5345	4985	*P. syringae*
P. amygdali pv. *morsprunorum* HRI-W 5269	GCA_001535785.1	6.18329	58.10	LIHZ01	157	5634	5338	*P. syringae*
P. savastanoi pv. *savastanoi* PseNe107	GCA_000935695.1	6.07177	58.00	JYHF01	247	5498	5169	*P. syringae*
P. syringae pv. *rhaphiolepidis* ICMP9756	GCA_001400995.1	5.48302	58.30	LJRE01	446	5142	5142	*P. syringae*
P. amygdali pv. *myricae* ICMP7118	GCA_001400555.1	5.95376	58.20	LJQV01	403	5391	5020	*P. syringae*
P. meliae ICMP6289	GCA_001400515.1	5.09797	58.40	LJQT01	504	5034	5034	*P. syringae*
P. amygdali pv. *aesculi* 2113	GCA_001537965.1	6.16294	58.30	LIKY01	324	5646	5233	*P. syringae*

P. putida ABAC63	GCA_001467195.1	5.13991	62.30	LKBN01	3451	4688	3423	*P. putida*
P. sp. RIT-PI-a	GCA_001187875.1	4.73286	62.30	LGIR01	35	4202	4096	*P. putida*
P. rhizosphaerae DSM 16299	GCA_000761155.1	4.68864	-	NZ_CP009533.1	-	4165	3893	*P. putida*
P. sp. M47T1	GCA_000263855.1	6.31063	62.50	AJWX01	88	5677	5497	*P. putida*
P. putida ND6	GCA_000264665.1	6.20245	61.72	NC_017986.1	-	5580	5426	*P. putida*
P. putida F1	GCA_000016865.1	5.95996	61.90	NC_009512.1	-	5331	5207	*P. putida*
P. sp. NBRC 111125	GCA_001320295.1	6.34058	61.60	BCBB01	150	5793	5621	*P. putida*
P. putida DOT-T1E	GCA_000281215.1	6.2607	61.40	CP003734.1	-	5803	5721	*P. putida*
P. putida S12	GCA_000495455.2	6.38243	61.44	NZ_CP009974.1	-	5799	5652	*P. putida*
P. putida BIRD-1	GCA_000183645.1	5.73154	61.70	NC_017530.1	-	5163	5021	*P. putida*
P. sp. S13.1.2	GCA_000292285.1	4.95585	62.00	ALPA01	337	4544	4383	*P. putida*
P. putida JCM 18798	GCA_001312185.1	6.57759	61.60	BBDC01	924	-	-	*P. putida*

P. monteilii SB3078	GCA_000510285.1	6.00009	62.50	NC_023075.1	-	5463	5316	*P. putida*
P. putida SF1	GCA_001027965.1	5.80588	62.50	LDPF01	292	5355	5144	*P. putida*
P. putida S16	GCA_000219705.1	5.98479	62.30	NC_015733.1	-	5437	5243	*P. putida*
P. taiwanensis SJ9	GCA_000500605.1	6.25305	61.80	AXUP01	736	6075	5519	*P. putida*
P. putida S11	GCA_000292775.1	5.97075	62.40	ALXA01	196	3675	3599	*P. putida*
P. putida KG-4	GCA_000987155.1	5.58691	63.00	AYRY01	91	5038	4911	*P. putida*
P. sp. Leaf58	GCA_001422615.1	6.28312	61.40	LMLL01	18	5813	5640	*P. putida*
P. sp. NBRC 111129	GCA_001320435.1	5.6881	62.10	BCBF01	233	5294	5107	*P. putida*
P. sp. TJI-51	GCA_000190455.3	5.8051	62.10	AEWE02	208	5375	5056	*P. putida*
P. putida T2-2	GCA_000710785.1	5.52423	62.60	JALX01	389	5119	4886	*P. putida*
P. sp. NBRC 111127	GCA_001320345.1	5.7852	62.50	BCBD01	352	5437	5073	*P. putida*
P. sp. H2	GCA_000763225.1	5.79041	62.60	JRPO01	132	5116	5010	*P. putida*
P. sp. NBRC 111123	GCA_001320205.1	5.67246	62.90	BCAZ01	108	5085	4936	*P. putida*
P. sp. NBRC 111124	GCA_001320245.1	6.08938	62.40	BCBA01	109	5519	5370	*P. putida*
P. plecoglossicida DSM 15088	GCA_000688275.1	5.34757	63.00	JHYX01	58	4876	4667	*P. putida*
P. putida SQ1	GCA_000802565.1	5.32094	61.60	JTCJ01	1623	5050	4270	*P. putida*
P. mosselii SJ10	GCA_000498975.2	6.24786	63.40	NZ_CP009365.1	-	5694	5533	*P. putida*
P. mosselii DSM 17497	GCA_000621225.1	6.26084	64.00	JHYW01	55	5790	5609	*P. putida*
P. sp. NBRC 111142	GCA_001320925.1	6.32546	62.80	BCBS01	329	5827	5521	*P. putida*
P. sp. ICMP 17674	GCA_000467045.1	5.06476	63.40	BATF01	359	-	-	*P. putida*
P. parafulva YAB-1	GCA_001186195.1	5.11984	61.50	LAWW01	23	4658	4535	*P. putida*
P. parafulva NS96	GCA_001477425.1	4.67109	61.90	LDSN01	162	4217	4073	*P. putida*
P. cremoricolorata DSM 17059	GCA_000425745.1	4.65508	63.50	AUEA01	26	4124	4013	*P. putida*
P. alkylphenolia KL28	GCA_000746525.1	5.76462	-	NZ_CP009048.1	-	5279	5115	*P. putida*

P. vranovensis DSM 16006	GCA_000425805.1	5.69781	-	AUED01	43	-	-	*P. putida*
P. sp. HYS	GCA_000259195.1	5.64603	-	AJJP01	231	-	-	*P. putida*
P. putida UASWS0946	GCA_000878325.1	6.0013	63.90	JXOG01	191	5387	5218	*P. putida*
P. sp. NBRC 111117	GCA_001320045.1	5.53247	64.50	BCAT01	61	4987	4875	*P. putida*
P. pseudoalcaligenes KF707	GCA_000262065.3	6.67805	65.40	AJMR01	229	6106	5898	*P. oleovorans*
P. sp. LFM046	GCA_000949385.1	5.96484	64.30	JYKO01	33	5446	5114	*P. oleovorans*
P. alcaligenes MRY13-0052	GCA_000474255.1	6.87694	65.80	BATO01	237	6188	5926	*P. oleovorans*
P. alcaligenes OT 69	GCA_000455385.1	7.02968	66.00	ATCP01	223	6325	6165	*P. oleovorans*
P. sp. Leaf83	GCA_001422075.1	5.53883	62.40	LMMB01	39	5057	4917	*P. oleovorans*
P. alcaliphila 34	GCA_000319815.1	5.42896	62.60	ANGB01	18	4983	4877	*P. oleovorans*
P. mendocina EGD-AQ5	GCA_000465575.1	5.22557	62.70	AVQF01	19	4772	4681	*P. oleovorans*
P. pseudoalcaligenes S1	GCA_000962895.1	6.88999	62.40	JTFL01	131	6498	6037	*P. oleovorans*
P. sp. 1-7	GCA_000742775.1	4.93101	62.00	JPRQ01	73	4483	4094	*P. oleovorans*
P. pseudoalcaligenes AD6	GCA_000626735.1	5.39226	62.50	JFJN01	198	4929	4775	*P. oleovorans*
P. mendocina NK-01	GCA_000204295.1	5.43435	62.50	NC_015410.1	-	5039	4940	*P. oleovorans*
P. mendocina S13.2	GCA_000725105.2	5.42298	62.40	JOVT01	6	5115	4979	*P. oleovorans*
P. mendocina DLHK	GCA_000287395.1	5.07209	64.70	ALKM01	33	4632	4496	*P. oleovorans*
P. mendocina ymp	GCA_000016565.1	5.07281	64.70	NC_009439.1	-	4658	4547	*P. oleovorans*
P. sp. EGD-AK9	GCA_000465935.2	5.10204	65.60	AVOF01	1015	4971	4229	*P. oleovorans*
P. alcaligenes NBRC 14159	GCA_000467105.1	4.82339	64.80	BATI01	122	4440	4301	*P. oleovorans*
P. sp. ML96	GCA_000761545.1	4.79678	64.80	JPMY01	47	4522	4432	*P. oleovorans*
P. sp. 313	GCA_000316965.1	5.20302	65.30	ANBZ01	110	4746	4606	*P. oryzihabitans*
P. sp. HUK17	GCA_001566765.1	2.568	0.00	LSMW01	15	2326	2272	*P. oryzihabitans*
P. oleovorans MOIL14HWK12	GCA_000510765.1	5.00589	66.30	AZOB01	12	4581	4507	*P. oryzihabitans*

P. psychrotolerans NS201	GCA_001477245.1	5.26279	64.90	LDSP01	172	4703	4575	*P. oryzihabitans*
P. psychrotolerans SB5	GCA_001476875.1	5.43522	64.60	LDSY01	171	4818	4658	*P. oryzihabitans*
P. stutzeri ODKF13	GCA_001575085.1	4.59133	64.20	LSVE01	85	4207	4134	*P. stutzeri*
P. stutzeri NT0124	GCA_000952205.1	4.59718	64.20	JXTL01	35	4322	4206	*P. stutzeri*
P. stutzeri T13	GCA_000282955.1	4.64894	63.90	ALJB01	71	4391	4268	*P. stutzeri*
P. stutzeri SLG510A3-8	GCA_001038645.1	4.65015	64.00	NZ_CP011854.1	-	4279	4082	*P. stutzeri*
P. mendocina 1223 PMEN	GCA_001062345.1	4.58565	-	JVYC01	201	-	-	*P. stutzeri*
P. stutzeri 267_PSTU	GCA_001064225.1	4.6387	63.80	JVNQ01	144	4257	4093	*P. stutzeri*
P. chloritidismutans AW-1	GCA_000495915.1	5.05635	-	AOFQ01	77	4870	4767	*P. stutzeri*
P. stutzeri BAL361	GCA_000935215.1	4.87896	62.60	JXXD01	398	4677	4383	*P. stutzeri*
P. stutzeri NF13	GCA_000341615.1	4.67467	63.00	AOBS01	82	4314	4181	*P. stutzeri*
P. stutzeri RCH2	GCA_000327065.1	4.60049	62.47	NC_019936.1	-	4301	4191	*P. stutzeri*
P. stutzeri KOS6	GCA_000307775.2	4.94721	62.70	AMCZ02	5	4497	4365	*P. stutzeri*
P. sp. Chol1	GCA_000306015.1	4.86903	64.00	AMSL01	199	4526	4242	*P. stutzeri*
P. stutzeri DSM 10701	GCA_000279165.1	4.17412	63.20	NC_018177.1	-	3869	3742	*P. stutzeri*
P. sp. TTU2014-096BSC	GCA_001446945.1	4.2247	63.20	LKKL01	47	3916	3785	*P. stutzeri*
P. azotifigens DSM 17556	GCA_000425625.1	5.01742	-	AUDU01	100	-	-	*P. stutzeri*
P. sp. YS-1p	GCA_000757505.1	6.38026	66.50	JPYP01	36	5873	5750	*P. aeruginosa*
P. aeruginosa C3719	GCA_000152525.1	6.2221	66.30	AAKV01	1	5692	5228	*P. aeruginosa*
P. aeruginosa B3-CFI	GCA_000455425.1	6.75191	66.20	CBMS01	314	6292	6010	*P. aeruginosa*
P. aeruginosa 14672	GCA_001414165.1	6.34919	66.50	LKPV01	12	5819	5678	*P. aeruginosa*
P. aeruginosa WH-SGI-V-07412	GCA_001451355.1	6.40916	66.40	LLOZ01	53	5960	5840	*P. aeruginosa*
P. aeruginosa 231_PPRO	GCA_001063005.1	6.47189	66.40	JVPC01	110	5991	5839	*P. aeruginosa*
P. aeruginosa AZPAE15033	GCA_000793725.1	6.08723	66.40	JTNN01	66	5620	5502	*P. aeruginosa*

P. sp. HMSC05H02	GCA_000952805.1	6.40131	66.40	JZWM01	151	5968	5648	*P. aeruginosa*
P. aeruginosa 1079_PAER	GCA_001061875.1	6.50656	66.30	JWDS01	459	6067	5773	*P. aeruginosa*
P. aeruginosa BWHPSA042	GCA_000520355.1	5.89339	66.50	AZZL01	3	5445	5311	*P. aeruginosa*
P. aeruginosa LES400	GCA_000583935.1	6.59112	66.30	NZ_CP006982.1	1	6117	5965	*P. aeruginosa*
P. aeruginosa ATCC 43390	GCA_001444925.1	6.6024	66.10	LJZL01	159	6149	5937	*P. aeruginosa*
P. sp. P179	GCA_000478485.2	6.89384	65.60	AQFO01	13	6343	6221	*P. aeruginosa*
P. aeruginosa PAO1	GCA_000006765.1	6.2644	66.60	NC_002516.2	-	5697	5572	*P. aeruginosa*
P. aeruginosa M10	GCA_000647655.1	6.01866	66.80	ATAG01	529	5673	5319	*P. aeruginosa*
P. aeruginosa ATCC 33352	GCA_001420265.1	6.21103	66.50	LJNY01	218	5808	5549	*P. aeruginosa*
P. aeruginosa 720_PAER	GCA_001067105.1	6.45421	66.30	JUVT01	359	6058	5813	*P. aeruginosa*
P. aeruginosa WH-SGI-V-07330	GCA_001454565.1	7.10838	65.80	LLVQ01	150	6630	6432	*P. aeruginosa*
P. aeruginosa P50_London_9_VIM_2_01_13	GCA_001181445.1	7.11645	65.80	CVWP01	192	6640	6480	*P. aeruginosa*
P. aeruginosa P22_London_17_VIM_2_06_10	GCA_001180845.1	7.02706	65.90	CVVI01	198	6482	6317	*P. aeruginosa*
P. aeruginosa BWHPSA022	GCA_000481265.1	6.91146	66.00	AXQG01	6	6405	6271	*P. aeruginosa*
P. aeruginosa BWHPSA046	GCA_000520275.1	6.90206	66.10	AZZH01	10	6404	6270	*P. aeruginosa*
P. aeruginosa AZPAE12413	GCA_000797125.1	6.74236	66.30	JUAB01	114	6234	5909	*P. aeruginosa*
P. denitrificans 1332_PDEN	GCA_001062775.1	7.03354	66.00	JVTH01	123	6451	6282	*P. aeruginosa*
P. aeruginosa BWHPSA017	GCA_000481365.1	6.93454	66.00	AXQL01	5	6349	6211	*P. aeruginosa*
P. aeruginosa 150_PMEN	GCA_001062835.1	6.20871	66.50	JVSG01	1755	6165	5162	*P. aeruginosa*
P. aeruginosa JD328	GCA_000506065.1	6.33026	66.50	AWZA01	1851	6190	5356	*P. aeruginosa*
P. aeruginosa JD310	GCA_000505925.1	6.40317	66.50	AWYR01	2062	6356	5415	*P. aeruginosa*
P. aeruginosa JD332	GCA_000506325.1	6.16612	66.60	AWZD01	2187	-	-	*P. aeruginosa*
P. aeruginosa WH-SGI-V-07486	GCA_001451605.1	6.8327	66.00	LLPS01	160	6382	6215	*P. aeruginosa*
P. aeruginosa WH-SGI-V-07646	GCA_001453485.1	6.80247	66.10	LLRR01	107	6298	6150	*P. aeruginosa*

P. aeruginosa AZPAE13879	GCA_000795685.1	6.94819	65.90	JTZD01	132	6485	6336	*P. aeruginosa*
P. aeruginosa NCGM 1900	GCA_000829275.1	6.81494	66.00	NZ_AP014622.1	-	6335	6206	*P. aeruginosa*
P. aeruginosa WH-SGI-V-07627	GCA_001452045.1	6.90942	66.10	LLQY01	93	6420	6273	*P. aeruginosa*
P. aeruginosa TSB 2	GCA_001374695.1	6.48965	-	CTCX01	77	-	-	*P. aeruginosa*
P. sp. 2_1_26	GCA_000233495.1	6.44748	66.10	ACWU01	51	5994	5588	*P. aeruginosa*
P. aeruginosa WH-SGI-V-07227	GCA_001450675.1	7.05976	66.00	LLMY01	124	6595	6400	*P. aeruginosa*
P. denitrificans 293_PDEN	GCA_001064395.1	6.83831	66.10	JVMP01	801	6625	6032	*P. aeruginosa*
P. aeruginosa WH-SGI-V-07185	GCA_001450625.1	7.19577	65.80	LLMP01	121	6711	6505	*P. aeruginosa*
P. nitroreducens Aramco J	GCA_000807755.1	7.33368	64.20	JUEH01	91	6915	6686	*P. nitroreducens*
P. nitroreducens HBP1	GCA_000518065.1	7.4082	64.20	AZRU01	212	7025	6197	*P. nitroreducens*
P. sp. 21	GCA_000955805.1	6.32619	65.60	JYOA01	42	5733	5646	*P. nitroreducens*
P. denitrificans ATCC 13867	GCA_000349845.1	5.69631	65.20	NC_020829.1	-	5086	4970	*P. nitroreducens*
P. citronellolis P3B5	GCA_001586155.1	6.95144	67.10	NZ_CP014158.1	-	6072	5936	*P. nitroreducens*
P. thermotolerans DSM 14292	GCA_000364625.1	3.7521	66.80	AQPA01	75	3525	3366	-
P. thermotolerans J53	GCA_000513835.1	3.75331	67.00	AZUT01	62	3516	3371	-

Genomes used for markers identification are highlighted in red.

[1] Phylogroups according to Garrido-Sanz et al. 2016.
