# Supplementary material for: Classification of Isolates from the Pseudomonas fluorescens Complex into Phylogenomic Groups Based in Group-Specific Markers
Source: Front Microbiol. 2017 Mar 15;8:413. doi: 10.3389/fmicb.2017.00413 (PMC5350142; doi:10.3389/fmicb.2017.00413)
Supplement: Supplementary Table 2 — Pseudomonas isolates used for blind test. [file Table2.PDF]

Supplementary Table 2. *Pseudomonas* isolates used for blind test

P. fluorescens **isolate name**	**Origin**	**Phylogroup affiliation**
Pseudomonas sp. EMC7	Endosphere, pumpkin	*P. fluorescens*
Pseudomonas sp. EMC11	Endosphere, pumpkin	*P. jessenii*
Pseudomonas sp. EMC3	Endosphere, pumpkin	*P. koreensis*
Pseudomonas sp. EMT2	Endosphere, tomato	*P. fluorescens*
Pseudomonas sp. EMT8	Endosphere, tomato	*P. fluorescens*
Pseudomonas sp. HFL1	Rhizosphere, lettuce	*P. jessenii*
Pseudomonas sp. HFL4	Rhizosphere, lettuce	*P. jessenii*
Pseudomonas sp. RMT2	Rhizosphere, tomato	*P. jessenii*
Pseudomonas sp. RMT4	Rhizosphere, tomato	*P. fluorescens*
Pseudomonas sp. RMT7	Rhizosphere, tomato	*P. koreensis*
Pseudomonas sp. RMT12	Rhizosphere, tomato	*P. fluorescens*
Pseudomonas sp. RMT1	Rhizosphere, tomato	*P. jessenii*
Pseudomonas sp. EMC5	Endosphere, pumpkin	*P. koreensis*
Pseudomonas sp. RMP9	Rhizosphere, pepper	*P. corrugata*
Pseudomonas sp. RMC9	Rhizosphere, pumpkin	*P. koreensis*
Pseudomonas sp. RMC8	Rhizosphere, pumpkin	*P. jessenii*
Pseudomonas sp. RMC4	Rhizosphere, pumpkin	*P. koreensis*
Pseudomonas sp. 7.3	Soil	*P. corrugata*
Pseudomonas sp. 3.2	Soil	*P. koreensis*
