# Supplementary material for: Classification of Isolates from the Pseudomonas fluorescens Complex into Phylogenomic Groups Based in Group-Specific Markers
Source: Front Microbiol. 2017 Mar 15;8:413. doi: 10.3389/fmicb.2017.00413 (PMC5350142; doi:10.3389/fmicb.2017.00413)
Supplement: Supplementary File 2 — Closest-related strain and phylogroup adscription according to 16S sequence identity. [file DataSheet2.PDF]

**Supplementary file 2**. Closest-related strain and phylogroup adscription according to 16S rDNA sequence identity.

| Isolate name (GenBank 16S rDNA accs. no.) | 16S% identity | 16S% cover. | Closest-related strain | Phylogroup |
|---|---|---|---|---|
| EMC3 (KY542119) | 99.74% | 100% | *P. fluorescens* Pf0-1 | *P. koreensis* |
| EMC5 (KY542268) | 99.58% | 100% | *P. fluorescens* Pf0-1 | *P. koreensis* |
| RMT7 (KY547838) | 99.62% | 99.75% | *P. fluorescens* Pf0-1 | *P. koreensis* |
| 3.2 (KY524298) | 99.74% | 100% | *P. fluorescens* Pf0-1 | *P. koreensis* |
| RMT1 (KY542269) | 98.74% | 99.62% | *P.* sp. GM33 | *P. jessenii* |
| RMT2 (KY547837) | 99.56% | 100% | *P.* sp. GM49 | *P. jessenii* |
| EMC7 (KY542270) | 99.55% | 99.85% | *P. veronii* 1YdBTEX2 | *P. fluorescens* |
| RMT4 (KY547831) | 99.30% | 99.86% | *P.* sp. Root569 | *P. fluorescens* |
| RMP9 (KY542122) | 99.94% | 100% | *P. fluorescens* F113 | *P. corrugata* |
| 7.3 (KY542120) | 99.94% | 100% | *P. brassicacearum* LBUM300 | *P. corrugata* |
