# Supplementary material for: Classification of Isolates from the Pseudomonas fluorescens Complex into Phylogenomic Groups Based in Group-Specific Markers
Source: Front Microbiol. 2017 Mar 15;8:413. doi: 10.3389/fmicb.2017.00413 (PMC5350142; doi:10.3389/fmicb.2017.00413)
Supplement: Supplementary File 3 — In silico PCR results. [file DataSheet3.PDF]

In silico PCR test for DGPF system vs. nt and wgs NCBI databases

as in March, 2016

No. #	Primer Name	Subject genome (NCBI ascc. No.)	*P. fluorescens* phylogroup	Primer mismatches	Primer 3' mismatches	Expected product lenght	Subject start	Subject end
	DGPf_0F			5	0	612	6084701	6084090
	DGPf_0R			1	0			
	DGPf_1F			-	-	0	-	-
	DGPf_1R			-	-			
	DGPf_2F			1	0	1001	2783112	2782112
	DGPf_2R			0	0			
	DGPf_3F			-	-	0	-	-
	DGPf_3R			-	-			
1	DGPf_4F	***P. protegens* CHA0**	*P. protegens*	0	0	1072	4724111	4725182
	DGPf_4R	NC_021237.1		0	0			
	DGPf_5F			-	-	0	-	-
	DGPf_5R			-	-			
	DGPf_6F			0	0	680	4195232	4194553
	DGPf_6R			0	0			
	DGPf_7F			-	-	0	-	-
	DGPf_7R			-	-			
	DGPf_8F			-	-	0	-	-
	DGPf_8R			-	-			
	DGPf_0F			4	0	612	6291704	6291093
	DGPf_0R			1	0			
	DGPf_1F			-	-	0	-	-
	DGPf_1R			-	-			
	DGPf_2F			1	0	1001	2735174	2734174
	DGPf_2R			0	0			
	DGPf_3F			-	-	0	-	-
	DGPf_3R			-	-			
2	DGPf_4F	***P. protegens* Pf-5**	*P. protegens*	0	0	1072	4782544	4783615
	DGPf_4R	NC_004129.6		0	0			
	DGPf_5F			-	-	0	-	-
	DGPf_5R			-	-			
	DGPf_6F			0	0	680	4263734	4263055
	DGPf_6R			0	0			
	DGPf_7F			-	-	0	-	-
	DGPf_7R			-	-			
	DGPf_8F			-	-	0	-	-
	DGPf_8R			-	-			
	DGPf_0F			4	0	612	6047439	6046828
	DGPf_0R			1	0			
	DGPf_1F			-	-	0	-	-
	DGPf_1R			-	-			
	DGPf_2F			1	0	1001	2758671	2757671
	DGPf_2R			0	0			
	DGPf_3F			-	-	0	-	-
	DGPf_3R			-	-			
3	DGPf_4F	***P. protegens* Cab57**	*P. protegens*	0	0	1073	4709015	4710087
	DGPf_4R	NZ_AP014522.1		0	0			
	DGPf_5F			-	-	0	-	-
	DGPf_5R			-	-			
	DGPf_6F			0	0	680	4243946	4243267
	DGPf_6R			0	0			
	DGPf_7F			-	-	0	-	-
	DGPf_7R			-	-			
	DGPf_8F			-	-	0	-	-
	DGPf_8R			-	-			
	DGPf_0F			1	0	612	25913	25302
	DGPf_0R			0	0			
	DGPf_1F			-	-	0	-	-
	DGPf_1R			-	-			
	DGPf_2F			1	0	1001	62885	61885
	DGPf_2R			0	0			
	DGPf_3F	***P. fluorescens* Wayne1**		-	-	0	-	-
	DGPf_3R	CADX01000061.1		-	-			
4	DGPf_4F	CADX01000065.1	*P. protegens*	0	0	1072	31468	32539
	DGPf_4R	CADX01000059.1		0	0			
	DGPf_5F	CADX01000088.1		-	-	0	-	-
	DGPf_5R			-	-			
	DGPf_6F			0	0	680	14206	14885
	DGPf_6R			0	0			
	DGPf_7F			-	-	0	-	-
	DGPf_7R			-	-			
	DGPf_8F			-	-	0	-	-
	DGPf_8R			-	-			
	DGPf_0F			1	0	612	6738	7349
	DGPf_0R			0	0			
	DGPf_1F			-	-	0	-	-
	DGPf_1R			-	-			
	DGPf_2F			1	0	1001	10272	9272
	DGPf_2R			0	0			
	DGPf_3F			-	-	0	-	-
	DGPf_3R	***P. fluorescens* NZI7**		-	-			
	DGPf_4F	AJXF01000078.1		0	0	1072	13583	12512
5	DGPf_4R	AJXF01000013.1	*P. protegens*	0	0			
	DGPf_5F	AJXF01000194.1		-	-	0	-	-
	DGPf_5R	AJXF01000051.1		-	-			

	DGPf_6F			0	0	680	5502	4823
	DGPf_6R			1	0			
	DGPf_7F			-	-	0	-	-
	DGPf_7R			-	-			
	DGPf_8F			-	-	0	-	-
	DGPf_8R			-	-			
	DGPf_0F			-	-	0	-	-
	DGPf_0R			-	-			
	DGPf_1F			-	-	0	-	-
	DGPf_1R			-	-			
	DGPf_2F			1	0	1001	8829	9829
	DGPf_2R			0	0			
	DGPf_3F			-	-	0	-	-
	DGPf_3R			-	-			
**6**	DGPf_4F	***P.* sp. PH1b**<br>AYMU01000001.1<br>AYMU01000022.1<br>AYMU01000014.1	*P. protegens*	0	0	1072	31080	30009
	DGPf_4R			0	0			
	DGPf_5F			-	-	0	-	-
	DGPf_5R			-	-			
	DGPf_6F			0	0	690	23817	24506
	DGPf_6R			0	0			
	DGPf_7F			-	-	0	-	-
	DGPf_7R			-	-			
	DGPf_8F			-	-	0	-	-
	DGPf_8R			-	-			
	DGPf_0F			2	0	612	6084582	6083971
	DGPf_0R			1	0			
	DGPf_1F			-	-	0	-	-
	DGPf_1R			-	-			
	DGPf_2F			1	0	1001	2698344	2697344
	DGPf_2R			0	0			
	DGPf_3F			-	-	0	-	-
	DGPf_3R			-	-			
**7**	DGPf_4F	***P.* sp. Os17**<br>AP014627.1	*P. protegens*	0	0	1072	4757136	4758207
	DGPf_4R			0	0			
	DGPf_5F			-	-	0	-	-
	DGPf_5R			-	-			
	DGPf_6F			0	0	660	4305385	4304726
	DGPf_6R			0	0			
	DGPf_7F			-	-	0	-	-
	DGPf_7R			-	-			
	DGPf_8F			-	-	0	-	-
	DGPf_8R			-	-			
	DGPf_0F			2	0	612	6007471	6006860
	DGPf_0R			1	0			
	DGPf_1F			-	-	0	-	-
	DGPf_1R			-	-			
	DGPf_2F			1	0	1001	2780123	2779123
	DGPf_2R			0	0			
	DGPf_3F			-	-	0	-	-
	DGPf_3R			-	-			
**8**	DGPf_4F	***P.* sp. St29**<br>AP014628.1	*P. protegens*	0	0	1072	4659317	4660388
	DGPf_4R			0	0			
	DGPf_5F			-	-	0	-	-
	DGPf_5R			-	-			
	DGPf_6F			0	0	680	4195946	4195267
	DGPf_6R			0	0			
	DGPf_7F			-	-	0	-	-
	DGPf_7R			-	-			
	DGPf_8F			-	-	0	-	-
	DGPf_8R			-	-			
	DGPf_0F			-	-	0	-	-
	DGPf_0R			-	-			
	DGPf_1F			-	-	0	-	-
	DGPf_1R			-	-			
	DGPf_2F			1	0	1001	96479	95479
	DGPf_2R			0	0			
	DGPf_3F			-	-	0	-	-
	DGPf_3R			-	-			
**9**	DGPf_4F	***P.* sp. CMAA1215**<br>AVOY01000202.1<br>AVOY01000042.1	*P. protegens*	0	0	1072	42482	43553
	DGPf_4R			0	0			
	DGPf_5F			-	-	0	-	-
	DGPf_5R			-	-			
	DGPf_6F			-	-	0	-	-
	DGPf_6R			-	-			
	DGPf_7F			-	-	0	-	-
	DGPf_7R			-	-			
	DGPf_8F			-	-	0	-	-
	DGPf_8R			-	-			
	DGPf_0F			-	-	0	-	-
	DGPf_0R			-	-			
	DGPf_1F			-	-	0	-	-
	DGPf_1R			-	-			
	DGPf_2F			1	0	1001	149628	148628
	DGPf_2R			0	0			
	DGPf_3F			-	-	0	-	-
	DGPf_3R			-	-			
**10**	DGPf_4F	***P. fluorescens* AU13852**<br>LCZC01000009.1<br>LCZC01000017.1	*P. protegens*	0	0	1072	89014	90085
	DGPf_4R			0	0			
	DGPf_5F			-	-	0	-	-
	DGPf_5R			-	-			

	DGPf_6F			-	-	0	-	-
	DGPf_6R			-	-			
	DGPf_7F			-	-	0	-	-
	DGPf_7R			-	-			
	DGPf_8F			-	-	0	-	-
	DGPf_8R			-	-			
	DGPf_0F			-	-	0	-	-
	DGPf_0R			-	-			
	DGPf_1F			-	-	0	-	-
	DGPf_1R			-	-			
	DGPf_2F			1	0	1001	148245	147245
	DGPf_2R			0	0			
	DGPf_3F			-	-	0	-	-
	DGPf_3R			-	-			
11	DGPf_4F	***P. fluorescens* AU20219**<br>LDET01000022.1<br>LDET01000031.1	*P. protegens*	0	0	1072	85185	86256
	DGPf_4R			0	0			
	DGPf_5F			-	-	0	-	-
	DGPf_5R			-	-			
	DGPf_6F			-	-	0	-	-
	DGPf_6R			-	-			
	DGPf_7F			-	-	0	-	-
	DGPf_7R			-	-			
	DGPf_8F			-	-	0	-	-
	DGPf_8R			-	-			
	DGPf_0F			-	-	0	-	-
	DGPf_0R			-	-			
	DGPf_1F			-	-	0	-	-
	DGPf_1R			-	-			
	DGPf_2F			1	0	1001	34129	33129
	DGPf_2R			0	0			
	DGPf_3F			-	-	0	-	-
	DGPf_3R			-	-			
12	DGPf_4F	***P. fluorescens* AU11706**<br>LCZB01000035.1<br>LCZB01000055.1	*P. protegens*	0	0	1072	48895	49966
	DGPf_4R			0	0			
	DGPf_5F			-	-	0	-	-
	DGPf_5R			-	-			
	DGPf_6F			-	-	0	-	-
	DGPf_6R			-	-			
	DGPf_7F			-	-	0	-	-
	DGPf_7R			-	-			
	DGPf_8F			-	-	0	-	-
	DGPf_8R			-	-			
	DGPf_0F			3	0	612	251697	251086
	DGPf_0R			1	0			
	DGPf_1F			-	-	0	-	-
	DGPf_1R			-	-			
	DGPf_2F			0	0	1001	554055	553055
	DGPf_2R			0	0			
	DGPf_3F			-	-	0	-	-
	DGPf_3R			-	-			
13	DGPf_4F	***P. protegens* K94.41**<br>LHUU01000013.1<br>LHUU01000006.1<br>LHUU01000010.1<br>LHUU01000009.1	*P. protegens*	0	0	1072	444081	445152
	DGPf_4R			0	0			
	DGPf_5F			-	-	0	-	-
	DGPf_5R			-	-			
	DGPf_6F			0	0	680	568965	568286
	DGPf_6R			0	0			
	DGPf_7F			-	-	0	-	-
	DGPf_7R			-	-			
	DGPf_8F			-	-	0	-	-
	DGPf_8R			-	-			
	DGPf_0F			4	0	612	212801	212190
	DGPf_0R			1	0			
	DGPf_1F			-	-	0	-	-
	DGPf_1R			-	-			
	DGPf_2F			1	0	1001	94263	95263
	DGPf_2R			0	0			
	DGPf_3F			-	-	0	-	-
	DGPf_3R			-	-			
14	DGPf_4F	***P. protegens* CMR5c**<br>LHUY01000007.1<br>LHUY01000010.1<br>LHUY01000024.1	*P. protegens*	0	0	1072	4558	5629
	DGPf_4R			0	0			
	DGPf_5F			-	-	0	-	-
	DGPf_5R			-	-			
	DGPf_6F			-	-	0	-	-
	DGPf_6R			-	-			
	DGPf_7F			-	-	0	-	-
	DGPf_7R			-	-			
	DGPf_8F			-	-	0	-	-
	DGPf_8R			-	-			
	PGN			4	0	612	250683	250072
	DGPf_0R			1	0			
	DGPf_1F			-	-	0	-	-
	DGPf_1R			-	-			
	DGPf_2F			1	0	1001	1394844	1393844
	DGPf_2R			0	0			
	DGPf_3F			-	-	0	-	-
	DGPf_3R			-	-			
15	DGPf_4F	***P. protegens* PGNR1**<br>LHUV01000009.1<br>LHUV01000005.1<br>LHUV01000006.1	*P. protegens*	0	0	1072	993700	994771
	DGPf_4R			0	0			
	DGPf_5F			-	-	0	-	-
	DGPf_5R			-	-			

	DGPf_6F			0	0	680	468177	467498
	DGPf_6R			0	0			
	DGPf_7F			-	-	0	-	-
	DGPf_7R			-	-			
	DGPf_8F			-	-	0	-	-
	DGPf_8R			-	-			
**16**	DGPf_0F	***P. chlororaphis* O6** AHOT01000028.1 AHOT01000002.1	*P. chlororaphis*	-	-	0	-	-
	DGPf_0R			-	-			
	DGPf_1F			-	-	0	-	-
	DGPf_1R			-	-			
	DGPf_2F			1	0	1001	158754	157754
	DGPf_2R			0	0			
	DGPf_3F			-	-	0	-	-
	DGPf_3R			-	-			
	DGPf_4F			-	-	0	-	-
	DGPf_4R			-	-			
	DGPf_5F			-	-	0	-	-
	DGPf_5R			-	-			
	DGPf_6F			-	-	0	-	-
	DGPf_6R			-	-			
	DGPf_7F			-	-	0	-	-
	DGPf_7R			-	-			
	DGPf_8F			0	0	661	186446	185786
	DGPf_8R			0	0			
**17**	DGPf_0F	***P. chlororaphis* YL-1** AWWJ01000051.1 AWWJ01000066.1	*P. chlororaphis*	-	-	0	-	-
	DGPf_0R			-	-			
	DGPf_1F			-	-	0	-	-
	DGPf_1R			-	-			
	DGPf_2F			1	0	1001	4129	5129
	DGPf_2R			0	0			
	DGPf_3F			-	-	0	-	-
	DGPf_3R			-	-			
	DGPf_4F			-	-	0	-	-
	DGPf_4R			-	-			
	DGPf_5F			-	-	0	-	-
	DGPf_5R			-	-			
	DGPf_6F			-	-	0	-	-
	DGPf_6R			-	-			
	DGPf_7F			-	-	0	-	-
	DGPf_7R			-	-			
	DGPf_8F			0	0	661	433366	432706
	DGPf_8R			0	0			
**18**	DGPf_0F	***P. chlororaphis* subsp. *chlororaphis* GP72** AHAY01000113.1 AHAY01000170.1	*P. chlororaphis*	-	-	0	-	-
	DGPf_0R			-	-			
	DGPf_1F			-	-	0	-	-
	DGPf_1R			-	-			
	DGPf_2F			1	0	1001	8076	9076
	DGPf_2R			0	0			
	DGPf_3F			-	-	0	-	-
	DGPf_3R			-	-			
	DGPf_4F			-	-	0	-	-
	DGPf_4R			-	-			
	DGPf_5F			-	-	0	-	-
	DGPf_5R			-	-			
	DGPf_6F			-	-	0	-	-
	DGPf_6R			-	-			
	DGPf_7F			-	-	0	-	-
	DGPf_7R			-	-			
	DGPf_8F			0	0	661	108886	108226
	DGPf_8R			0	0			
**19**	DGPf_0F	***P. chlororaphis* subsp. *aurantiaca* PB-St2** AYUD01000017.1 AYUD01000001.1	*P. chlororaphis*	-	-	0	-	-
	DGPf_0R			-	-			
	DGPf_1F			-	-	0	-	-
	DGPf_1R			-	-			
	DGPf_2F			1	0	1001	1145743	1144743
	DGPf_2R			0	0			
	DGPf_3F			-	-	0	-	-
	DGPf_3R			-	-			
	DGPf_4F			-	-	0	-	-
	DGPf_4R			-	-			
	DGPf_5F			-	-	0	-	-
	DGPf_5R			-	-			
	DGPf_6F			-	-	0	-	-
	DGPf_6R			-	-			
	DGPf_7F			-	-	0	-	-
	DGPf_7R			-	-			
	DGPf_8F			0	0	661	2275124	2274464
	DGPf_8R			0	0			
**20**	DGPf_0F	***P. chlororaphis* HT66** ATBG01000024.1	*P. chlororaphis*	-	-	0	-	-
	DGPf_0R			-	-			
	DGPf_1F			-	-	0	-	-
	DGPf_1R			-	-			
	DGPf_2F			2	0	1001	10135	11135
	DGPf_2R			0	0			
	DGPf_3F			-	-	0	-	-
	DGPf_3R			-	-			
	DGPf_4F			-	-	0	-	-
	DGPf_4R			-	-			
	DGPf_5F			-	-	0	-	-
	DGPf_5R			-	-			

	DGPf_6F			-	-	0	-	-
	DGPf_6R			-	-			
	DGPf_7F			-	-	0	-	-
	DGPf_7R			-	-			
	DGPf_8F			0	0	661	143586	142926
	DGPf_8R			0	0			
	DGPf_0F			-	-	0	-	-
	DGPf_0R			-	-			
	DGPf_1F			-	-	0	-	-
	DGPf_1R			-	-			
	DGPf_2F			1	0	1001	1712911	1711911
	DGPf_2R			0	0			
	DGPf_3F			-	-	0	-	-
	DGPf_3R	***P. chlororaphis* subsp.**		-	-			
**21**	DGPf_4F	***aureofaciens* 30-84**	*P. chlororaphis*	-	-	0	-	-
	DGPf_4R	AHHJ01000004.1		-	-			
	DGPf_5F	AHHJ01000011.1		-	-	0	-	-
	DGPf_5R			-	-			
	DGPf_6F			-	-	0	-	-
	DGPf_6R			-	-			
	DGPf_7F			-	-	0	-	-
	DGPf_7R			-	-			
	DGPf_8F			0	0	661	834033	833373
	DGPf_8R			0	0			
	DGPf_0F			-	-	0	-	-
	DGPf_0R			-	-			
	DGPf_1F			-	-	0	-	-
	DGPf_1R			-	-			
	DGPf_2F			1	0	1001	78350	77350
	DGPf_2R			0	0			
	DGPf_3F			-	-	0	-	-
	DGPf_3R	***P.* sp. GM17**		-	-			
**22**	DGPf_4F	AKJU01000096.1	*P. chlororaphis*	-	-	0	-	-
	DGPf_4R	AKJU01000177.1		-	-			
	DGPf_5F			-	-	0	-	-
	DGPf_5R			-	-			
	DGPf_6F			-	-	0	-	-
	DGPf_6R			-	-			
	DGPf_7F			-	-	0	-	-
	DGPf_7R			-	-			
	DGPf_8F			0	0	661	17136	17796
	DGPf_8R			0	0			
	DGPf_0F			-	-	0	-	-
	DGPf_0R			-	-			
	DGPf_1F			-	-	0	-	-
	DGPf_1R			-	-			
	DGPf_2F			1	0	1001	2484476	2483476
	DGPf_2R			0	0			
	DGPf_3F			-	-	0	-	-
	DGPf_3R			-	-			
**23**	DGPf_4F	***P. chlororaphis* PA23**	*P. chlororaphis*	-	-	0	-	-
	DGPf_4R	CP008696.1		-	-			
	DGPf_5F			-	-	0	-	-
	DGPf_5R			-	-			
	DGPf_6F			-	-	0	-	-
	DGPf_6R			-	-			
	DGPf_7F			-	-	0	-	-
	DGPf_7R			-	-			
	DGPf_8F			1	0	661	5614885	5614225
	DGPf_8R			1	0			
	DGPf_0F			-	-	0	-	-
	DGPf_0R			-	-			
	DGPf_1F			-	-	0	-	-
	DGPf_1R			-	-			
	DGPf_2F			0	0	1001	4110850	4111850
	DGPf_2R			0	0			
	DGPf_3F			-	-	0	-	-
	DGPf_3R	***P. chlororaphis* subsp.**		-	-			
**24**	DGPf_4F	***aurantiaca* JD37**	*P. chlororaphis*	-	-	0	-	-
	DGPf_4R	CP009290.1		-	-			
	DGPf_5F			-	-	0	-	-
	DGPf_5R			-	-			
	DGPf_6F			-	-	0	-	-
	DGPf_6R			-	-			
	DGPf_7F			-	-	0	-	-
	DGPf_7R			-	-			
	DGPf_8F			0	0	661	1329042	1329702
	DGPf_8R			1	0			
	DGPf_0F			-	-	0	-	-
	DGPf_0R			-	-			
	DGPf_1F			-	-	0	-	-
	DGPf_1R			-	-			
	DGPf_2F			0	0	1001	2623003	2622003
	DGPf_2R			0	0			
	DGPf_3F			-	-	0	-	-
	DGPf_3R			-	-			
**25**	DGPf_4F	***P.* sp. MRSN12121**	*P. chlororaphis*	-	-	0	-	-
	DGPf_4R	CP009290.1		-	-			
	DGPf_5F			-	-	0	-	-
	DGPf_5R			-	-			

	DGPf_6F			-	-	0	-	-
	DGPf_6R			-	-			
	DGPf_7F			-	-	0	-	-
	DGPf_7R			-	-			
	DGPf_8F			1	0	661	5637151	5636491
	DGPf_8R			0	0			
**26**	DGPf_0F	***P. chlororaphis* PCL1606**<br>CP011110.1	*P. chlororaphis*	-	-	0	-	-
	DGPf_0R			-	-			
	DGPf_1F			-	-	0	-	-
	DGPf_1R			-	-			
	DGPf_2F			0	0	1001	4089739	4090739
	DGPf_2R			0	0			
	DGPf_3F			-	-	0	-	-
	DGPf_3R			-	-			
	DGPf_4F			-	-	0	-	-
	DGPf_4R			-	-			
	DGPf_5F			-	-	0	-	-
	DGPf_5R			-	-			
	DGPf_6F			-	-	0	-	-
	DGPf_6R			-	-			
	DGPf_7F			-	-	0	-	-
	DGPf_7R			-	-			
	DGPf_8F			1	0	661	1283823	1284483
	DGPf_8R			1	0			
**27**	DGPf_0F	***P. chlororaphis* subsp. *aureofaciens* NBRC 3521**<br>BBQB01000013.1<br>BBQB01000006.1	*P. chlororaphis*	-	-	0	-	-
	DGPf_0R			-	-			
	DGPf_1F			-	-	0	-	-
	DGPf_1R			-	-			
	DGPf_2F			0	0	1001	261871	262871
	DGPf_2R			0	0			
	DGPf_3F			-	-	0	-	-
	DGPf_3R			-	-			
	DGPf_4F			-	-	0	-	-
	DGPf_4R			-	-			
	DGPf_5F			-	-	0	-	-
	DGPf_5R			-	-			
	DGPf_6F			-	-	0	-	-
	DGPf_6R			-	-			
	DGPf_7F			-	-	0	-	-
	DGPf_7R			-	-			
	DGPf_8F			0	0	661	220210	220870
	DGPf_8R			0	0			
**28**	DGPf_0F	***P. chlororaphis* subsp. *Chlororaphis* LMG 5004**<br>LHVC01000004.1<br>LHVC01000010.1	*P. chlororaphis*	-	-	0	-	-
	DGPf_0R			-	-			
	DGPf_1F			-	-	0	-	-
	DGPf_1R			-	-			
	DGPf_2F			0	0	1001	39991	38991
	DGPf_2R			0	0			
	DGPf_3F			-	-	0	-	-
	DGPf_3R			-	-			
	DGPf_4F			-	-	0	-	-
	DGPf_4R			-	-			
	DGPf_5F			-	-	0	-	-
	DGPf_5R			-	-			
	DGPf_6F			-	-	0	-	-
	DGPf_6R			-	-			
	DGPf_7F			-	-	0	-	-
	DGPf_7R			-	-			
	DGPf_8F			0	0	661	797302	796642
	DGPf_8R			0	0			
**29**	DGPf_0F	***P.* sp. Ag1**<br>AKVH01000049.1<br>AKVH01000102.1	*P. gessardii*	-	-	0	-	-
	DGPf_0R			-	-			
	DGPf_1F			-	-	0	-	-
	DGPf_1R			-	-			
	DGPf_2F			-	-	0	-	-
	DGPf_2R			-	-			
	DGPf_3F			-	-	0	-	-
	DGPf_3R			-	-			
	DGPf_4F			-	-	0	-	-
	DGPf_4R			-	-			
	DGPf_5F			0	0	1115	21292	20178
	DGPf_5R			0	0			
	DGPf_6F			0	0	680	136865	137544
	DGPf_6R			0	0			
	DGPf_7F			-	-	0	-	-
	DGPf_7R			-	-			
	DGPf_8F			-	-	0	-	-
	DGPf_8R			-	-			
**30**	DGPf_0F	***P. fluorescens* BBc6R8**<br>NZ_AKXH02000032.1<br>NZ_AKXH02000006.1	*P. gessardii*	-	-	0	-	-
	DGPf_0R			-	-			
	DGPf_1F			-	-	0	-	-
	DGPf_1R			-	-			
	DGPf_2F			-	-	0	-	-
	DGPf_2R			-	-			
	DGPf_3F			-	-	0	-	-
	DGPf_3R			-	-			
	DGPf_4F			-	-	0	-	-
	DGPf_4R			-	-			
	DGPf_5F			0	0	1115	116867	115753
	DGPf_5R			0	0			

	DGPf_6F			0	0	680	207	886
	DGPf_6R			0	0			
	DGPf_7F			-	-	0	-	-
	DGPf_7R			-	-			
	DGPf_8F			-	-	0	-	-
	DGPf_8R			-	-			
	DGPf_0F			-	-	0	-	-
	DGPf_0R			-	-			
	DGPf_1F			-	-	0	-	-
	DGPf_1R			-	-			
	DGPf_2F			-	-	0	-	-
	DGPf_2R			-	-			
	DGPf_3F			-	-	0	-	-
	DGPf_3R	***P.* sp. PAMC 26793**		-	-			
**31**	DGPf_4F	AMXG01000012.1	*P. gessardi*	-	-	0	-	-
	DGPf_4R	AMXG01000010.1		-	-			
	DGPf_5F			0	0	1115	112149	113263
	DGPf_5R			0	0			
	DGPf_6F			0	0	680	138430	137751
	DGPf_6R			0	0			
	DGPf_7F			-	-	0	-	-
	DGPf_7R			-	-			
	DGPf_8F			-	-	0	-	-
	DGPf_8R			-	-			
	DGPf_0F			-	-	0	-	-
	DGPf_0R			-	-			
	DGPf_1F			-	-	0	-	-
	DGPf_1R			-	-			
	DGPf_2F			-	-	0	-	-
	DGPf_2R			-	-			
	DGPf_3F			-	-	0	-	-
	DGPf_3R	***P.* sp. PAMC 25886**		-	-			
**32**	DGPf_4F	AHHC01000033.1	*P. gessardii*	-	-	0	-	-
	DGPf_4R	AHHC01000062.1		-	-			
	DGPf_5F			0	0	1115	25938	24824
	DGPf_5R			0	0			
	DGPf_6F			0	0	680	100104	99425
	DGPf_6R			0	0			
	DGPf_7F			-	-	0	-	-
	DGPf_7R			-	-			
	DGPf_8F			-	-	0	-	-
	DGPf_8R			-	-			
	DGPf_0F			-	-	0	-	-
	DGPf_0R			-	-			
	DGPf_1F			-	-	0	-	-
	DGPf_1R			-	-			
	DGPf_2F			-	-	0	-	-
	DGPf_2R			-	-			
	DGPf_3F			-	-	0	-	-
	DGPf_3R	***P.* sp. FH4**		-	-			
**33**	DGPf_4F	AOHN01000020.1	*P. gessardii*	-	-	0	-	-
	DGPf_4R	AOHN01000061.1		-	-			
	DGPf_5F			1	0	1115	12805	11691
	DGPf_5R			0	0			
	DGPf_6F			1	0	680	16946	16267
	DGPf_6R			0	0			
	DGPf_7F			-	-	0	-	-
	DGPf_7R			-	-			
	DGPf_8F			-	-	0	-	-
	DGPf_8R			-	-			
	DGPf_0F			-	-	0	-	-
	DGPf_0R			-	-			
	DGPf_1F			-	-	0	-	-
	DGPf_1R				-			
	DGPf_2F			-	-	0	-	-
	DGPf_2R			-	-			
	DGPf_3F			-	-	0	-	-
	DGPf_3R	***P. fluorescens* ATCC 17400**		-	-			
**34**	DGPf_4F	JENC01000010.1	*P. gessardii*	-	-	0	-	-
	DGPf_4R	JENC01000007.1		-	-			
	DGPf_5F			0	0	1115	180928	182042
	DGPf_5R			0	0			
	DGPf_6F			1	0	680	22501	23180
	DGPf_6R			2	0			
	DGPf_7F			-	-	0	-	-
	DGPf_7R			-	-			
	DGPf_8F			-	-	0	-	-
	DGPf_8R			-	-			
	DGPf_0F			-	-	0	-	-
	DGPf_0R			-	-			
	DGPf_1F			-	-	0	-	-
	DGPf_1R			-	-			
	DGPf_2F			-	-	0	-	-
	DGPf_2R			-	-			
	DGPf_3F			-	-	0	-	-
	DGPf_3R	***P.* sp. UK4**		-	-			
**35**	DGPf_4F	CP008896.1	*P. gessardii*	-	-	0	-	-
	DGPf_4R	CP008896.1		-	-			
	DGPf_5F			1	0	1115	441710	442824
	DGPf_5R			0	0			

	DGPf_6F			1	0	680	1586189	1585510
	DGPf_6R			0	0			
	DGPf_7F			-	-	0	-	-
	DGPf_7R			-	-			
	DGPf_8F			-		0	-	-
	DGPf_8R			-	-			
**36**	DGPf_0F	***P. fluorescens* GcM5-1A**<br>JJOE01000031.1<br>JJOE01000045.1	*P. gessardii*	-	-	0	-	-
	DGPf_0R			-	-			
	DGPf_1F			-	-	0	-	-
	DGPf_1R			-	-			
	DGPf_2F			-	-	0	-	-
	DGPf_2R			-	-			
	DGPf_3F			-	-	0	-	-
	DGPf_3R			-	-			
	DGPf_4F			-	-	0	-	-
	DGPf_4R			-	-			
	DGPf_5F			0	0	1115	31531	32645
	DGPf_5R			0	0			
	DGPf_6F			2	0	680	10728	10049
	DGPf_6R			0	0			
	DGPf_7F			-	-	0	-	-
	DGPf_7R			-	-			
	DGPf_8F			-	-	0	-	-
	DGPf_8R			-	-			
**37**	DGPf_0F	***P. fluorescens* ATCC 948**<br>JSFM01000046.1<br>JSFM01000042.1	*P. gessardii*	-	-	0	-	-
	DGPf_0R			-	-			
	DGPf_1F			-	-	0	-	-
	DGPf_1R			-	-			
	DGPf_2F			-	-	0	-	-
	DGPf_2R			-	-			
	DGPf_3F			-	-	0	-	-
	DGPf_3R			-	-			
	DGPf_4F			-	-	0	-	-
	DGPf_4R			-	-			
	DGPf_5F			0	0	1115	17885	18999
	DGPf_5R			0	0			
	DGPf_6F			0	0	680	10889	10210
	DGPf_6R			0	0			
	DGPf_7F			-	-	0	-	-
	DGPf_7R			-	-			
	DGPf_8F			-	-	0	-	-
	DGPf_8R			-	-			
**38**	DGPf_0F	***P. trivialis* IHBB745**<br>CP011507.1	*P. fluorescens*	-	-	0	-	-
	DGPf_0R			-	-			
	DGPf_1F			-	-	0	-	-
	DGPf_1R			-	-			
	DGPf_2F			-	-	0	-	-
	DGPf_2R			-	-			
	DGPf_3F			-	-	0	-	-
	DGPf_3R			-	-			
	DGPf_4F			-	-	0	-	-
	DGPf_4R			-	-			
	DGPf_5F			1	0	1115	311763	310649
	DGPf_5R			0	0			
	DGPf_6F			-	-	0	-	-
	DGPf_6R			-	-			
	DGPf_7F			1	0	745	762734	763478
	DGPf_7R			1	0			
	DGPf_8F			-	-	0	-	-
	DGPf_8R			-	-			
**39**	DGPf_0F	***P. fluorescens* SBW25**<br>AM181176.4	*P. fluorescens*	-	-	0	-	-
	DGPf_0R			-	-			
	DGPf_1F			-	-	0	-	-
	DGPf_1R			-	-			
	DGPf_2F			-	-	0	-	-
	DGPf_2R			-	-			
	DGPf_3F			-	-	0	-	-
	DGPf_3R			-	-			
	DGPf_4F			-	-	0	-	-
	DGPf_4R			-	-			
	DGPf_5F			0	0	1115	3066192	3067306
	DGPf_5R			0	0			
	DGPf_6F			-	-	0	-	-
	DGPf_6R			-	-			
	DGPf_7F			2	0	745	2491164	2490420
	DGPf_7R			1	0			
	DGPf_8F			-	-	0	-	-
	DGPf_8R			-	-			
**40**	DGPf_0F	***P. azotoformans* S4**<br>CP014546.1	*P. fluorescens*	-	-	0	-	-
	DGPf_0R			-	-			
	DGPf_1F			-	-	0	-	-
	DGPf_1R			-	-			
	DGPf_2F			-	-	0	-	-
	DGPf_2R			-	-			
	DGPf_3F			-	-	0	-	-
	DGPf_3R			-	-			
	DGPf_4F			-	-	0	-	-
	DGPf_4R			-	-			
	DGPf_5F			1	0	1115	4605226	4606340
	DGPf_5R			0	0			

	DGPf_6F			-	-	0	-	-
	DGPf_6R			-	-			
	DGPf_7F			1	0	745	3929183	3928439
	DGPf_7R			1	0			
	DGPf_8F			-	-	0	-	-
	DGPf_8R			-	-			
	DGPf_0F			-	-	0	-	-
	DGPf_0R			-	-			
	DGPf_1F			-	-	0	-	-
	DGPf_1R			-	-			
	DGPf_2F			-	-	0	-	-
	DGPf_2R			-	-			
	DGPf_3F			-	-	0	-	-
	DGPf_3R			-	-			
**41**	DGPf_4F	***P.* sp. TKP**	*P. fluorescens*	-	-	0	-	-
	DGPf_4R	CP006852.1		-	-			
	DGPf_5F			0	0	1115	3208437	3209551
	DGPf_5R			0	0			
	DGPf_6F			-	-	0	-	-
	DGPf_6R			-	-			
	DGPf_7F			1	0	745	2667877	2667133
	DGPf_7R			1	0			
	DGPf_8F			-	-	0	-	-
	DGPf_8R			-	-			
	DGPf_0F			-	-	0	-	-
	DGPf_0R			-	-			
	DGPf_1F			-	-	0	-	-
	DGPf_1R			-	-			
	DGPf_2F			-	-	0	-	-
	DGPf_2R			-	-			
	DGPf_3F			-	-	0	-	-
	DGPf_3R			-	-			
**42**	DGPf_4F	***P. simiae* WCS417**	*P. fluorescens*	-	-	0	-	-
	DGPf_4R	CP007637.1		-	-			
	DGPf_5F			0	0	1115	2811791	2812905
	DGPf_5R			0	0			
	DGPf_6F			-	-	0	-	-
	DGPf_6R			-	-			
	DGPf_7F			1	0	745	2335964	2335220
	DGPf_7R			1	0			
	DGPf_8F			-	-	0	-	-
	DGPf_8R			-	-			
	DGPf_0F			-	-	0	-	-
	DGPf_0R			-	-			
	DGPf_1F			-	-	0	-	-
	DGPf_1R			-	-			
	DGPf_2F			-	-	0	-	-
	DGPf_2R			-	-			
	DGPf_3F			-	-	0	-	-
	DGPf_3R			-	-			
**43**	DGPf_4F	***P. fluorescens* PCL1751**	*P. fluorescens*	-	-	0	-	-
	DGPf_4R	CP010896.1		-	-			
	DGPf_5F			0	0	1115	2739842	2740956
	DGPf_5R			0	0			
	DGPf_6F			-	-	0	-	-
	DGPf_6R			-	-			
	DGPf_7F			1	0	745	2265043	2264299
	DGPf_7R			1	0			
	DGPf_8F			-	-	0	-	-
	DGPf_8R			-	-			
	DGPf_0F			-	-	0	-	-
	DGPf_0R			-	-			
	DGPf_1F			-	-	0	-	-
	DGPf_1R			-	-			
	DGPf_2F			-	-	0	-	-
	DGPf_2R			-	-			
	DGPf_3F			-	-	0	-	-
	DGPf_3R			-	-			
**44**	DGPf_4F	***P. fluorescens* PICF7**	*P. fluorescens*	-	-	0	-	-
	DGPf_4R	CP005975.1		-	-			
	DGPf_5F			0	0	1115	5254351	5253237
	DGPf_5R			0	0			
	DGPf_6F			-	-	0	-	-
	DGPf_6R			-	-			
	DGPf_7F			1	0	745	5729391	5730135
	DGPf_7R			1	0			
	DGPf_8F			-	-	0	-	-
	DGPf_8R			-	-			
	DGPf_0F			-	-	0	-	-
	DGPf_0R			-	-			
	DGPf_1F			-	-	0	-	-
	DGPf_1R			-	-			
	DGPf_2F			-	-	0	-	-
	DGPf_2R			-	-			
	DGPf_3F			-	-	0	-	-
	DGPf_3R			-	-			
**45**	DGPf_4F	***P. fluorescens* LBUM223**	*P. fluorescens*	-	-	0	-	-
	DGPf_4R	CP011117.1		-	-			
	DGPf_5F			1	0	1115	132086	133200
	DGPf_5R			0	0			

	DGPf_6F			-	-	0	-	-
	DGPf_6R			-	-			
	DGPf_7F			4	0	745	6142900	6142156
	DGPf_7R			2	0			
	DGPf_8F			-	-	0	-	-
	DGPf_8R			-	-			
46	DGPf_0F	***P. poae* RE*1-1-14** CP004045.1	*P. fluorescens*	-	-	0	-	-
	DGPf_0R			-	-			
	DGPf_1F			-	-	0	-	-
	DGPf_1R			-	-			
	DGPf_2F			-	-	0	-	-
	DGPf_2R			-	-			
	DGPf_3F			-	-	0	-	-
	DGPf_3R			-	-			
	DGPf_4F			-	-	0	-	-
	DGPf_4R			-	-			
	DGPf_5F			0	0	1115	2294582	2293468
	DGPf_5R			0	0			
	DGPf_6F			-	-	0	-	-
	DGPf_6R			-	-			
	DGPf_7F			2	0	745	2559491	2560235
	DGPf_7R			1	0			
	DGPf_8F			-	-	0	-	-
	DGPf_8R			-	-			
47	DGPf_0F	***P.* sp. WCS374** CP007638.1	*P. fluorescens*	-	-	0	-	-
	DGPf_0R			-	-			
	DGPf_1F			-	-	0	-	-
	DGPf_1R			-	-			
	DGPf_2F			-	-	0	-	-
	DGPf_2R			-	-			
	DGPf_3F			-	-	0	-	-
	DGPf_3R			-	-			
	DGPf_4F			-	-	0	-	-
	DGPf_4R			-	-			
	DGPf_5F			0	0	1115	3237242	3236128
	DGPf_5R			0	0			
	DGPf_6F			-	-	0	-	-
	DGPf_6R			-	-			
	DGPf_7F			2	0	745	2405142	2404398
	DGPf_7R			1	0			
	DGPf_8F			-	-	0	-	-
	DGPf_8R			-	-			
48	DGPf_0F	***P. fluorescens* A506** CP003041.1	*P. fluorescens*	-	-	0	-	-
	DGPf_0R			-	-			
	DGPf_1F			-	-	0	-	-
	DGPf_1R			-	-			
	DGPf_2F			-	-	0	-	-
	DGPf_2R			-	-			
	DGPf_3F			-	-	0	-	-
	DGPf_3R			-	-			
	DGPf_4F			-	-	0	-	-
	DGPf_4R			-	-			
	DGPf_5F			0	0	1115	3242466	3241352
	DGPf_5R			0	0			
	DGPf_6F			-	-	0	-	-
	DGPf_6R			-	-			
	DGPf_7F			2	0	745	2393984	2393240
	DGPf_7R			1	0			
	DGPf_8F			-	-	0	-	-
	DGPf_8R			-	-			
49	DGPf_0F	***P. sp.* CBZ-4** ANNV01000016.1 ANNV01000168.1	*P. fluorescens*	-	-	0	-	-
	DGPf_0R			-	-			
	DGPf_1F			-	-	0	-	-
	DGPf_1R			-	-			
	DGPf_2F			-	-	0	-	-
	DGPf_2R			-	-			
	DGPf_3F			-	-	0	-	-
	DGPf_3R			-	-			
	DGPf_4F			-	-	0	-	-
	DGPf_4R			-	-			
	DGPf_5F			0	0	1115	45384	46498
	DGPf_5R			0	0			
	DGPf_6F			-	-	0	-	-
	DGPf_6R			-	-			
	DGPf_7F			1	0	745	8179	8923
	DGPf_7R			1	0			
	DGPf_8F			-	-	0	-	-
	DGPf_8R			-	-			
50	DGPf_0F	***P. extremaustralis* 14-3** AHIP01000060.1 AHIP01000029.1	*P. fluorescens*	-	-	0	-	-
	DGPf_0R			-	-			
	DGPf_1F			-	-	0	-	-
	DGPf_1R			-	-			
	DGPf_2F			-	-	0	-	-
	DGPf_2R			-	-			
	DGPf_3F			-	-	0	-	-
	DGPf_3R			-	-			
	DGPf_4F			-	-	0	-	-
	DGPf_4R			-	-			
	DGPf_5F			0	0	1114	160	1274
	DGPf_5R			0	0			

	DGPf_6F			-	-	0	-	-
	DGPf_6R			-	-			
	DGPf_7F			1	0	745	11147	10403
	DGPf_7R			1	0			
	DGPf_8F			-	-	0	-	-
	DGPf_8R			-	-			
	DGPf_0F			-	-	0	-	-
	DGPf_0R			-	-			
	DGPf_1F			-	-	0	-	-
	DGPf_1R			-	-			
	DGPf_2F			-	-	0	-	-
	DGPf_2R			-	-			
	DGPf_3F			-	-	0	-	-
	DGPf_3R			-	-			
**51**	DGPf_4F	***P. fluorescens*** **ICMP3636**<br>LKEI01000028.1<br>LKEI01000058.1	*P. fluorescens*	-	-	0	-	-
	DGPf_4R			-	-			
	DGPf_5F			0	0	1115	1383	269
	DGPf_5R			0	0			
	DGPf_6F			-	-	0	-	-
	DGPf_6R			-	-			
	DGPf_7F			1	0	745	122537	123281
	DGPf_7R			1	0			
	DGPf_8F			-	-	0	-	-
	DGPf_8R			-	-			
	DGPf_0F			-	-	0	-	-
	DGPf_0R			-	-			
	DGPf_1F			-	-	0	-	-
	DGPf_1R			-	-			
	DGPf_2F			-	-	0	-	-
	DGPf_2R			-	-			
	DGPf_3F			-	-	0	-	-
	DGPf_3R			-	-			
**52**	DGPf_4F	***P.*** **sp. RIT357**<br>JFYX01000016.1<br>JFYX01000007.1	*P. fluorescens*	-	-	0	-	-
	DGPf_4R			-	-			
	DGPf_5F			1	0	1115	7093	5979
	DGPf_5R			0	0			
	DGPf_6F			-	-	0	-	-
	DGPf_6R			-	-			
	DGPf_7F			2	0	745	35357	36101
	DGPf_7R			2	0			
	DGPf_8F			-	-	0	-	-
	DGPf_8R			-	-			
	DGPf_0F			-	-	0	-	-
	DGPf_0R			-	-			
	DGPf_1F			-	-	0	-	-
	DGPf_1R			-	-			
	DGPf_2F			-	-	0	-	-
	DGPf_2R			-	-			
	DGPf_3F			-	-	0	-	-
	DGPf_3R			-	-			
**53**	DGPf_4F	***P. veronii*** **R4**<br>JXWQ02000001.1	*P. fluorescens*	-	-	0	-	-
	DGPf_4R			-	-			
	DGPf_5F			0	0	1115	3139370	3140484
	DGPf_5R			0	0			
	DGPf_6F			-	-	0	-	-
	DGPf_6R			-	-			
	DGPf_7F			1	0	745	4399733	4400477
	DGPf_7R			1	0			
	DGPf_8F			-	-	0	-	-
	DGPf_8R			-	-			
	DGPf_0F			-	-	0	-	-
	DGPf_0R			-	-			
	DGPf_1F			-	-	0	-	-
	DGPf_1R			-	-			
	DGPf_2F			-	-	0	-	-
	DGPf_2R			-	-			
	DGPf_3F			-	-	0	-	-
	DGPf_3R			-	-			
**54**	DGPf_4F	***P. veronii*** **1YB2**<br>JGYI01000011.1<br>JGYI01000017.1	*P. fluorescens*	-	-	0	-	-
	DGPf_4R			-	-			
	DGPf_5F			0	0	1115	10334	9220
	DGPf_5R			0	0			
	DGPf_6F			-	-	0	-	-
	DGPf_6R			-	-			
	DGPf_7F			1	0	745	17760	17016
	DGPf_7R			2	0			
	DGPf_8F			-	-	0	-	-
	DGPf_8R			-	-			
	DGPf_0F			-	-	0	-	-
	DGPf_0R			-	-			
	DGPf_1F			-	-	0	-	-
	DGPf_1R			-	-			
	DGPf_2F			-	-	0	-	-
	DGPf_2R			-	-			
	DGPf_3F			-	-	0	-	-
	DGPf_3R			-	-			
**55**	DGPf_4F	***P. fluorescens*** **BS2**<br>AMZG01000051.1<br>AMZG01000016.1	*P. fluorescens*	-	-	0	-	-
	DGPf_4R			-	-			
	DGPf_5F			0	0	1115	6982	5868
	DGPf_5R			0	0			

	DGPf_6F			-	-	0	-	-
	DGPf_6R			-	-			
	DGPf_7F			0	0	745	59875	59131
	DGPf_7R			1	0			
	DGPf_8F			-	-	0	-	-
	DGPf_8R			-	-			
	DGPf_0F			-	-	0	-	-
	DGPf_0R			-	-			
	DGPf_1F			-	-	0	-	-
	DGPf_1R			-	-			
	DGPf_2F			-	-	0	-	-
	DGPf_2R			-	-			
	DGPf_3F			-	-	0	-	-
	DGPf_3R			-	-			
**56**	DGPf_4F	***P. veronii* 1YdBTEX2**<br>AOUH01000029.1<br>AOUH01000025.1	*P. fluorescens*	-	-	0	-	-
	DGPf_4R			-	-			
	DGPf_5F			0	0	1115	105263	106377
	DGPf_5R			0	0			
	DGPf_6F			-	-	0	-	-
	DGPf_6R			-	-			
	DGPf_7F			1	0	745	26863	27607
	DGPf_7R			1	0			
	DGPf_8F			-	-	0	-	-
	DGPf_8R			-	-			
	DGPf_0F			-	-	0	-	-
	DGPf_0R			-	-			
	DGPf_1F			-	-	0	-	-
	DGPf_1R			-	-			
	DGPf_2F			-	-	0	-	-
	DGPf_2R			-	-			
	DGPf_3F			-	-	0	-	-
	DGPf_3R			-	-			
**57**	DGPf_4F	***P. fluorescens* H14**<br>LACG01000001.1<br>LACG01000021.1	*P. fluorescens*	-	-	0	-	-
	DGPf_4R			-	-			
	DGPf_5F			0	0	1115	123804	124918
	DGPf_5R			0	0			
	DGPf_6F			-	-	0	-	-
	DGPf_6R			-	-			
	DGPf_7F			3	0	745	42345	43089
	DGPf_7R			1	0			
	DGPf_8F			-	-	0	-	-
	DGPf_8R			-	-			
	DGPf_0F			-	-	0	-	-
	DGPf_0R			-	-			
	DGPf_1F			-	-	0	-	-
	DGPf_1R			-	-			
	DGPf_2F			-	-	0	-	-
	DGPf_2R			-	-			
	DGPf_3F			-	-	0	-	-
	DGPf_3R			-	-			
**58**	DGPf_4F	***P. fluorescens* H21**<br>LACF01000010.1<br>LACF01000013.1	*P. fluorescens*	-	-	0	-	-
	DGPf_4R			-	-			
	DGPf_5F			0	0	1115	189348	188234
	DGPf_5R			0	0			
	DGPf_6F			-	-	0	-	-
	DGPf_6R			-	-			
	DGPf_7F			3	0	745	119942	119198
	DGPf_7R			1	0			
	DGPf_8F			-	-	0	-	-
	DGPf_8R			-	-			
	DGPf_0F			-	-	0	-	-
	DGPf_0R			-	-			
	DGPf_1F			-	-	0	-	-
	DGPf_1R			-	-			
	DGPf_2F			-	-	0	-	-
	DGPf_2R			-	-			
	DGPf_3F			-	-	0	-	-
	DGPf_3R			-	-			
**59**	DGPf_4F	***P. simiae* MEB105**<br>JXQT01000020.1<br>JXQT01000019.1	*P. fluorescens*	-	-	0	-	-
	DGPf_4R			-	-			
	DGPf_5F			0	0	1115	10676	9562
	DGPf_5R			0	0			
	DGPf_6F			-	-	0	-	-
	DGPf_6R			-	-			
	DGPf_7F			1	0	745	92090	91346
	DGPf_7R			1	0			
	DGPf_8F			-	-	0	-	-
	DGPf_8R			-	-			
	DGPf_0F			-	-	0	-	-
	DGPf_0R			-	-			
	DGPf_1F			-	-	0	-	-
	DGPf_1R			-	-			
	DGPf_2F			-	-	0	-	-
	DGPf_2R			-	-			
	DGPf_3F			-	-	0	-	-
	DGPf_3R			-	-			
**60**	DGPf_4F	***P. simiae* 2-36**<br>JRMC01000009.1<br>JRMC01000007.1	*P. fluorescens*	-	-	0	-	-
	DGPf_4R			-	-			
	DGPf_5F			0	0	1115	277028	278142
	DGPf_5R			0	0			

	DGPf_6F			-	-	0	-	-
	DGPf_6R			-	-			
	DGPf_7F			1	0	745	192237	192981
	DGPf_7R			1	0			
	DGPf_8F			-	-	0	-	-
	DGPf_8R			-	-			
**61**	DGPf_0F	***P.* sp. R81**<br>AHZN01000078.1<br>AHZN01000086.1	*P. fluorescens*	-	-	0	-	-
	DGPf_0R			-	-			
	DGPf_1F			-	-	0	-	-
	DGPf_1R			-	-			
	DGPf_2F			-	-	0	-	-
	DGPf_2R			-	-			
	DGPf_3F			-	-	0	-	-
	DGPf_3R			-	-			
	DGPf_4F			-	-	0	-	-
	DGPf_4R			-	-			
	DGPf_5F			0	0	1115	11254	10140
	DGPf_5R			0	0			
	DGPf_6F			-	-	0	-	-
	DGPf_6R			-	-			
	DGPf_7F			1	0	745	53085	53829
	DGPf_7R			1	0			
	DGPf_8F			-	-	0	-	-
	DGPf_8R			-	-			
**62**	DGPf_0F	***P. fluorescens* WH6**<br>AEAZ01000018.1<br>AEAZ01000013.1	*P. fluorescens*	-	-	0	-	-
	DGPf_0R			-	-			
	DGPf_1F			-	-	0	-	-
	DGPf_1R			-	-			
	DGPf_2F			-	-	0	-	-
	DGPf_2R			-	-			
	DGPf_3F			-	-	0	-	-
	DGPf_3R			-	-			
	DGPf_4F			-	-	0	-	-
	DGPf_4R			-	-			
	DGPf_5F			0	0	1115	183460	184574
	DGPf_5R			0	0			
	DGPf_6F			-	-	0	-	-
	DGPf_6R			-	-			
	DGPf_7F			1	0	745	118564	117820
	DGPf_7R			1	0			
	DGPf_8F			-	-	0	-	-
	DGPf_8R			-	-			
**63**	DGPf_0F	***P. fluorescens* ICMP 11288**<br>LKEF01000068.1<br>LKEF01000013.1	*P. fluorescens*	-	-	0	-	-
	DGPf_0R			-	-			
	DGPf_1F			-	-	0	-	-
	DGPf_1R			-	-			
	DGPf_2F			-	-	0	-	-
	DGPf_2R			-	-			
	DGPf_3F			-	-	0	-	-
	DGPf_3R			-	-			
	DGPf_4F			-	-	0	-	-
	DGPf_4R			-	-			
	DGPf_5F			0	0	1115	1960	3074
	DGPf_5R			0	0			
	DGPf_6F			-	-	0	-	-
	DGPf_6R			-	-			
	DGPf_7F			1	0	745	97708	96964
	DGPf_7R			1	0			
	DGPf_8F			-	-	0	-	-
	DGPf_8R			-	-			
**64**	DGPf_0F	***P. fluorescens* AU10973**<br>JRXV01000010.1<br>JRXV01000006.1	*P. fluorescens*	-	-	0	-	-
	DGPf_0R			-	-			
	DGPf_1F			-	-	0	-	-
	DGPf_1R			-	-			
	DGPf_2F			-	-	0	-	-
	DGPf_2R			-	-			
	DGPf_3F			-	-	0	-	-
	DGPf_3R			-	-			
	DGPf_4F			-	-	0	-	-
	DGPf_4R			-	-			
	DGPf_5F			0	0	1115	204453	205567
	DGPf_5R			0	0			
	DGPf_6F			-	-	0	-	-
	DGPf_6R			-	-			
	DGPf_7F			1	0	745	516769	516025
	DGPf_7R			1	0			
	DGPf_8F			-	-	0	-	-
	DGPf_8R			-	-			
**65**	DGPf_0F	***P. fluorescens* EGD-AQ6**<br>AVQG01000011.1<br>AVQG01000058.1	*P. fluorescens*	-	-	0	-	-
	DGPf_0R			-	-			
	DGPf_1F			-	-	0	-	-
	DGPf_1R			-	-			
	DGPf_2F			-	-	0	-	-
	DGPf_2R			-	-			
	DGPf_3F			-	-	0	-	-
	DGPf_3R			-	-			
	DGPf_4F			-	-	0	-	-
	DGPf_4R			-	-			
	DGPf_5F			0	0	1115	11520	10406
	DGPf_5R			0	0			

	DGPf_6F			-	-	0	-	-
	DGPf_6R			-	-			
	DGPf_7F			1	0	745	191439	192183
	DGPf_7R			1	0			
	DGPf_8F			-	-	0	-	-
	DGPf_8R			-	-			
66	DGPf_0F	***P.* sp. FH1** AOHM01000078.1 AOHM01000142.1	*P. fluorescens*	-	-	0	-	-
	DGPf_0R			-	-			
	DGPf_1F			-	-	0	-	-
	DGPf_1R			-	-			
	DGPf_2F			-	-	0	-	-
	DGPf_2R			-	-			
	DGPf_3F			-	-	0	-	-
	DGPf_3R			-	-			
	DGPf_4F			-	-	0	-	-
	DGPf_4R			-	-			
	DGPf_5F			1	1	1115	1304	190
	DGPf_5R			0	0			
	DGPf_6F			-	-	0	-	-
	DGPf_6R			-	-			
	DGPf_7F			1	0	745	48807	48063
	DGPf_7R			2	0			
	DGPf_8F			-	-	0	-	-
	DGPf_8R			-	-			
67	DGPf_0F	***P. veronii* DSM 11331** JYLL01000018.1 JYLL01000039.1	*P. fluorescens*	-	-	0	-	-
	DGPf_0R			-	-			
	DGPf_1F			-	-	0	-	-
	DGPf_1R			-	-			
	DGPf_2F			-	-	0	-	-
	DGPf_2R			-	-			
	DGPf_3F			-	-	0	-	-
	DGPf_3R			-	-			
	DGPf_4F			-	-	0	-	-
	DGPf_4R			-	-			
	DGPf_5F			0	0	1115	104690	105804
	DGPf_5R			0	0			
	DGPf_6F			-	-	0	-	-
	DGPf_6R			-	-			
	DGPf_7F			1	0	745	36442	37186
	DGPf_7R			1	0			
	DGPf_8F			-	-	0	-	-
	DGPf_8R			-	-			
68	DGPf_0F	***P.* sp. CHM02** JFCA01000009.1 JFCA01000003.1	*P. fluorescens*	-	-	0	-	-
	DGPf_0R			-	-			
	DGPf_1F			-	-	0	-	-
	DGPf_1R			-	-			
	DGPf_2F			-	-	0	-	-
	DGPf_2R			-	-			
	DGPf_3F			-	-	0	-	-
	DGPf_3R			-	-			
	DGPf_4F			-	-	0	-	-
	DGPf_4R			-	-			
	DGPf_5F			1	0	1115	64158	63044
	DGPf_5R			0	0			
	DGPf_6F			-	-	0	-	-
	DGPf_6R			-	-			
	DGPf_7F			1	0	745	143243	142499
	DGPf_7R			1	0			
	DGPf_8F			-	-	0	-	-
	DGPf_8R			-	-			
69	DGPf_0F	***P.* sp. KG01** LFMW01000012.1 LFMW01000039.1	*P. fluorescens*	-	-	0	-	-
	DGPf_0R			-	-			
	DGPf_1F			-	-	0	-	-
	DGPf_1R			-	-			
	DGPf_2F			-	-	0	-	-
	DGPf_2R			-	-			
	DGPf_3F			-	-	0	-	-
	DGPf_3R			-	-			
	DGPf_4F			-	-	0	-	-
	DGPf_4R			-	-			
	DGPf_5F			0	0	1115	568859	569973
	DGPf_5R			0	0			
	DGPf_6F			-	-	0	-	-
	DGPf_6R			-	-			
	DGPf_7F			1	0	745	238886	239630
	DGPf_7R			1	0			
	DGPf_8F			-	-	0	-	-
	DGPf_8R			-	-			
70	DGPf_0F	***P. orientalis* DSM 17489** JYLM01000009.1 JYLM01000003.1	*P. fluorescens*	-	-	0	-	-
	DGPf_0R			-	-			
	DGPf_1F			-	-	0	-	-
	DGPf_1R			-	-			
	DGPf_2F			-	-	0	-	-
	DGPf_2R			-	-			
	DGPf_3F			-	-	0	-	-
	DGPf_3R			-	-			
	DGPf_4F			-	-	0	-	-
	DGPf_4R			-	-			
	DGPf_5F			3	0	1115	234106	235220
	DGPf_5R			1	0			

	DGPf_6F			-	-	0	-	-
	DGPf_6R			-	-			
	DGPf_7F			1	0	745	21474	20730
	DGPf_7R			1	0			
	DGPf_8F			-	-	0	-	-
	DGPf_8R			-	-			
71	DGPf_0F	***P. fluorescens*** **AU14705**<br>JRYA01000015.1<br>JRYA01000013.1	*P. fluorescens*	-	-	0	-	-
	DGPf_0R			-	-			
	DGPf_1F			-	-	0	-	-
	DGPf_1R			-	-			
	DGPf_2F			-	-	0	-	-
	DGPf_2R			-	-			
	DGPf_3F			-	-	0	-	-
	DGPf_3R			-	-			
	DGPf_4F			-	-	0	-	-
	DGPf_4R			-	-			
	DGPf_5F			0	0	1115	241988	243102
	DGPf_5R			0	0			
	DGPf_6F			-	-	0	-	-
	DGPf_6R			-	-			
	DGPf_7F			0	0	745	184350	183606
	DGPf_7R			1	0			
	DGPf_8F			-	-	0	-	-
	DGPf_8R			-	-			
72	DGPf_0F	***P. fluorescens*** **NZ052**<br>AJXH01000436.1<br>AJXH01000364.1	*P. fluorescens*	-	-	0	-	-
	DGPf_0R			-	-			
	DGPf_1F			-	-	0	-	-
	DGPf_1R			-	-			
	DGPf_2F			-	-	0	-	-
	DGPf_2R			-	-			
	DGPf_3F			-	-	0	-	-
	DGPf_3R			-	-			
	DGPf_4F			-	-	0	-	-
	DGPf_4R			-	-			
	DGPf_5F			0	0	1115	6258	5144
	DGPf_5R			0	0			
	DGPf_6F			-	-	0	-	-
	DGPf_6R			-	-			
	DGPf_7F			1	0	745	6258	7002
	DGPf_7R			0	0			
	DGPf_8F			-	-	0	-	-
	DGPf_8R			-	-			
73	DGPf_0F	***P.*** **sp. Leaf15**<br>LMKI01000027.1<br>LMKI01000036.1	*P. fluorescens*	-	-	0	-	-
	DGPf_0R			-	-			
	DGPf_1F			-	-	0	-	-
	DGPf_1R			-	-			
	DGPf_2F			-	-	0	-	-
	DGPf_2R			-	-			
	DGPf_3F			-	-	0	-	-
	DGPf_3R			-	-			
	DGPf_4F			-	-	0	-	-
	DGPf_4R			-	-			
	DGPf_5F			0	0	1115	10243	9129
	DGPf_5R			0	0			
	DGPf_6F			-	-	0	-	-
	DGPf_6R			-	-			
	DGPf_7F			1	0	745	158626	157882
	DGPf_7R			1	0			
	DGPf_8F			-	-	0	-	-
	DGPf_8R			-	-			
74	DGPf_0F	***P.*** **sp. LAMO17WK12:I2**<br>AZVU01000015.1<br>AZVU01000001.1	*P. fluorescens*	-	-	0	-	-
	DGPf_0R			-	-			
	DGPf_1F			-	-	0	-	-
	DGPf_1R			-	-			
	DGPf_2F			-	-	0	-	-
	DGPf_2R			-	-			
	DGPf_3F			-	-	0	-	-
	DGPf_3R			-	-			
	DGPf_4F			-	-	0	-	-
	DGPf_4R			-	-			
	DGPf_5F			1	0	1115	10381	9267
	DGPf_5R			0	0			
	DGPf_6F			-	-	0	-	-
	DGPf_6R			-	-			
	DGPf_7F			1	0	745	546979	546235
	DGPf_7R			1	0			
	DGPf_8F			-	-	0	-	-
	DGPf_8R			-	-			
75	DGPf_0F	***P. fluorescens*** **LMG 5329**<br>ASGY01000196.1<br>ASGY01000052.1	*P. fluorescens*	-	-	0	-	-
	DGPf_0R			-	-			
	DGPf_1F			-	-	0	-	-
	DGPf_1R			-	-			
	DGPf_2F			-	-	0	-	-
	DGPf_2R			-	-			
	DGPf_3F			-	-	0	-	-
	DGPf_3R			-	-			
	DGPf_4F			-	-	0	-	-
	DGPf_4R			-	-			
	DGPf_5F			1	1	1115	5909	4795
	DGPf_5R			0	0			

	DGPf_6F			-	-	0	-	-
	DGPf_6R			-	-			
	DGPf_7F			1	0	745	22147	22891
	DGPf_7R			1	0			
	DGPf_8F			-	-	0	-	-
	DGPf_8R			-	-			
**76**	DGPf_0F	***P.* sp. 2-92 (2010)**<br>AYTD01000010.1<br>AYTD01000006.1	*P. fluorescens*	-	-	0	-	-
	DGPf_0R			-	-			
	DGPf_1F			-	-	0	-	-
	DGPf_1R			-	-			
	DGPf_2F			-	-	0	-	-
	DGPf_2R			-	-			
	DGPf_3F			-	-	0	-	-
	DGPf_3R			-	-			
	DGPf_4F			-	-	0	-	-
	DGPf_4R			-	-			
	DGPf_5F			0	0	1115	137626	138740
	DGPf_5R			0	0			
	DGPf_6F			-	-	0	-	-
	DGPf_6R			-	-			
	DGPf_7F			1	0	745	223510	222766
	DGPf_7R			1	0			
	DGPf_8F			-	-	0	-	-
	DGPf_8R			-	-			
**77**	DGPf_0F	***P.* sp. Root9**<br>LMIY01000006.1	*P. fluorescens*	-	-	0	-	-
	DGPf_0R			-	-			
	DGPf_1F			-	-	0	-	-
	DGPf_1R			-	-			
	DGPf_2F			-	-	0	-	-
	DGPf_2R			-	-			
	DGPf_3F			-	-	0	-	-
	DGPf_3R			-	-			
	DGPf_4F			-	-	0	-	-
	DGPf_4R			-	-			
	DGPf_5F			0	0	1115	504362	505476
	DGPf_5R			0	0			
	DGPf_6F			-	-	0	-	-
	DGPf_6R			-	-			
	DGPf_7F			1	0	745	37440	36696
	DGPf_7R			1	0			
	DGPf_8F			-	-	0	-	-
	DGPf_8R			-	-			
**78**	DGPf_0F	***P.* sp. Eur1 9.41**<br>JQLM01000002.1	*P. fluorescens*	-	-	0	-	-
	DGPf_0R			-	-			
	DGPf_1F			-	-	0	-	-
	DGPf_1R			-	-			
	DGPf_2F			-	-	0	-	-
	DGPf_2R			-	-			
	DGPf_3F			-	-	0	-	-
	DGPf_3R			-	-			
	DGPf_4F			-	-	0	-	-
	DGPf_4R			-	-			
	DGPf_5F			1	0	1115	100916	99802
	DGPf_5R			0	0			
	DGPf_6F			-	-	0	-	-
	DGPf_6R			-	-			
	DGPf_7F			1	0	745	529119	529863
	DGPf_7R			1	0			
	DGPf_8F			-	-	0	-	-
	DGPf_8R			-	-			
**79**	DGPf_0F	***P.* sp. ARP3**<br>LEKF01000007.1<br>LEKF01000019.1	*P. fluorescens*	-	-	0	-	-
	DGPf_0R			-	-			
	DGPf_1F			-	-	0	-	-
	DGPf_1R			-	-			
	DGPf_2F			-	-	0	-	-
	DGPf_2R			-	-			
	DGPf_3F			-	-	0	-	-
	DGPf_3R			-	-			
	DGPf_4F			-	-	0	-	-
	DGPf_4R			-	-			
	DGPf_5F			0	0	1115	217054	218168
	DGPf_5R			0	0			
	DGPf_6F			-	-	0	-	-
	DGPf_6R			-	-			
	DGPf_7F			3	0	745	33946	34690
	DGPf_7R			1	0			
	DGPf_8F			-	-	0	-	-
	DGPf_8R			-	-			
**80**	DGPf_0F	***P. fluorescens* AU12271**<br>JRYP01000007.1	*P. fluorescens*	-	-	0	-	-
	DGPf_0R			-	-			
	DGPf_1F			-	-	0	-	-
	DGPf_1R			-	-			
	DGPf_2F			-	-	0	-	-
	DGPf_2R			-	-			
	DGPf_3F			-	-	0	-	-
	DGPf_3R			-	-			
	DGPf_4F			-	-	0	-	-
	DGPf_4R			-	-			
	DGPf_5F			0	0	1115	220546	221660
	DGPf_5R			0	0			

	DGPf_6F			-	-	0	-	-
	DGPf_6R			-	-			
	DGPf_7F			3	0	745	332150	331406
	DGPf_7R			1	0			
	DGPf_8F			-	-	0	-	-
	DGPf_8R			-	-			
	DGPf_0F			-	-	0	-	-
	DGPf_0R			-	-			
	DGPf_1F			-	-	0	-	-
	DGPf_1R			-	-			
	DGPf_2F			-	-	0	-	-
	DGPf_2R			-	-			
	DGPf_3F			-	-	0	-	-
	DGPf_3R	**_P. fluorescens_ AU11518**		-	-			
	DGPf_4F			-	-	0	-	-
81	DGPf_4R	JRXW01000017.1	*P. fluorescens*	-	-			
	DGPf_5F	JRXW01000013.1		0	0	1115	217572	218686
	DGPf_5R			0	0			
	DGPf_6F			-	-	0	-	-
	DGPf_6R			-	-			
	DGPf_7F			3	0	745	28199	27455
	DGPf_7R			1	0			
	DGPf_8F			-	-	0	-	-
	DGPf_8R			-	-			
	DGPf_0F			-	-	0	-	-
	DGPf_0R			-	-			
	DGPf_1F			-	-	0	-	-
	DGPf_1R			-	-			
	DGPf_2F			-	-	0	-	-
	DGPf_2R			-	-			
	DGPf_3F			-	-	0	-	-
	DGPf_3R	**_P. fluorescens_ AU2989**		-	-			
	DGPf_4F			-	-	0	-	-
82	DGPf_4R	JRXT01000013.1	*P. fluorescens*	-	-			
	DGPf_5F	JRXT01000012.1		0	0	1115	226090	227204
	DGPf_5R			0	0			
	DGPf_6F			-	-	0	-	-
	DGPf_6R			-	-			
	DGPf_7F			3	0	745	77462	76718
	DGPf_7R			2	0			
	DGPf_8F			-	-	0	-	-
	DGPf_8R			-	-			
	DGPf_0F			-	-	0	-	-
	DGPf_0R			-	-			
	DGPf_1F			-	-	0	-	-
	DGPf_1R			-	-			
	DGPf_2F			-	-	0	-	-
	DGPf_2R			-	-			
	DGPf_3F			-	-	0	-	-
	DGPf_3R	**_P. fluorescens_ PA4C2**		-	-			
	DGPf_4F			-	-	0	-	-
83	DGPf_4R	AXDA01000030.1	*P. fluorescens*	-	-			
	DGPf_5F	AXDA01000019.1		0	0	1115	66799	67913
	DGPf_5R			0	0			
	DGPf_6F			-	-	0	-	-
	DGPf_6R			-	-			
	DGPf_7F			2	0	745	17059	16315
	DGPf_7R			1	0			
	DGPf_8F			-	-	0	-	-
	DGPf_8R			-	-			
	DGPf_0F			-	-	0	-	-
	DGPf_0R			-	-			
	DGPf_1F			-	-	0	-	-
	DGPf_1R			-	-			
	DGPf_2F			-	-	0	-	-
	DGPf_2R			-	-			
	DGPf_3F			-	-	0	-	-
	DGPf_3R	**_P. fluorescens_ NZ007**		-	-			
	DGPf_4F			-	-	0	-	-
84	DGPf_4R	AKBR01000692.1	*P. fluorescens*	-	-			
	DGPf_5F	AKBR01000293.1		0	0	1115	6717	5603
	DGPf_5R			0	0			
	DGPf_6F			-	-	0	-	-
	DGPf_6R			-	-			
	DGPf_7F			1	0	745	5750	6494
	DGPf_7R			1	0			
	DGPf_8F			-	-	0	-	-
	DGPf_8R			-	-			
	DGPf_0F			-	-	0	-	-
	DGPf_0R			-	-			
	DGPf_1F			-	-	0	-	-
	DGPf_1R			-	-			
	DGPf_2F			-	-	0	-	-
	DGPf_2R			-	-			
	DGPf_3F			-	-	0	-	-
	DGPf_3R	**_P._ sp. Root569**		-	-			
	DGPf_4F			-	-	0	-	-
85	DGPf_4R	LMGQ01000004.1	*P. fluorescens*	-	-			
	DGPf_5F	LMGQ01000006.1		1	0	1115	116299	117413
	DGPf_5R			0	0			

	DGPf_6F			-	-	0	-	-
	DGPf_6R			-	-			
	DGPf_7F			4	0	745	74457	75201
	DGPf_7R			0	0			
	DGPf_8F			-	-	0	-	-
	DGPf_8R			-	-			
	DGPf_0F			-	-	0	-	-
	DGPf_0R			-	-			
	DGPf_1F			-	-	0	-	-
	DGPf_1R			-	-			
	DGPf_2F			-	-	0	-	-
	DGPf_2R			-	-			
	DGPf_3F			-	-	0	-	-
	DGPf_3R			-	-			
**86**	DGPf_4F	***P.* sp. ABAC21**<br>LKBL01001684.1<br>LKBL01001263.1	*P. fluorescens*	-	-	0	-	-
	DGPf_4R			-	-			
	DGPf_5F			0	0	1115	4356	5470
	DGPf_5R			0	0			
	DGPf_6F			-	-	0	-	-
	DGPf_6R			-	-			
	DGPf_7F			2	0	745	3331	2587
	DGPf_7R			1	0			
	DGPf_8F			-	-	0	-	-
	DGPf_8R			-	-			
	DGPf_0F			-	-	0	-	-
	DGPf_0R			-	-			
	DGPf_1F			-	-	0	-	-
	DGPf_1R			-	-			
	DGPf_2F			-	-	0	-	-
	DGPf_2R			-	-			
	DGPf_3F			-	-	0	-	-
	DGPf_3R			-	-			
**87**	DGPf_4F	***P. trivialis* DSM 14937**<br>JYLK01000013.1<br>JYLK01000004.1	*P. fluorescens*	-	-	0	-	-
	DGPf_4R			-	-			
	DGPf_5F			0	0	1115	158654	157540
	DGPf_5R			0	0			
	DGPf_6F			-	-	0	-	-
	DGPf_6R			-	-			
	DGPf_7F			2	0	745	160036	160780
	DGPf_7R			1	0			
	DGPf_8F			-	-	0	-	-
	DGPf_8R			-	-			
	DGPf_0F			-	-	0	-	-
	DGPf_0R			-	-			
	DGPf_1F			-	-	0	-	-
	DGPf_1R			-	-			
	DGPf_2F			-	-	0	-	-
	DGPf_2R			-	-			
	DGPf_3F			-	-	0	-	-
	DGPf_3R			-	-			
**88**	DGPf_4F	***P. poae* DSM 14936**<br>JYLI01000001.1<br>JYLI01000011.1	*P. fluorescens*	-	-	0	-	-
	DGPf_4R			-	-			
	DGPf_5F			1	0	1115	354698	353584
	DGPf_5R			0	0			
	DGPf_6F			-	-	0	-	-
	DGPf_6R			-	-			
	DGPf_7F			2	0	745	159911	160655
	DGPf_7R			1	0			
	DGPf_8F			-	-	0	-	-
	DGPf_8R			-	-			
	DGPf_0F			-	-	0	-	-
	DGPf_0R			-	-			
	DGPf_1F			-	-	0	-	-
	DGPf_1R			-	-			
	DGPf_2F			-	-	0	-	-
	DGPf_2R			-	-			
	DGPf_3F			-	-	0	-	-
	DGPf_3R			-	-			
**89**	DGPf_4F	***P. libanensis* DSM 17149**<br>JYLH01000002.1<br>JYLH01000006.1	*P. fluorescens*	-	-	0	-	-
	DGPf_4R			-	-			
	DGPf_5F			1	0	1115	247671	246557
	DGPf_5R			0	0			
	DGPf_6F			-	-	0	-	-
	DGPf_6R			-	-			
	DGPf_7F			3	0	745	262596	261852
	DGPf_7R			0	0			
	DGPf_8F			-	-	0	-	-
	DGPf_8R			-	-			
	DGPf_0F			-	-	0	-	-
	DGPf_0R			-	-			
	DGPf_1F			-	-	0	-	-
	DGPf_1R			-	-			
	DGPf_2F			-	-	0	-	-
	DGPf_2R			-	-			
	DGPf_3F			-	-	0	-	-
	DGPf_3R			-	-			
**90**	DGPf_4F	***P. fluorescens* AU14917**<br>JRXY01000019.1<br>JRXY01000015.1	*P. fluorescens*	-	-	0	-	-
	DGPf_4R			-	-			
	DGPf_5F			0	0	1115	353026	354140
	DGPf_5R			0	0			

	DGPf_6F			-	-	0	-	-
	DGPf_6R			-	-			
	DGPf_7F			1	0	745	65733	64989
	DGPf_7R			1	0			
	DGPf_8F			-	-	0	-	-
	DGPf_8R			-	-			
	DGPf_0F			-	-	0	-	-
	DGPf_0R			-	-			
	DGPf_1F			-	-	0	-	-
	DGPf_1R			-	-			
	DGPf_2F			-	-	0	-	-
	DGPf_2R			-	-			
	DGPf_3F			-	-	0	-	-
	DGPf_3R			-	-			
**91**	DGPf_4F	***P. rhodesiae* FF9**<br>CCYI01000014.1	*P. fluorescens*	-	-	0	-	-
	DGPf_4R			-	-			
	DGPf_5F			0	0	1115	613874	614988
	DGPf_5R			0	0			
	DGPf_6F			-	-	0	-	-
	DGPf_6R			-	-			
	DGPf_7F			3	0	745	237730	236986
	DGPf_7R			1	0			
	DGPf_8F			-	-	0	-	-
	DGPf_8R			-	-			
	DGPf_0F			-	-	0	-	-
	DGPf_0R			-	-			
	DGPf_1F			-	-	0	-	-
	DGPf_1R			-	-			
	DGPf_2F			-	-	0	-	-
	DGPf_2R			-	-			
	DGPf_3F			-	-	0	-	-
	DGPf_3R			-	-			
**92**	DGPf_4F	***P. libanensis* RIT-PI-g**<br>LHOY01000024.1<br>LHOY01000006.1	*P. fluorescens*	-	-	0	-	-
	DGPf_4R			-	-			
	DGPf_5F			1	0	1115	7542	6428
	DGPf_5R			0	0			
	DGPf_6F			-	-	0	-	-
	DGPf_6R			-	-			
	DGPf_7F			4	0	745	76714	77458
	DGPf_7R			0	0			
	DGPf_8F			-	-	0	-	-
	DGPf_8R			-	-			
	DGPf_0F			-	-	0	-	-
	DGPf_0R			-	-			
	DGPf_1F			-	-	0	-	-
	DGPf_1R			-	-			
	DGPf_2F			-	-	0	-	-
	DGPf_2R			-	-			
	DGPf_3F			-	-	0	-	-
	DGPf_3R			-	-			
**93**	DGPf_4F	***P.* sp. CF150**<br>ATLQ01000014.1<br>ATLQ01000095.1	*P. fluorescens*	-	-	0	-	-
	DGPf_4R			-	-			
	DGPf_5F			0	0	1115	3515	4629
	DGPf_5R			0	0			
	DGPf_6F			-	-	0	-	-
	DGPf_6R			-	-			
	DGPf_7F			1	0	745	171630	170886
	DGPf_7R			1	0			
	DGPf_8F			-	-	0	-	-
	DGPf_8R			-	-			
	DGPf_0F			-	-	0	-	-
	DGPf_0R			-	-			
	DGPf_1F			-	-	0	-	-
	DGPf_1R			-	-			
	DGPf_2F			-	-	0	-	-
	DGPf_2R			-	-			
	DGPf_3F			-	-	0	-	-
	DGPf_3R			-	-			
**94**	DGPf_4F	***P. fluorescens* BRIP34879**<br>AMZW01000057.1<br>AMZW01000036.1	*P. fluorescens*	-	-	0	-	-
	DGPf_4R			-	-			
	DGPf_5F			0	0	1115	57411	58525
	DGPf_5R			0	0			
	DGPf_6F			-	-	0	-	-
	DGPf_6R			-	-			
	DGPf_7F			2	0	745	25026	24282
	DGPf_7R			1	0			
	DGPf_8F			-	-	0	-	-
	DGPf_8R			-	-			
	DGPf_0F			-	-	0	-	-
	DGPf_0R			-	-			
	DGPf_1F			-	-	0	-	-
	DGPf_1R			-	-			
	DGPf_2F			-	-	0	-	-
	DGPf_2R			-	-			
	DGPf_3F			-	-	0	-	-
	DGPf_3R			-	-			
**95**	DGPf_4F	***P. fluorescens* FH5**<br>AOJA01000006.1<br>AOJA01000060.1	*P. fluorescens*	-	-	0	-	-
	DGPf_4R			-	-			
	DGPf_5F			0	0	1115	7288	6174
	DGPf_5R			0	0			

	DGPf_6F			-	-	0	-	-
	DGPf_6R			-	-			
	DGPf_7F			2	0	745	107469	106725
	DGPf_7R			1	0			
	DGPf_8F			-	-	0	-	-
	DGPf_8R			-	-			
	DGPf_0F			-	-	0	-	-
	DGPf_0R			-	-			
	DGPf_1F			-	-	0	-	-
	DGPf_1R			-	-			
	DGPf_2F			-	-	0	-	-
	DGPf_2R			-	-			
	DGPf_3F			-	-	0	-	-
	DGPf_3R			-	-			
**96**	DGPf_4F	***P.*** **sp. ICMP 19500**<br>LKBK01000083.1<br>LKBK01000086.1	*P. fluorescens*	-	-	0	-	-
	DGPf_4R			-	-			
	DGPf_5F			0	0	1115	42041	43155
	DGPf_5R			0	0			
	DGPf_6F			-	-	0	-	-
	DGPf_6R			-	-			
	DGPf_7F			2	0	745	11391	10647
	DGPf_7R			1	0			
	DGPf_8F			-	-	0	-	-
	DGPf_8R			-	-			
	DGPf_0F			-	-	0	-	-
	DGPf_0R			-	-			
	DGPf_1F			-	-	0	-	-
	DGPf_1R			-	-			
	DGPf_2F			-	-	0	-	-
	DGPf_2R			-	-			
	DGPf_3F			-	-	0	-	-
	DGPf_3R			-	-			
**97**	DGPf_4F	***P.*** **sp. DSM 29167**<br>JYLO01000003.1<br>JYLO01000004.1	*P. fluorescens*	-	-	0	-	-
	DGPf_4R			-	-			
	DGPf_5F			0	0	1115	316603	317717
	DGPf_5R			0	0			
	DGPf_6F			-	-	0	-	-
	DGPf_6R			-	-			
	DGPf_7F			1	0	745	167321	168065
	DGPf_7R			1	0			
	DGPf_8F			-	-	0	-	-
	DGPf_8R			-	-			
	DGPf_0F			-	-	0	-	-
	DGPf_0R			-	-			
	DGPf_1F			-	-	0	-	-
	DGPf_1R			-	-			
	DGPf_2F			-	-	0	-	-
	DGPf_2R			-	-			
	DGPf_3F			-	-	0	-	-
	DGPf_3R			-	-			
**98**	DGPf_4F	***P. synxantha*** **DSM 18928**<br>JYLJ01000003.1<br>JYLJ01000023.1	*P. fluorescens*	-	-	0	-	-
	DGPf_4R			-	-			
	DGPf_5F			1	0	1115	270203	269089
	DGPf_5R			1	0			
	DGPf_6F			-	-	0	-	-
	DGPf_6R			-	-			
	DGPf_7F			3	0	745	80695	81439
	DGPf_7R			0	0			
	DGPf_8F			-	-	0	-	-
	DGPf_8R			-	-			
	DGPf_0F			-	-	0	-	-
	DGPf_0R			-	-			
	DGPf_1F			-	-	0	-	-
	DGPf_1R			-	-			
	DGPf_2F			-	-	0	-	-
	DGPf_2R			-	-			
	DGPf_3F			-	-	0	-	-
	DGPf_3R			-	-			
**99**	DGPf_4F	***P. fluorescens*** **AU14440**<br>JRXX01000065.1<br>JRXX01000057.1	*P. fluorescens*	-	-	0	-	-
	DGPf_4R			-	-			
	DGPf_5F			0	0	1115	70449	71563
	DGPf_5R			0	0			
	DGPf_6F			-	-	0	-	-
	DGPf_6R			-	-			
	DGPf_7F			1	0	745	1131	387
	DGPf_7R			1	0			
	DGPf_8F			-	-	0	-	-
	DGPf_8R			-	-			
	DGPf_0F			-	-	0	-	-
	DGPf_0R			-	-			
	DGPf_1F			-	-	0	-	-
	DGPf_1R			-	-			
	DGPf_2F			-	-	0	-	-
	DGPf_2R			-	-			
	DGPf_3F			-	-	0	-	-
	DGPf_3R			-	-			
**100**	DGPf_4F	***P.*** **sp. CFT9**<br>ATLM01000005.1<br>ATLM01000017.1	*P. fluorescens*	-	-	0	-	-
	DGPf_4R			-	-			
	DGPf_5F			0	0	1115	81256	82370
	DGPf_5R			0	0			

	DGPf_6F			-	-	0	-	-
	DGPf_6R			-	-			
	DGPf_7F			2	0	745	11364	10620
	DGPf_7R			1	0			
	DGPf_8F			-	-	0	-	-
	DGPf_8R			-	-			
	DGPf_0F			-	-	0	-	-
	DGPf_0R			-	-			
	DGPf_1F			-	-	0	-	-
	DGPf_1R			-	-			
	DGPf_2F			-	-	0	-	-
	DGPf_2R			-	-			
	DGPf_3F			-	-	0	-	-
	DGPf_3R			-	-			
**101**	DGPf_4F	***P.* sp. DSM 29164**<br>JYLN01000005.1<br>JYLN01000010.1	*P. fluorescens*	-	-	0	-	-
	DGPf_4R			-	-			
	DGPf_5F			1	1	1115	335972	337086
	DGPf_5R			0	0			
	DGPf_6F			-	-	0	-	-
	DGPf_6R			-	-			
	DGPf_7F			1	0	745	179104	178360
	DGPf_7R			1	0			
	DGPf_8F			-	-	0	-	-
	DGPf_8R			-	-			
	DGPf_0F			-	-	0	-	-
	DGPf_0R			-	-			
	DGPf_1F			-	-	0	-	-
	DGPf_1R			-	-			
	DGPf_2F			-	-	0	-	-
	DGPf_2R			-	-			
	DGPf_3F			-	-	0	-	-
	DGPf_3R			-	-			
**102**	DGPf_4F	***P. fluorescens* AU6308**<br>JRXZ01000011.1<br>JRXZ01000006.1	*P. fluorescens*	-	-	0	-	-
	DGPf_4R			-	-			
	DGPf_5F			0	0	1115	7421	6307
	DGPf_5R			0	0			
	DGPf_6F			-	-	0	-	-
	DGPf_6R			-	-			
	DGPf_7F			1	0	**736**	100025	99290
	DGPf_7R			2	0			
	DGPf_8F			-	-	0	-	-
	DGPf_8R			-	-			
	DGPf_0F			-	-	0	-	-
	DGPf_0R			-	-			
	DGPf_1F			-	-	0	-	-
	DGPf_1R			-	-			
	DGPf_2F			-	-	0	-	-
	DGPf_2R			-	-			
	DGPf_3F			-	-	0	-	-
	DGPf_3R			-	-			
**103**	DGPf_4F	***P. fluorescens* AU6026**<br>JRXU01000021.1<br>JRXU01000010.1	*P. fluorescens*	-	-	0	-	-
	DGPf_4R			-	-			
	DGPf_5F			0	0	1115	7417	6303
	DGPf_5R			0	0			
	DGPf_6F			-	-	0	-	-
	DGPf_6R			-	-			
	DGPf_7F			1	0	**736**	11643	10908
	DGPf_7R			2	0			
	DGPf_8F			-	-	0	-	-
	DGPf_8R			-	-			
	DGPf_0F			-	-	0	-	-
	DGPf_0R			-	-			
	DGPf_1F			-	-	0	-	-
	DGPf_1R			-	-			
	DGPf_2F			-	-	0	-	-
	DGPf_2R			-	-			
	DGPf_3F			-	-	0	-	-
	DGPf_3R			-	-			
**104**	DGPf_4F	***P. fluorescens* SS101**<br>AHPN01000001.1	*P. fluorescens*	-	-	0	-	-
	DGPf_4R			-	-			
	DGPf_5F			0	0	1115	4430382	4431496
	DGPf_5R			0	0			
	DGPf_6F			-	-	0	-	-
	DGPf_6R			-	-			
	DGPf_7F			1	0	745	3964126	3963382
	DGPf_7R			1	0			
	DGPf_8F			-	-	0	-	-
	DGPf_8R			-	-			
	DGPf_0F			-	-	0	-	-
	DGPf_0R			-	-			
	DGPf_1F			-	-	0	-	-
	DGPf_1R			-	-			
	DGPf_2F			-	-	0	-	-
	DGPf_2R			-	-			
	DGPf_3F			-	-	0	-	-
	DGPf_3R			-	-			
**105**	DGPf_4F	***P.* sp. NBRC 111137**<br>BCBN01000071.1<br>BCBN01000001.1	*P. fluorescens*	-	-	0	-	-
	DGPf_4R			-	-			
	DGPf_5F			1	0	1115	4610	3496
	DGPf_5R			0	0			

	DGPf_6F			-	-	0	-	-
	DGPf_6R			-	-			
	DGPf_7F			1	0	745	43912	44656
	DGPf_7R			1	0			
	DGPf_8F			-	-	0	-	-
	DGPf_8R			-	-			
	DGPf_0F			-	-	0	-	-
	DGPf_0R			-	-			
	DGPf_1F			-	-	0	-	-
	DGPf_1R			-	-			
	DGPf_2F			-	-	0	-	-
	DGPf_2R			-	-			
	DGPf_3F			-	-	0	-	-
	DGPf_3R	***P.* sp. NBRC 111138**		-	-			
**106**	DGPf_4F	BCBO01000120.1	*P. fluorescens*	-	-	0	-	-
	DGPf_4R	BCBO01000145.1		-	-			
	DGPf_5F			0	0	1115	7855	6741
	DGPf_5R			0	0			
	DGPf_6F			-	-	0	-	-
	DGPf_6R			-	-			
	DGPf_7F			1	0	745	5395	4651
	DGPf_7R			1	0			
	DGPf_8F			-	-	0	-	-
	DGPf_8R			-	-			
	DGPf_0F			-	-	0	-	-
	DGPf_0R			-	-			
	DGPf_1F			-	-	0	-	-
	DGPf_1R			-	-			
	DGPf_2F			-	-	0	-	-
	DGPf_2R			-	-			
	DGPf_3F			-	-	0	-	-
	DGPf_3R	***P. fluorescens* 2-79**		-	-			
**107**	DGPf_4F	JXCQ01000114.1	*P. fluorescens*	-	-	0	-	-
	DGPf_4R	JXCQ01000111.1		-	-			
	DGPf_5F			1	0	1115	7226	6112
	DGPf_5R			0	0			
	DGPf_6F			-	-	0	-	-
	DGPf_6R			-	-			
	DGPf_7F			2	0	745	5670	4926
	DGPf_7R			1	0			
	DGPf_8F			-	-	0	-	-
	DGPf_8R			-	-			
	DGPf_0F			-	-	0	-	-
	DGPf_0R			-	-			
	DGPf_1F			-	-	0	-	-
	DGPf_1R			-	-			
	DGPf_2F			-	-	0	-	-
	DGPf_2R			-	-			
	DGPf_3F			-	-	0	-	-
	DGPf_3R			-	-			
**108**	DGPf_4F	***P.* sp. BRG-100**	*P. fluorescens*	-	-	0	-	-
	DGPf_4R	JPRX01000001.1		-	-			
	DGPf_5F			0	0	1115	332562	331448
	DGPf_5R			1	0			
	DGPf_6F			-	-	0	-	-
	DGPf_6R			-	-			
	DGPf_7F			1	0	745	855250	855994
	DGPf_7R			2	0			
	DGPf_8F			-	-	0	-	-
	DGPf_8R			-	-			
	DGPf_0F			-	-	0	-	-
	DGPf_0R			-	-			
	DGPf_1F			-	-	0	-	-
	DGPf_1R			-	-			
	DGPf_2F			-	-	0	-	-
	DGPf_2R			-	-			
	DGPf_3F			-	-	0	-	-
	DGPf_3R	***P.* sp. DSM 28142**		-	-			
**109**	DGPf_4F	JYLP01000015.1	*P. fluorescens*	-	-	0	-	-
	DGPf_4R	JYLP01000023.1		-	-			
	DGPf_5F			0	0	1115	84265	85379
	DGPf_5R			0	0			
	DGPf_6F			-	-	0	-	-
	DGPf_6R			-	-			
	DGPf_7F			1	0	745	14652	15396
	DGPf_7R			1	0			
	DGPf_8F			-	-	0	-	-
	DGPf_8R			-	-			
	DGPf_0F			-	-	0	-	-
	DGPf_0R			-	-			
	DGPf_1F			-	-	0	-	-
	DGPf_1R			-	-			
	DGPf_2F			-	-	0	-	-
	DGPf_2R			-	-			
	DGPf_3F			-	-	0	-	-
	DGPf_3R	***P. synxantha* BG33R**		-	-			
**110**	DGPf_4F	AHPP01000001.1	*P. fluorescens*	-	-	0	-	-
	DGPf_4R	AHPP01000002.1		-	-			
	DGPf_5F			0	0	1115	190306	189192
	DGPf_5R			1	0			

	DGPf_6F			-	-	0	-	-
	DGPf_6R			-	-			
	DGPf_7F			1	0	745	2357669	2356925
	DGPf_7R			2	0			
	DGPf_8F			-	-	0	-	-
	DGPf_8R			-	-			
	DGPf_0F			-	-	0	-	-
	DGPf_0R			-	-			
	DGPf_1F			-	-	0	-	-
	DGPf_1R			-	-			
	DGPf_2F			-	-	0	-	-
	DGPf_2R			-	-			
	DGPf_3F			-	-	0	-	-
	DGPf_3R	***P. marginalis* ICMP 9505**		-	-			
**111**	DGPf_4F	LKGY01000104.1	*P. fluorescens*	-	-	0	-	-
	DGPf_4R	LKGY01000070.1		-	-			
	DGPf_5F			0	0	1115	6767	5653
	DGPf_5R			0	0			
	DGPf_6F			-	-	0	-	-
	DGPf_6R			-	-			
	DGPf_7F			1	0	745	50242	50986
	DGPf_7R			1	0			
	DGPf_8F			-	-	0	-	-
	DGPf_8R			-	-			
	DGPf_0F			-	-	0	-	-
	DGPf_0R			-	-			
	DGPf_1F			-	-	0	-	-
	DGPf_1R			-	-			
	DGPf_2F			1	0	1001	13116	14116
	DGPf_2R			0	0			
	DGPf_3F	***P. tolaasii* 6264**		-	-	0	-	-
	DGPf_3R	AKYY01000110.1		-	-			
**112**	DGPf_4F	AKYY01000146.1	*P. fluorescens*	-	-	0	-	-
	DGPf_4R	AKYY01000208.1		-	-			
	DGPf_5F	AKYY01000049.1		1	0	1115	9262	8148
	DGPf_5R	AKYY01000064.1		0	0			
	DGPf_6F			-	-	0	-	-
	DGPf_6R			-	-			
	DGPf_7F			0	0	?	126-1 AKYY01000208.1	1-629 AKYY01000049.1
	DGPf_7R			1	0			
	DGPf_8F			-	-	0	-	-
	DGPf_8R			-	-			
	DGPf_0F			-	-	0	-	-
	DGPf_0R			-	-			
	DGPf_1F			-	-	0	-	-
	DGPf_1R			-	-			
	DGPf_2F			1	0	1001	12846	13846
	DGPf_2R			0	0			
	DGPf_3F			-	-	0	-	-
	DGPf_3R	***P. tolaasii* PMS117**		-	-			
**113**	DGPf_4F	AJXG01000064.1	*P. fluorescens*	-	-	0	-	-
	DGPf_4R	AJXG01000031.1		-	-			
	DGPf_5F	AJXG01000079.1		1	0	1115	11985	10871
	DGPf_5R	AJXG01000275.1		0	0			
	DGPf_6F			-	-	0	-	-
	DGPf_6R			-	-			
	DGPf_7F			0	0	745	49950	49206
	DGPf_7R			1	0			
	DGPf_8F			-	-	0	-	-
	DGPf_8R			-	-			
	DGPf_0F			-	-	0	-	-
	DGPf_0R			-	-			
	DGPf_1F			-	-	0	-	-
	DGPf_1R			-	-			
	DGPf_2F			-	-	0	-	-
	DGPf_2R			-	-			
	DGPf_3F			-	-	0	-	-
	DGPf_3R	***P. fluorescens* Ps_22**		-	-			
**114**	DGPf_4F	LCYA01000093.1	*P. fluorescens*	-	-	0	-	-
	DGPf_4R	LCYA01000018.1		-	-			
	DGPf_5F			0	0	1115	7528	6414
	DGPf_5R			0	0			
	DGPf_6F			-	-	0	-	-
	DGPf_6R			-	-			
	DGPf_7F			1	0	**800**	32061	32860
	DGPf_7R			1	0			
	DGPf_8F			-	-	0	-	-
	DGPf_8R			-	-			
	DGPf_0F			2	0	612	6063854	6063243
	DGPf_0R			0	0			
	DGPf_1F			0	0	685	86455	87139
	DGPf_1R			0	0			
	DGPf_2F			-	-	0	-	-
	DGPf_2R			-	-			
	DGPf_3F			-	-	0	-	-
	DGPf_3R			-	-			
**115**	DGPf_4F	***P. fluorescens* F113**	*P. corrugata*	-	-	0	-	-
	DGPf_4R	CP003150.1		-	-			
	DGPf_5F			-	-	0	-	-
	DGPf_5R			-	-			

	DGPf_6F			-	-	0	-	-
	DGPf_6R			-	-			
	DGPf_7F			-	-	0	-	-
	DGPf_7R			-	-			
	DGPf_8F			-	-	0	-	-
	DGPf_8R			-	-			
**116**	DGPf_0F	***P. brassicacearum* LBUM300**<br>CP012680.1	*P. corrugata*	1	0	612	6342617	6342006
	DGPf_0R			0	0			
	DGPf_1F			0	0	685	253175	253859
	DGPf_1R			0	0			
	DGPf_2F			-	-	0	-	-
	DGPf_2R			-	-			
	DGPf_3F			-	-	0	-	-
	DGPf_3R			-	-			
	DGPf_4F			-	-	0	-	-
	DGPf_4R			-	-			
	DGPf_5F			-	-	0	-	-
	DGPf_5R			-	-			
	DGPf_6F			-	-	0	-	-
	DGPf_6R			-	-			
	DGPf_7F			-	-	0	-	-
	DGPf_7R			-	-			
	DGPf_8F			-	-	0	-	-
	DGPf_8R			-	-			
**117**	DGPf_0F	***P. brassicacearum* subsp. *brassicacearum* NFM421**<br>CP002585.1	*P. corrugata*	1	0	612	6078839	6078228
	DGPf_0R			0	0			
	DGPf_1F			0	0	685	98958	99642
	DGPf_1R			0	0			
	DGPf_2F			-	-	0	-	-
	DGPf_2R			-	-			
	DGPf_3F			-	-	0	-	-
	DGPf_3R			-	-			
	DGPf_4F			-	-	0	-	-
	DGPf_4R			-	-			
	DGPf_5F			-	-	0	-	-
	DGPf_5R			-	-			
	DGPf_6F			-	-	0	-	-
	DGPf_6R			-	-			
	DGPf_7F			-	-	0	-	-
	DGPf_7R			-	-			
	DGPf_8F			-	-	0	-	-
	DGPf_8R			-	-			
**118**	DGPf_0F	***P. brassicacearum* DF41**<br>CP007410.1	*P. corrugata*	2	0	612	5904325	5903714
	DGPf_0R			0	0			
	DGPf_1F			0	0	685	2541675	2542359
	DGPf_1R			0	0			
	DGPf_2F			-	-	0	-	-
	DGPf_2R			-	-			
	DGPf_3F			-	-	0	-	-
	DGPf_3R			-	-			
	DGPf_4F			-	-	0	-	-
	DGPf_4R			-	-			
	DGPf_5F			-	-	0	-	-
	DGPf_5R			-	-			
	DGPf_6F			-	-	0	-	-
	DGPf_6R			-	-			
	DGPf_7F			-	-	0	-	-
	DGPf_7R			-	-			
	DGPf_8F			-	-	0	-	-
	DGPf_8R			-	-			
**119**	DGPf_0F	***P. fluorescens* Pf29Arp**<br>ANOR01000079.1<br>ANOR01000142.1	*P. corrugata*	1	0	612	30021	29410
	DGPf_0R			0	0			
	DGPf_1F			0	0	685	79333	80017
	DGPf_1R			0	0			
	DGPf_2F			-	-	0	-	-
	DGPf_2R			-	-			
	DGPf_3F			-	-	0	-	-
	DGPf_3R			-	-			
	DGPf_4F			-	-	0	-	-
	DGPf_4R			-	-			
	DGPf_5F			-	-	0	-	-
	DGPf_5R			-	-			
	DGPf_6F			-	-	0	-	-
	DGPf_6R			-	-			
	DGPf_7F			-	-	0	-	-
	DGPf_7R			-	-			
	DGPf_8F			-	-	0	-	-
	DGPf_8R			-	-			
**120**	DGPf_0F	***P. kilonensis* 1855-344**<br>JZXC01000024.1<br>JZXC01000022.1	*P. corrugata*	3	0	612	22275	21664
	DGPf_0R			0	0			
	DGPf_1F			0	0	685	21989	22673
	DGPf_1R			0	0			
	DGPf_2F			-	-	0	-	-
	DGPf_2R			-	-			
	DGPf_3F			-	-	0	-	-
	DGPf_3R			-	-			
	DGPf_4F			-	-	0	-	-
	DGPf_4R			-	-			
	DGPf_5F			-	-	0	-	-
	DGPf_5R			-	-			

	DGPf_6F			-	-	0	-	-
	DGPf_6R			-	-			
	DGPf_7F			-	-	0	-	-
	DGPf_7R			-	-			
	DGPf_8F			-	-	0	-	-
	DGPf_8R			-	-			
	DGPf_0F			3	0	612	296175	295564
	DGPf_0R			0	0			
	DGPf_1F			1	0	685	81770	81086
	DGPf_1R			0	0			
	DGPf_2F			-	-	0	-	-
	DGPf_2R			-	-			
	DGPf_3F			-	-	0	-	-
	DGPf_3R			-	-			
**121**	DGPf_4F	***P.* sp. Root401**<br>LMDO01000064.1<br>LMDO01000012.1	*P. corrugata*	-	-	0	-	-
	DGPf_4R			-	-			
	DGPf_5F			-	-	0	-	-
	DGPf_5R			-	-			
	DGPf_6F			-	-	0	-	-
	DGPf_6R			-	-			
	DGPf_7F			-	-	0	-	-
	DGPf_7R			-	-			
	DGPf_8F			-	-	0	-	-
	DGPf_8R			-	-			
	DGPf_0F			1	0	612	38035	38646
	DGPf_0R			0	0			
	DGPf_1F			0	0	685	3574	2890
	DGPf_1R			0	0			
	DGPf_2F			-	-	0	-	-
	DGPf_2R			-	-			
	DGPf_3F			-	-	0	-	-
	DGPf_3R			-	-			
**122**	DGPf_4F	***P. fluorescens* DSM 8569**<br>JXOE01000032.1<br>JXOE01000013.1	*P. corrugata*	-	-	0	-	-
	DGPf_4R			-	-			
	DGPf_5F			-	-	0	-	-
	DGPf_5R			-	-			
	DGPf_6F			-	-	0	-	-
	DGPf_6R			-	-			
	DGPf_7F			-	-	0	-	-
	DGPf_7R			-	-			
	DGPf_8F			-	-	0	-	-
	DGPf_8R			-	-			
	DGPf_0F			1	0	612	10214	9603
	DGPf_0R			0	0			
	DGPf_1F			0	0	685	260451	261135
	DGPf_1R			0	0			
	DGPf_2F			-	-	0	-	-
	DGPf_2R			-	-			
	DGPf_3F			-	-	0	-	-
	DGPf_3R			-	-			
**123**	DGPf_4F	***P. brassicacearum* PA1G7**<br>JBON01000044.1<br>JBON01000031.1	*P. corrugata*	-	-	0	-	-
	DGPf_4R			-	-			
	DGPf_5F			-	-	0	-	-
	DGPf_5R			-	-			
	DGPf_6F			-	-	0	-	-
	DGPf_6R			-	-			
	DGPf_7F			-	-	0	-	-
	DGPf_7R			-	-			
	DGPf_8F			-	-	0	-	-
	DGPf_8R			-	-			
	DGPf_0F			1	0	612	1605	2216
	DGPf_0R			0	0			
	DGPf_1F			0	0	685	5265	5949
	DGPf_1R			0	0			
	DGPf_2F			-	-	0	-	-
	DGPf_2R			-	-			
	DGPf_3F			-	-	0	-	-
	DGPf_3R			-	-			
**124**	DGPf_4F	***P. fluorescens* Wood1R**<br>CAFF01001085.1<br>CAFF01000503.1	*P. corrugata*	-	-	0	-	-
	DGPf_4R			-	-			
	DGPf_5F			-	-	0	-	-
	DGPf_5R			-	-			
	DGPf_6F			-	-	0	-	-
	DGPf_6R			-	-			
	DGPf_7F			-	-	0	-	-
	DGPf_7R			-	-			
	DGPf_8F			-	-	0	-	-
	DGPf_8R			-	-			
	DGPf_0F			1	0	612	586626	587237
	DGPf_0R			0	0			
	DGPf_1F			0	0	685	24138	23454
	DGPf_1R			0	0			
	DGPf_2F			-	-	0	-	-
	DGPf_2R			-	-			
	DGPf_3F			-	-	0	-	-
	DGPf_3R			-	-			
**125**	DGPf_4F	***P. brassicacearum* PP1 210F**<br>AYJR01000006.1<br>AYJR01000004.1	*P. corrugata*	-	-	0	-	-
	DGPf_4R			-	-			
	DGPf_5F			-	-	0	-	-
	DGPf_5R			-	-			

	DGPf_6F			-	-	0	-	-
	DGPf_6R			-	-			
	DGPf_7F			-	-	0	-	-
	DGPf_7R			-	-			
	DGPf_8F			-	-	0	-	-
	DGPf_8R			-	-			
	DGPf_0F			1	0	612	584330	584941
	DGPf_0R			0	0			
	DGPf_1F			0	0	685	77656	76972
	DGPf_1R			0	0			
	DGPf_2F			-	-	0	-	-
	DGPf_2R			-	-			
	DGPf_3F			-	-	0	-	-
	DGPf_3R	***P. fluorescens* Q8r1-96**		-	-			
**126**	DGPf_4F	AHPO01000005.1	*P. corrugata*	-	-	0	-	-
	DGPf_4R	AHPO01000003.1		-	-			
	DGPf_5F			-	-	0	-	-
	DGPf_5R			-	-			
	DGPf_6F			-	-	0	-	-
	DGPf_6R			-	-			
	DGPf_7F			-	-	0	-	-
	DGPf_7R			-	-			
	DGPf_8F			-	-	0	-	-
	DGPf_8R			-	-			
	DGPf_0F			1	0	612	5217	5828
	DGPf_0R			0	0			
	DGPf_1F			0	0	685	482193	482877
	DGPf_1R			0	0			
	DGPf_2F			-	-	0	-	-
	DGPf_2R			-	-			
	DGPf_3F			-	-	0	-	-
	DGPf_3R	***P.* sp. URIL14HWK12:I7**		-	-			
**127**	DGPf_4F	AZVP01000049.1	*P. corrugata*	-	-	0	-	-
	DGPf_4R	AZVP01000005.1		-	-			
	DGPf_5F			-	-	0	-	-
	DGPf_5R			-	-			
	DGPf_6F			-	-	0	-	-
	DGPf_6R			-	-			
	DGPf_7F			-	-	0	-	-
	DGPf_7R			-	-			
	DGPf_8F			-	-	0	-	-
	DGPf_8R			-	-			
	DGPf_0F			2	0	612	27363	26752
	DGPf_0R			0	0			
	DGPf_1F			0	0	685	25388	24704
	DGPf_1R			0	0			
	DGPf_2F			-	-	0	-	-
	DGPf_2R			-	-			
	DGPf_3F			-	-	0	-	-
	DGPf_3R	***P.* sp. CFII68**		-	-			
**128**	DGPf_4F	ATLN01000122.1	*P. corrugata*	-	-	0	-	-
	DGPf_4R	ATLN01000018.1		-	-			
	DGPf_5F			-	-	0	-	-
	DGPf_5R			-	-			
	DGPf_6F			-	-	0	-	-
	DGPf_6R			-	-			
	DGPf_7F			-	-	0	-	-
	DGPf_7R			-	-			
	DGPf_8F			-	-	0	-	-
	DGPf_8R			-	-			
	DGPf_0F			2	0	612	23698	23087
	DGPf_0R			0	0			
	DGPf_1F			0	0	685	16668	15984
	DGPf_1R			0	0			
	DGPf_2F			-	-	0	-	-
	DGPf_2R			-	-			
	DGPf_3F			-	-	0	-	-
	DGPf_3R	***P. fluorescens* et76**		-	-			
**129**	DGPf_4F	LNAB01000215.1	*P. corrugata*	-	-	0	-	-
	DGPf_4R	LNAB01000033.1		-	-			
	DGPf_5F			-	-	0	-	-
	DGPf_5R			-	-			
	DGPf_6F			-	-	0	-	-
	DGPf_6R			-	-			
	DGPf_7F			-	-	0	-	-
	DGPf_7R			-	-			
	DGPf_8F			-	-	0	-	-
	DGPf_8R			-	-			
	DGPf_0F			2	0	612	11856	11245
	DGPf_0R			0	0			
	DGPf_1F			0	0	685	24385	25069
	DGPf_1R			0	0			
	DGPf_2F			-	-	0	-	-
	DGPf_2R			-	-			
	DGPf_3F			-	-	0	-	-
	DGPf_3R	***P. fluorescens* S12**		-	-			
**130**	DGPf_4F	AVFN01000083.1	*P. corrugata*	-	-	0	-	-
	DGPf_4R	AVFN01000051.1		-	-			
	DGPf_5F			-	-	0	-	-
	DGPf_5R			-	-			

	DGPf_6F			-	-	0	-	-
	DGPf_6R			-	-			
	DGPf_7F			-	-	0	-	-
	DGPf_7R			-	-			
	DGPf_8F			-	-	0	-	-
	DGPf_8R			-	-			
	DGPf_0F			1	0	612	89091	89702
	DGPf_0R			0	0			
	DGPf_1F			0	0	685	29668	28984
	DGPf_1R			0	0			
	DGPf_2F			-	-	0	-	-
	DGPf_2R			-	-			
	DGPf_3F			-	-	0	-	-
	DGPf_3R	***P. brassicacearum* 51MFCvi2.1**		-	-			
131	DGPf_4F	AZOC01000034.1	*P. corrugata*	-	-	0	-	-
	DGPf_4R	AZOC01000011.1		-	-			
	DGPf_5F			-	-	0	-	-
	DGPf_5R			-	-			
	DGPf_6F			-	-	0	-	-
	DGPf_6R			-	-			
	DGPf_7F			-	-	0	-	-
	DGPf_7R			-	-			
	DGPf_8F			-	-	0	-	-
	DGPf_8R			-	-			
	DGPf_0F			1	0	612	10058	9447
	DGPf_0R			0	0			
	DGPf_1F			0	0	685	60941	61625
	DGPf_1R			0	0			
	DGPf_2F			-	-	0	-	-
	DGPf_2R			-	-			
	DGPf_3F			-	-	0	-	-
	DGPf_3R	***P. brassicacearum* LZ-4**		-	-			
132	DGPf_4F	JNCR01000033.1	*P. corrugata*	-	-	0	-	-
	DGPf_4R	JNCR01000034.1		-	-			
	DGPf_5F			-	-	0	-	-
	DGPf_5R			-	-			
	DGPf_6F			-	-	0	-	-
	DGPf_6R			-	-			
	DGPf_7F			-	-	0	-	-
	DGPf_7R			-	-			
	DGPf_8F			-	-	0	-	-
	DGPf_8R			-	-			
	DGPf_0F			1	0	612	12070	12681
	DGPf_0R			**1**	0			
	DGPf_1F			1	0	685	26879	27563
	DGPf_1R			0	0			
	DGPf_2F			-	-	0	-	-
	DGPf_2R			-	-			
	DGPf_3F			-	-	0	-	-
	DGPf_3R	***P. corrugata* TEIC1148**		-	-			
133	DGPf_4F	LIHH01000221.1	*P. corrugata*	-	-	0	-	-
	DGPf_4R	LIHH01000054.1		-	-			
	DGPf_5F			-	-	0	-	-
	DGPf_5R			-	-			
	DGPf_6F			-	-	0	-	-
	DGPf_6R			-	-			
	DGPf_7F			-	-	0	-	-
	DGPf_7R			-	-			
	DGPf_8F			-	-	0	-	-
	DGPf_8R			-	-			
	DGPf_0F			1	0	612	24015	24626
	DGPf_0R			**1**	0			
	DGPf_1F			1	0	685	185966	186650
	DGPf_1R			0	0			
	DGPf_2F			-	-	0	-	-
	DGPf_2R			-	-			
	DGPf_3F			-	-	0	-	-
	DGPf_3R	***P. corrugata* NCPPB2445**		-	-			
134	DGPf_4F	LIGR01000005.1	*P. corrugata*	-	-	0	-	-
	DGPf_4R	LIGR01000036.1		-	-			
	DGPf_5F			-	-	0	-	-
	DGPf_5R			-	-			
	DGPf_6F			-	-	0	-	-
	DGPf_6R			-	-			
	DGPf_7F			-	-	0	-	-
	DGPf_7R			-	-			
	DGPf_8F			-	-	0	-	-
	DGPf_8R			-	-			
	DGPf_0F			1	0	612	22027	21416
	DGPf_0R			**1**	0			
	DGPf_1F			1	0	685	1626	942
	DGPf_1R			0	0			
	DGPf_2F			-	-	0	-	-
	DGPf_2R			-	-			
	DGPf_3F			-	-	0	-	-
	DGPf_3R	***P. corrugata* CFBP5403**		-	-			
135	DGPf_4F	LIGO01000009.1	*P. corrugata*	-	-	0	-	-
	DGPf_4R	LIGO01000138.1		-	-			
	DGPf_5F			-	-	0	-	-
	DGPf_5R			-	-			

	DGPf_6F			-	-	0	-	-
	DGPf_6R			-	-			
	DGPf_7F			-	-	0	-	-
	DGPf_7R			-	-			
	DGPf_8F			-	-	0	-	-
	DGPf_8R			-	-			
136	DGPf_0F	***P. corrugata* CFBP 5454**<br>ATKI01000044.1<br>ATKI01000047.1	*P. corrugata*	1	0	612	55879	56490
	DGPf_0R			1	0			
	DGPf_1F			1	0	685	29290	28606
	DGPf_1R			0	0			
	DGPf_2F			-	-	0	-	-
	DGPf_2R			-	-			
	DGPf_3F			-	-	0	-	-
	DGPf_3R			-	-			
	DGPf_4F			-	-	0	-	-
	DGPf_4R			-	-			
	DGPf_5F			-	-	0	-	-
	DGPf_5R			-	-			
	DGPf_6F			-	-	0	-	-
	DGPf_6R			-	-			
	DGPf_7F			-	-	0	-	-
	DGPf_7R			-	-			
	DGPf_8F			-	-	0	-	-
	DGPf_8R			-	-			
137	DGPf_0F	***P. fluorescens* NT0133**<br>JYHW01000065.1<br>JYHW01000044.1	*P. corrugata*	2	0	612	17150	16539
	DGPf_0R			0	0			
	DGPf_1F			0	0	685	216599	215915
	DGPf_1R			1	0			
	DGPf_2F			-	-	0	-	-
	DGPf_2R			-	-			
	DGPf_3F			-	-	0	-	-
	DGPf_3R			-	-			
	DGPf_4F			-	-	0	-	-
	DGPf_4R			-	-			
	DGPf_5F			-	-	0	-	-
	DGPf_5R			-	-			
	DGPf_6F			-	-	0	-	-
	DGPf_6R			-	-			
	DGPf_7F			-	-	0	-	-
	DGPf_7R			-	-			
	DGPf_8F			-	-	0	-	-
	DGPf_8R			-	-			
138	DGPf_0F	***P. frederiksbergensis* SI8**<br>JQGJ02000025.1<br>JQGJ02000005.1	*P. corrugata*	2	0	612	30658	30047
	DGPf_0R			1	0			
	DGPf_1F			0	0	685	293840	294524
	DGPf_1R			1	0			
	DGPf_2F			-	-	0	-	-
	DGPf_2R			-	-			
	DGPf_3F			-	-	0	-	-
	DGPf_3R			-	-			
	DGPf_4F			-	-	0	-	-
	DGPf_4R			-	-			
	DGPf_5F			-	-	0	-	-
	DGPf_5R			-	-			
	DGPf_6F			-	-	0	-	-
	DGPf_6R			-	-			
	DGPf_7F			-	-	0	-	-
	DGPf_7R			-	-			
	DGPf_8F			-	-	0	-	-
	DGPf_8R			-	-			
139	DGPf_0F	***P. mediterranea* TEIC1022**<br>LJWU01000032.1<br>LJWU01000039.1	*P. corrugata*	1	0	612	19419	18808
	DGPf_0R			1	0			
	DGPf_1F			0	0	685	63029	63713
	DGPf_1R			0	0			
	DGPf_2F			-	-	0	-	-
	DGPf_2R			-	-			
	DGPf_3F			-	-	0	-	-
	DGPf_3R			-	-			
	DGPf_4F			-	-	0	-	-
	DGPf_4R			-	-			
	DGPf_5F			-	-	0	-	-
	DGPf_5R			-	-			
	DGPf_6F			-	-	0	-	-
	DGPf_6R			-	-			
	DGPf_7F			-	-	0	-	-
	DGPf_7R			-	-			
	DGPf_8F			-	-	0	-	-
	DGPf_8R			-	-			
140	DGPf_0F	***P.* sp. SHC52**<br>CBLV010000106.1<br>CBLV010000121.1	*P. corrugata*	1	0	612	12745	12134
	DGPf_0R			1	0			
	DGPf_1F			1	0	685	9485	8801
	DGPf_1R			0	0			
	DGPf_2F			-	-	0	-	-
	DGPf_2R			-	-			
	DGPf_3F			-	-	0	-	-
	DGPf_3R			-	-			
	DGPf_4F			-	-	0	-	-
	DGPf_4R			-	-			
	DGPf_5F			-	-	0	-	-
	DGPf_5R			-	-			

	DGPf_6F			-	-	0	-	-
	DGPf_6R			-	-			
	DGPf_7F			-	-	0	-	-
	DGPf_7R			-	-			
	DGPf_8F			-	-	0	-	-
	DGPf_8R			-	-			
	DGPf_0F			1	0	612	654979	654368
	DGPf_0R			1	0			
	DGPf_1F			0	0	685	689052	689736
	DGPf_1R			0	0			
	DGPf_2F			-	-	0	-	-
	DGPf_2R			-	-			
	DGPf_3F			-	-	0	-	-
	DGPf_3R	***P. mediterranea*** **CFBP 5447**		-	-			
**141**	DGPf_4F	AUPB01000038.1	*P. corrugata*	-	-	0	-	-
	DGPf_4R	AUPB01000033.1		-	-			
	DGPf_5F			-	-	0	-	-
	DGPf_5R			-	-			
	DGPf_6F			-	-	0	-	-
	DGPf_6R			-	-			
	DGPf_7F			-	-	0	-	-
	DGPf_7R			-	-			
	DGPf_8F			-	-	0	-	-
	DGPf_8R			-	-			
	DGPf_0F			1	0	612	19886	19275
	DGPf_0R			1	0			
	DGPf_1F			0	0	685	69788	69104
	DGPf_1R			0	0			
	DGPf_2F			-	-	0	-	-
	DGPf_2R			-	-			
	DGPf_3F			-	-	0	-	-
	DGPf_3R	***P. mediterranea*** **CFBP5444**		-	-			
**142**	DGPf_4F	LIHG01000034.1	*P. corrugata*	-	-	0	-	-
	DGPf_4R	LIHG01000018.1		-	-			
	DGPf_5F			-	-	0	-	-
	DGPf_5R			-	-			
	DGPf_6F			-	-	0	-	-
	DGPf_6R			-	-			
	DGPf_7F			-	-	0	-	-
	DGPf_7R			-	-			
	DGPf_8F			-	-	0	-	-
	DGPf_8R			-	-			
	DGPf_0F			1	0	612	20541	19930
	DGPf_0R			1	0			
	DGPf_1F			0	0	685	49763	50447
	DGPf_1R			0	0			
	DGPf_2F			-	-	0	-	-
	DGPf_2R			-	-			
	DGPf_3F			-	-	0	-	-
	DGPf_3R	***P. mediterranea*** **CFBP5404**		-	-			
**143**	DGPf_4F	LIGZ01000033.1	*P. corrugata*	-	-	0	-	-
	DGPf_4R	LIGZ01000020.1		-	-			
	DGPf_5F			-	-	0	-	-
	DGPf_5R			-	-			
	DGPf_6F			-	-	0	-	-
	DGPf_6R			-	-			
	DGPf_7F			-	-	0	-	-
	DGPf_7R			-	-			
	DGPf_8F			-	-	0	-	-
	DGPf_8R			-	-			
	DGPf_0F			1	0	612	57490	58101
	DGPf_0R			1	0			
	DGPf_1F			0	0	685	69766	69082
	DGPf_1R			0	0			
	DGPf_2F			-	-	0	-	-
	DGPf_2R			-	-			
	DGPf_3F			-	-	0	-	-
	DGPf_3R	***P. mediterranea*** **TEIC1105**		-	-			
**144**	DGPf_4F	LIGN01000004.1	*P. corrugata*	-	-	0	-	-
	DGPf_4R	LIGN01000007.1		-	-			
	DGPf_5F			-	-	0	-	-
	DGPf_5R			-	-			
	DGPf_6F			-	-	0	-	-
	DGPf_6R			-	-			
	DGPf_7F			-	-	0	-	-
	DGPf_7R			-	-			
	DGPf_8F			-	-	0	-	-
	DGPf_8R			-	-			
	DGPf_0F			2	0	612	699266	699877
	DGPf_0R			0	0			
	DGPf_1F			0	0	685	3971453	3972137
	DGPf_1R			0	0			
	DGPf_2F			-	-	0	-	-
	DGPf_2R			-	-			
	DGPf_3F			-	-	0	-	-
	DGPf_3R	***P. fluorescens*** **Q2-87**		-	-			
**145**	DGPf_4F	AGBM01000001.1	*P. corrugata*	-	-	0	-	-
	DGPf_4R	AGBM01000001.1		-	-			
	DGPf_5F			-	-	0	-	-
	DGPf_5R			-	-			

	DGPf_6F			-	-	0	-	-
	DGPf_6R			-	-			
	DGPf_7F			-	-	0	-	-
	DGPf_7R			-	-			
	DGPf_8F			-	-	0	-	-
	DGPf_8R			-	-			
146	DGPf_0F	***P.* sp. Q12-87** LHVI01000033.1 LHVI01000014.1	*P. corrugata*	2	0	612	85046	84435
	DGPf_0R			0	0			
	DGPf_1F			0	0	685	130584	129900
	DGPf_1R			0	0			
	DGPf_2F			-	-	0	-	-
	DGPf_2R			-	-			
	DGPf_3F			-	-	0	-	-
	DGPf_3R			-	-			
	DGPf_4F			-	-	0	-	-
	DGPf_4R			-	-			
	DGPf_5F			-	-	0	-	-
	DGPf_5R			-	-			
	DGPf_6F			-	-	0	-	-
	DGPf_6R			-	-			
	DGPf_7F			-	-	0	-	-
	DGPf_7R			-	-			
	DGPf_8F			-	-	0	-	-
	DGPf_8R			-	-			
147	DGPf_0F	***P. fluorescens* Pf0-1** CP000094.2	*P. koreensis*	1	0	612	5640032	5639421
	DGPf_0R			0	0			
	DGPf_1F			-	-	0	-	-
	DGPf_1R			-	-			
	DGPf_2F			1	0	1001	2426662	2425662
	DGPf_2R			0	0			
	DGPf_3F			-	-	0	-	-
	DGPf_3R			-	-			
	DGPf_4F			-	-	0	-	-
	DGPf_4R			-	-			
	DGPf_5F			-	-	0	-	-
	DGPf_5R			-	-			
	DGPf_6F			-	-	0	-	-
	DGPf_6R			-	-			
	DGPf_7F			-	-	0	-	-
	DGPf_7R			-	-			
	DGPf_8F			-	-	0	-	-
	DGPf_8R			-	-			
148	DGPf_0F	***P. fluorescens* SF4c** JTGH01000020.1 JTGH01000016.1	*P. koreensis*	1	0	612	245863	245252
	DGPf_0R			0	0			
	DGPf_1F			-	-	0	-	-
	DGPf_1R			-	-			
	DGPf_2F			2	0	1001	119854	120854
	DGPf_2R			0	0			
	DGPf_3F			-	-	0	-	-
	DGPf_3R			-	-			
	DGPf_4F			-	-	0	-	-
	DGPf_4R			-	-			
	DGPf_5F			-	-	0	-	-
	DGPf_5R			-	-			
	DGPf_6F			-	-	0	-	-
	DGPf_6R			-	-			
	DGPf_7F			-	-	0	-	-
	DGPf_7R			-	-			
	DGPf_8F			-	-	0	-	-
	DGPf_8R			-	-			
149	DGPf_0F	***P.* sp. GM25** AKJQ01000039.1 AKJQ01000048.1	*P. koreensis*	1	0	612	3571	2960
	DGPf_0R			0	0			
	DGPf_1F			-	-	0	-	-
	DGPf_1R			-	-			
	DGPf_2F			1	0	1001	112079	113079
	DGPf_2R			0	0			
	DGPf_3F			-	-	0	-	-
	DGPf_3R			-	-			
	DGPf_4F			-	-	0	-	-
	DGPf_4R			-	-			
	DGPf_5F			-	-	0	-	-
	DGPf_5R			-	-			
	DGPf_6F			-	-	0	-	-
	DGPf_6R			-	-			
	DGPf_7F			-	-	0	-	-
	DGPf_7R			-	-			
	DGPf_8F			-	-	0	-	-
	DGPf_8R			-	-			
150	DGPf_0F	***P.* sp. Leaf434** LMQZ01000011.1 LMQZ01000009.1	*P. koreensis*	1	0	612	232327	231716
	DGPf_0R			1	0			
	DGPf_1F			-	-	0	-	-
	DGPf_1R			-	-			
	DGPf_2F			1	0	1001	300800	299800
	DGPf_2R			0	0			
	DGPf_3F			-	-	0	-	-
	DGPf_3R			-	-			
	DGPf_4F			-	-	0	-	-
	DGPf_4R			-	-			
	DGPf_5F			-	-	0	-	-
	DGPf_5R			-	-			

	DGPf_6F			-	-	0	-	-
	DGPf_6R			-	-			
	DGPf_7F			-	-	0	-	-
	DGPf_7R			-	-			
	DGPf_8F			-	-	0	-	-
	DGPf_8R			-	-			
	DGPf_0F			1	0	612	234021	233410
	DGPf_0R			0	0			
	DGPf_1F			-	-	0	-	-
	DGPf_1R			-	-			
	DGPf_2F			1	0	1001	217543	218543
	DGPf_2R			0	0			
	DGPf_3F			-	-	0	-	-
	DGPf_3R	***P. fluorescens* SF39a**		-	-			
**151**	DGPf_4F	JTGG01000013.1	*P. koreensis*	-	-	0	-	-
	DGPf_4R	JTGG01000022.1		-	-			
	DGPf_5F			-	-	0	-	-
	DGPf_5R			-	-			
	DGPf_6F			-	-	0	-	-
	DGPf_6R			-	-			
	DGPf_7F			-	-	0	-	-
	DGPf_7R			-	-			
	DGPf_8F			-	-	0	-	-
	DGPf_8R			-	-			
	DGPf_0F			2	0	612	96656	96045
	DGPf_0R			0	0			
	DGPf_1F			-	-	0	-	-
	DGPf_1R			-	-			
	DGPf_2F			1	0	1001	35962	34962
	DGPf_2R			0	0			
	DGPf_3F			-	-	0	-	-
	DGPf_3R	***P. fluorescens* AU11114**		-	-			
**152**	DGPf_4F	LCZE01000041.1	*P. koreensis*	-	-	0	-	-
	DGPf_4R	LCZE01000020.1		-	-			
	DGPf_5F			-	-	0	-	-
	DGPf_5R			-	-			
	DGPf_6F			-	-	0	-	-
	DGPf_6R			-	-			
	DGPf_7F			-	-	0	-	-
	DGPf_7R			-	-			
	DGPf_8F			-	-	0	-	-
	DGPf_8R			-	-			
	DGPf_0F			1	0	612	541651	541040
	DGPf_0R			0	0			
	DGPf_1F			-	-	0	-	-
	DGPf_1R			-	-			
	DGPf_2F			1	0	1001	594743	595743
	DGPf_2R			0	0			
	DGPf_3F			-	-	0	-	-
	DGPf_3R	***P. moraviensis* R28-S**		-	-			
**153**	DGPf_4F	AYMZ01000010.1	*P. koreensis*	-	-	0	-	-
	DGPf_4R	AYMZ01000006.1		-	-			
	DGPf_5F			-	-	0	-	-
	DGPf_5R			-	-			
	DGPf_6F			-	-	0	-	-
	DGPf_6R			-	-			
	DGPf_7F			-	-	0	-	-
	DGPf_7R			-	-			
	DGPf_8F			-	-	0	-	-
	DGPf_8R			-	-			
	DGPf_0F			2	0	612	5516387	5515776
	DGPf_0R			0	0			
	DGPf_1F			-	-	0	-	-
	DGPf_1R			-	-			
	DGPf_2F			1	0	1001	2370646	2369646
	DGPf_2R			0	0			
	DGPf_3F			-	-	0	-	-
	DGPf_3R			-	-			
**154**	DGPf_4F	***P. fluorescens* SRM1**	*P. koreensis*	-	-	0	-	-
	DGPf_4R	CDMF01000001.1		-	-			
	DGPf_5F			-	-	0	-	-
	DGPf_5R			-	-			
	DGPf_6F			-	-	0	-	-
	DGPf_6R			-	-			
	DGPf_7F			-	-	0	-	-
	DGPf_7R			-	-			
	DGPf_8F			-	-	0	-	-
	DGPf_8R			-	-			
	DGPf_0F			1	0	612	178901	178290
	DGPf_0R			0	0			
	DGPf_1F			-	-	0	-	-
	DGPf_1R			-	-			
	DGPf_2F			1	0	1001	353942	352942
	DGPf_2R			0	0			
	DGPf_3F			-	-	0	-	-
	DGPf_3R	***P.* sp. URIL14HWK12:I6**		-	-			
**155**	DGPf_4F	AZVL01000012.1	*P. koreensis*	-	-	0	-	-
	DGPf_4R	AZVL01000004.1		-	-			
	DGPf_5F			-	-	0	-	-
	DGPf_5R			-	-			

	DGPf_6F			-	-	0	-	-
	DGPf_6R			-	-			
	DGPf_7F			-	-	0	-	-
	DGPf_7R			-	-			
	DGPf_8F			-	-	0	-	-
	DGPf_8R			-	-			
**156**	DGPf_0F	***P.* sp. R62** AHZM01000351.1 AHZM01000744.1	*P. koreensis*	1	0	612	7404	8015
	DGPf_0R			0	0			
	DGPf_1F			-	-	0	-	-
	DGPf_1R			-	-			
	DGPf_2F			1	0	1001	2141	3141
	DGPf_2R			0	0			
	DGPf_3F			-	-	0	-	-
	DGPf_3R			-	-			
	DGPf_4F			-	-	0	-	-
	DGPf_4R			-	-			
	DGPf_5F			-	-	0	-	-
	DGPf_5R			-	-			
	DGPf_6F			-	-	0	-	-
	DGPf_6R			-	-			
	DGPf_7F			-	-	0	-	-
	DGPf_7R			-	-			
	DGPf_8F			-	-	0	-	-
	DGPf_8R			-	-			
**157**	DGPf_0F	***P. fluorescens* MEP34** JXQY01000038.1 JXQY01000023.1	*P. koreensis*	1	0	612	235135	234524
	DGPf_0R			0	0			
	DGPf_1F			-	-	0	-	-
	DGPf_1R			-	-			
	DGPf_2F			1	0	1001	407703	406703
	DGPf_2R			0	0			
	DGPf_3F			-	-	0	-	-
	DGPf_3R			-	-			
	DGPf_4F			-	-	0	-	-
	DGPf_4R			-	-			
	DGPf_5F			-	-	0	-	-
	DGPf_5R			-	-			
	DGPf_6F			-	-	0	-	-
	DGPf_6R			-	-			
	DGPf_7F			-	-	0	-	-
	DGPf_7R			-	-			
	DGPf_8F			-	-	0	-	-
	DGPf_8R			-	-			
**158**	DGPf_0F	***P.* sp. PTA1** JQJQ01000009.1 JQJQ01000022.1	*P. koreensis*	1	0	612	65185	65796
	DGPf_0R			0	0			
	DGPf_1F			-	-	0	-	-
	DGPf_1R			-	-			
	DGPf_2F			1	0	1001	18391	19391
	DGPf_2R			0	0			
	DGPf_3F			-	-	0	-	-
	DGPf_3R			-	-			
	DGPf_4F			-	-	0	-	-
	DGPf_4R			-	-			
	DGPf_5F			-	-	0	-	-
	DGPf_5R			-	-			
	DGPf_6F			-	-	0	-	-
	DGPf_6R			-	-			
	DGPf_7F			-	-	0	-	-
	DGPf_7R			-	-			
	DGPf_8F			-	-	0	-	-
	DGPf_8R			-	-			
**159**	DGPf_0F	***P.* sp. W15Feb9B** JTKF01000007.1 JTKF01000045.1	*P. koreensis*	1	0	612	4884	5495
	DGPf_0R			0	0			
	DGPf_1F			-	-	0	-	-
	DGPf_1R			-	-			
	DGPf_2F			1	0	1001	88383	89383
	DGPf_2R			0	0			
	DGPf_3F			-	-	0	-	-
	DGPf_3R			-	-			
	DGPf_4F			-	-	0	-	-
	DGPf_4R			-	-			
	DGPf_5F			-	-	0	-	-
	DGPf_5R			-	-			
	DGPf_6F			-	-	0	-	-
	DGPf_6R			-	-			
	DGPf_7F			-	-	0	-	-
	DGPf_7R			-	-			
	DGPf_8F			-	-	0	-	-
	DGPf_8R			-	-			
**160**	DGPf_0F	***P.* sp. GM16** AKJV01000012.1 AKJV01000098.1	*P. koreensis*	1	0	612	26756	26145
	DGPf_0R			0	0			
	DGPf_1F			-	-	0	-	-
	DGPf_1R			-	-			
	DGPf_2F			1	0	1001	8946	9946
	DGPf_2R			0	0			
	DGPf_3F			-	-	0	-	-
	DGPf_3R			-	-			
	DGPf_4F			-	-	0	-	-
	DGPf_4R			-	-			
	DGPf_5F			-	-	0	-	-
	DGPf_5R			-	-			

	DGPf_6F			-	-	0	-	-
	DGPf_6R			-	-			
	DGPf_7F			-	-	0	-	-
	DGPf_7R			-	-			
	DGPf_8F			-	-	0	-	-
	DGPf_8R			-	-			
	DGPf_0F			1	0	612	31920	32531
	DGPf_0R			0	0			
	DGPf_1F			-	-	0	-	-
	DGPf_1R			-	-			
	DGPf_2F			1	0	1001	4238	3238
	DGPf_2R			0	0			
	DGPf_3F			-	-	0	-	-
	DGPf_3R			-	-			
161	DGPf_4F	***P.* sp. GM24**<br>AKJR01000020.1<br>AKJR01000066.1	*P. koreensis*	-	-	0	-	-
	DGPf_4R			-	-			
	DGPf_5F			-	-	0	-	-
	DGPf_5R			-	-			
	DGPf_6F			-	-	0	-	-
	DGPf_6R			-	-			
	DGPf_7F			-	-	0	-	-
	DGPf_7R			-	-			
	DGPf_8F			-	-	0	-	-
	DGPf_8R			-	-			
	DGPf_0F			1	0	612	92114	92725
	DGPf_0R			0	0			
	DGPf_1F			-	-	0	-	-
	DGPf_1R			-	-			
	DGPf_2F			1	0	1001	155953	156953
	DGPf_2R			0	0			
	DGPf_3F			-	-	0	-	-
	DGPf_3R			-	-			
162	DGPf_4F	***P.* sp. RIT288**<br>JFYN01000044.1<br>JFYN01000042.1	*P. koreensis*	-	-	0	-	-
	DGPf_4R			-	-			
	DGPf_5F			-	-	0	-	-
	DGPf_5R			-	-			
	DGPf_6F			-	-	0	-	-
	DGPf_6R			-	-			
	DGPf_7F			-	-	0	-	-
	DGPf_7R			-	-			
	DGPf_8F			-	-	0	-	-
	DGPf_8R			-	-			
	DGPf_0F			1	0	612	23230	23841
	DGPf_0R			0	0			
	DGPf_1F			-	-	0	-	-
	DGPf_1R			-	-			
	DGPf_2F			1	0	1001	30653	29653
	DGPf_2R			0	0			
	DGPf_3F			-	-	0	-	-
	DGPf_3R			-	-			
163	DGPf_4F	***P. fluorescens* H16**<br>JSAL01000014.1<br>JSAL01000026.1	*P. koreensis*	-	-	0	-	-
	DGPf_4R			-	-			
	DGPf_5F			-	-	0	-	-
	DGPf_5R			-	-			
	DGPf_6F			-	-	0	-	-
	DGPf_6R			-	-			
	DGPf_7F			-	-	0	-	-
	DGPf_7R			-	-			
	DGPf_8F			-	-	0	-	-
	DGPf_8R			-	-			
	DGPf_0F			1	0	612	90208	90819
	DGPf_0R			0	0			
	DGPf_1F			-	-	0	-	-
	DGPf_1R			-	-			
	DGPf_2F			1	0	1001	221967	220967
	DGPf_2R			0	0			
	DGPf_3F			-	-	0	-	-
	DGPf_3R			-	-			
164	DGPf_4F	***P.* sp. GM30**<br>AKJP02000006.1<br>AKJP02000030.1	*P. koreensis*	-	-	0	-	-
	DGPf_4R			-	-			
	DGPf_5F			-	-	0	-	-
	DGPf_5R			-	-			
	DGPf_6F			-	-	0	-	-
	DGPf_6R			-	-			
	DGPf_7F			-	-	0	-	-
	DGPf_7R			-	-			
	DGPf_8F			-	-	0	-	-
	DGPf_8R			-	-			
	DGPf_0F			1	0	612	87861	88472
	DGPf_0R			0	0			
	DGPf_1F			-	-	0	-	-
	DGPf_1R			-	-			
	DGPf_2F			1	0	1001	221586	222586
	DGPf_2R			0	0			
	DGPf_3F			-	-	0	-	-
	DGPf_3R			-	-			
165	DGPf_4F	***P.* sp. RIT-PI-o**<br>LHPA01000024.1<br>LHPA01000021.1	*P. koreensis*	-	-	0	-	-
	DGPf_4R			-	-			
	DGPf_5F			-	-	0	-	-
	DGPf_5R			-	-			

	DGPf_6F			-	-	0	-	-
	DGPf_6R			-	-			
	DGPf_7F			-	-	0	-	-
	DGPf_7R			-	-			
	DGPf_8F			-	-	0	-	-
	DGPf_8R			-	-			
	DGPf_0F			2	0	612	18365	17754
	DGPf_0R			0	0			
	DGPf_1F			-	-	0	-	-
	DGPf_1R			-	-			
	DGPf_2F			1	0	1001	4293	5293
	DGPf_2R			0	0			
	DGPf_3F			-	-	0	-	-
	DGPf_3R	***P.* sp. GM80**		-	-			
166	DGPf_4F	AKJD01000080.1	*P. koreensis*	-	-	0	-	-
	DGPf_4R	AKJD01000265.1		-	-			
	DGPf_5F			-	-	0	-	-
	DGPf_5R			-	-			
	DGPf_6F			-	-	0	-	-
	DGPf_6R			-	-			
	DGPf_7F			-	-	0	-	-
	DGPf_7R			-	-			
	DGPf_8F			-	-	0	-	-
	DGPf_8R			-	-			
	DGPf_0F			1	0	612	264065	263454
	DGPf_0R			0	0			
	DGPf_1F			-	-	0	-	-
	DGPf_1R			-	-			
	DGPf_2F			1	0	1001	162454	161454
	DGPf_2R			0	0			
	DGPf_3F			-	-	0	-	-
	DGPf_3R	***P. fluorescens* AU5633**		-	-			
167	DGPf_4F	LCZD01000027.1	*P. koreensis*	-	-	0	-	-
	DGPf_4R	LCZD01000014.1		-	-			
	DGPf_5F			-	-	0	-	-
	DGPf_5R			-	-			
	DGPf_6F			-	-	0	-	-
	DGPf_6R			-	-			
	DGPf_7F			-	-	0	-	-
	DGPf_7R			-	-			
	DGPf_8F			-	-	0	-	-
	DGPf_8R			-	-			
	DGPf_0F			1	0	612	196362	195751
	DGPf_0R			1	0			
	DGPf_1F			-	-	0	-	-
	DGPf_1R			-	-			
	DGPf_2F			1	0	1001	73865	72865
	DGPf_2R			0	0			
	DGPf_3F			-	-	0	-	-
	DGPf_3R	***P. chlororaphis* EA105**		-	-			
168	DGPf_4F	JSFK01000006.1	*P. koreensis*	-	-	0	-	-
	DGPf_4R	JSFK01000022.1		-	-			
	DGPf_5F			-	-	0	-	-
	DGPf_5R			-	-			
	DGPf_6F			-	-	0	-	-
	DGPf_6R			-	-			
	DGPf_7F			-	-	0	-	-
	DGPf_7R			-	-			
	DGPf_8F			-	-	0	-	-
	DGPf_8R			-	-			
	DGPf_0F			1	0	612	24243	23632
	DGPf_0R			0	0			
	DGPf_1F			-	-	0	-	-
	DGPf_1R			-	-			
	DGPf_2F			1	0	1001	181025	180025
	DGPf_2R			0	0			
	DGPf_3F			-	-	0	-	-
	DGPf_3R	***P.* sp. RIT-PI-r**		-	-			
169	DGPf_4F	LIGE01000038.1	*P. koreensis*	-	-	0	-	-
	DGPf_4R	LIGE01000022.1		-	-			
	DGPf_5F			-	-	0	-	-
	DGPf_5R			-	-			
	DGPf_6F			-	-	0	-	-
	DGPf_6R			-	-			
	DGPf_7F			-	-	0	-	-
	DGPf_7R			-	-			
	DGPf_8F			-	-	0	-	-
	DGPf_8R			-	-			
	DGPf_0F			1	0	612	23963	24574
	DGPf_0R			1	0			
	DGPf_1F			-	-	0	-	-
	DGPf_1R			-	-			
	DGPf_2F			1	0	1001	6167	5167
	DGPf_2R			0	0			
	DGPf_3F			-	-	0	-	-
	DGPf_3R	***P. fluorescens* NZ011**		-	-			
170	DGPf_4F	AJXJ01000086.1	*P. koreensis*	-	-	0	-	-
	DGPf_4R	AJXJ01000004.1		-	-			
	DGPf_5F			-	-	0	-	-
	DGPf_5R			-	-			

	DGPf_6F			-	-	0	-	-
	DGPf_6R			-	-			
	DGPf_7F			-	-	0	-	-
	DGPf_7R			-	-			
	DGPf_8F			-	-	0	-	-
	DGPf_8R			-	-			
**171**	DGPf_0F	***P. fluorescens* R124**<br>ALYL01000069.1<br>ALYL01000049.1	*P. koreensis*	1	0	612	26882	26271
	DGPf_0R			0	0			
	DGPf_1F			-	-	0	-	-
	DGPf_1R			-	-			
	DGPf_2F			1	0	1001	3562	4562
	DGPf_2R			0	0			
	DGPf_3F			-	-	0	-	-
	DGPf_3R			-	-			
	DGPf_4F			-	-	0	-	-
	DGPf_4R			-	-			
	DGPf_5F			-	-	0	-	-
	DGPf_5R			-	-			
	DGPf_6F			-	-	0	-	-
	DGPf_6R			-	-			
	DGPf_7F			-	-	0	-	-
	DGPf_7R			-	-			
	DGPf_8F			-	-	0	-	-
	DGPf_8R			-	-			
**172**	DGPf_0F	***P.* sp. H1h**<br>AYMJ01000002.1<br>AYMJ01000018.1	*P. koreensis*	1	0	612	5856	6467
	DGPf_0R			0	0			
	DGPf_1F			-	-	0	-	-
	DGPf_1R			-	-			
	DGPf_2F			2	0	**1013**	93094	92082
	DGPf_2R			0	0			
	DGPf_3F			-	-	0	-	-
	DGPf_3R			-	-			
	DGPf_4F			-	-	0	-	-
	DGPf_4R			-	-			
	DGPf_5F			-	-	0	-	-
	DGPf_5R			-	-			
	DGPf_6F			-	-	0	-	-
	DGPf_6R			-	-			
	DGPf_7F			-	-	0	-	-
	DGPf_7R			-	-			
	DGPf_8F			-	-	0	-	-
	DGPf_8R			-	-			
**173**	DGPf_0F	***P.* sp. UW4**<br>CP003880.1	*P. jessenii*	1	0	612	5510253	5509642
	DGPf_0R			0	0			
	DGPf_1F			-	-	0	-	-
	DGPf_1R			-	-			
	DGPf_2F			1	0	**1013**	3601498	3602510
	DGPf_2R			0	0			
	DGPf_3F			0	0	681	2216136	2215456
	DGPf_3R			0	0			
	DGPf_4F			-	-	0	-	-
	DGPf_4R			-	-			
	DGPf_5F			-	-	0	-	-
	DGPf_5R			-	-			
	DGPf_6F			-	-	0	-	-
	DGPf_6R			-	-			
	DGPf_7F			-	-	0	-	-
	DGPf_7R			-	-			
	DGPf_8F			-	-	0	-	-
	DGPf_8R			-	-			
**174**	DGPf_0F	***P.* sp. GM48**<br>AKJM01000046.1<br>AKJM01000015.1<br>AKJM01000104.1	*P. jessenii*	1	0	612	81272	81883
	DGPf_0R			0	0			
	DGPf_1F			-	-	0	-	-
	DGPf_1R			-	-			
	DGPf_2F			1	0	1001	7440	8440
	DGPf_2R			0	0			
	DGPf_3F			0	0	681	19354	18674
	DGPf_3R			0	0			
	DGPf_4F			-	-	0	-	-
	DGPf_4R			-	-			
	DGPf_5F			-	-	0	-	-
	DGPf_5R			-	-			
	DGPf_6F			-	-	0	-	-
	DGPf_6R			-	-			
	DGPf_7F			-	-	0	-	-
	DGPf_7R			-	-			
	DGPf_8F			-	-	0	-	-
	DGPf_8R			-	-			
**175**	DGPf_0F	***P.* sp. GM55**<br>AKJJ01000045.1<br>AKJJ01000113.1<br>AKJJ01000036.1	*P. jessenii*	1	0	612	3588	2977
	DGPf_0R			0	0			
	DGPf_1F			-	-	0	-	-
	DGPf_1R			-	-			
	DGPf_2F			1	0	**1013**	9616	8604
	DGPf_2R			0	0			
	DGPf_3F			0	0	681	5311	4631
	DGPf_3R			0	0			
	DGPf_4F			-	-	0	-	-
	DGPf_4R			-	-			
	DGPf_5F			-	-	0	-	-
	DGPf_5R			-	-			

	DGPf_6F			-	-	0	-	-
	DGPf_6R			-	-			
	DGPf_7F			-	-	0	-	-
	DGPf_7R			-	-			
	DGPf_8F			-	-	0	-	-
	DGPf_8R			-	-			
	DGPf_0F			1	0	612	46725	47336
	DGPf_0R			0	0			
	DGPf_1F			-	-	0	-	-
	DGPf_1R			-	-			
	DGPf_2F			1	0	1013	11841	10829
	DGPf_2R			0	0			
	DGPf_3F			0	0	681	4190	4870
	DGPf_3R	***P.* sp. GM33**		0	0			
176	DGPf_4F	AKJO01000008.1	*P. jessenii*	-	-	0	-	-
	DGPf_4R	AKJO01000099.1		-	-			
	DGPf_5F	AKJO01000164.1		-	-	0	-	-
	DGPf_5R			-	-			
	DGPf_6F			-	-	0	-	-
	DGPf_6R			-	-			
	DGPf_7F			-	-	0	-	-
	DGPf_7R			-	-			
	DGPf_8F			-	-	0	-	-
	DGPf_8R			-	-			
	DGPf_0F			1	0	612	228591	227980
	DGPf_0R			0	0			
	DGPf_1F			-	-	0	-	-
	DGPf_1R			-	-			
	DGPf_2F			1	0	1013	19135	20147
	DGPf_2R			0	0			
	DGPf_3F			1	0	681	71390	72070
	DGPf_3R	***P. fluorescens* S613**		1	0			
177	DGPf_4F	LJXB01000093.1	*P. jessenii*	-	-	0	-	-
	DGPf_4R	LJXB01000048.1		-	-			
	DGPf_5F	LJXB01000090.1		-	-	0	-	-
	DGPf_5R			-	-			
	DGPf_6F			-	-	0	-	-
	DGPf_6R			-	-			
	DGPf_7F			-	-	0	-	-
	DGPf_7R			-	-			
	DGPf_8F			-	-	0	-	-
	DGPf_8R			-	-			
	DGPf_0F			1	0	612	3565	2954
	DGPf_0R			0	0			
	DGPf_1F			-	-	0	-	-
	DGPf_1R			-	-			
	DGPf_2F			1	0	1013	1278	266
	DGPf_2R			0	0			
	DGPf_3F			0	0	681	1053	373
	DGPf_3R	***P.* sp. GM49**		1	0			
178	DGPf_4F	AKJL01000029.1	*P. jessenii*	-	-	0	-	-
	DGPf_4R	AKJL01000309.1		-	-			
	DGPf_5F	AKJL01000111.1		-	-	0	-	-
	DGPf_5R			-	-			
	DGPf_6F			-	-	0	-	-
	DGPf_6R			-	-			
	DGPf_7F			-	-	0	-	-
	DGPf_7R			-	-			
	DGPf_8F			-	-	0	-	-
	DGPf_8R			-	-			
	DGPf_0F			1	0	612	2356	1745
	DGPf_0R			0	0			
	DGPf_1F			-	-	0	-	-
	DGPf_1R			-	-			
	DGPf_2F			1	0	1001	13494	14494
	DGPf_2R			0	0			
	DGPf_3F			1	0	681	7313	7993
	DGPf_3R	***P.* sp. GM74**		0	0			
179	DGPf_4F	AKJG01000161.1	*P. jessenii*	-	-	0	-	-
	DGPf_4R	AKJG01000086.1		-	-			
	DGPf_5F	AKJG01000049.1		-	-	0	-	-
	DGPf_5R			-	-			
	DGPf_6F			-	-	0	-	-
	DGPf_6R			-	-			
	DGPf_7F			-	-	0	-	-
	DGPf_7R			-	-			
	DGPf_8F			-	-	0	-	-
	DGPf_8R			-	-			
	DGPf_0F			1	0	612	80884	81495
	DGPf_0R			0	0			
	DGPf_1F			-	-	0	-	-
	DGPf_1R			-	-			
	DGPf_2F			1	0	1013	4749	5761
	DGPf_2R			0	0			
	DGPf_3F			0	0	681	163238	162558
	DGPf_3R	***P. fluorescens* C8**		1	0			
180	DGPf_4F	LACC01000022.1	*P. jessenii*	-	-	0	-	-
	DGPf_4R	LACC01000078.1		-	-			
	DGPf_5F	LACC01000005.1		-	-	0	-	-
	DGPf_5R			-	-			

	DGPf_6F			-	-	0	-	-
	DGPf_6R			-	-			
	DGPf_7F			-	-	0	-	-
	DGPf_7R			-	-			
	DGPf_8F			-	-	0	-	-
	DGPf_8R			-	-			
	DGPf_0F			1	0	612	230197	229586
	DGPf_0R			0	0			
	DGPf_1F			-	-	0	-	-
	DGPf_1R			-	-			
	DGPf_2F			1	0	**1013**	338942	339954
	DGPf_2R			0	0			
	DGPf_3F			0	0	681	15052	15732
	DGPf_3R	***P.* sp. Root71**		1	0			
**181**	DGPf_4F	LMHY01000030.1	*P. jessenii*	-	-	0	-	-
	DGPf_4R	LMHY01000028.1		-	-			
	DGPf_5F	LMHY01000007.1		-	-	0	-	-
	DGPf_5R			-	-			
	DGPf_6F			-	-	0	-	-
	DGPf_6R			-	-			
	DGPf_7F			-	-	0	-	-
	DGPf_7R			-	-			
	DGPf_8F			-	-	0	-	-
	DGPf_8R			-	-			
	DGPf_0F			1	0	612	230318	229707
	DGPf_0R			0	0			
	DGPf_1F			-	-	0	-	-
	DGPf_1R			-	-			
	DGPf_2F			1	0	**1013**	338631	339643
	DGPf_2R			0	0			
	DGPf_3F			0	0	681	14793	15473
	DGPf_3R	***P.* sp. Root68**		1	0			
**182**	DGPf_4F	LMHI01000025.1	*P. jessenii*	-	-	0	-	-
	DGPf_4R	LMHI01000023.1		-	-			
	DGPf_5F	LMHI01000006.1		-	-	0	-	-
	DGPf_5R			-	-			
	DGPf_6F			-	-	0	-	-
	DGPf_6R			-	-			
	DGPf_7F			-	-	0	-	-
	DGPf_7R			-	-			
	DGPf_8F			-	-	0	-	-
	DGPf_8R			-	-			
	DGPf_0F			-	-	0	-	-
	DGPf_0R			-	-			
	DGPf_1F			-	-	0	-	-
	DGPf_1R			-	-			
	DGPf_2F			1	0	1001	7349	8349
	DGPf_2R			0	0			
	DGPf_3F			0	0	681	308834	309514
	DGPf_3R	***P.* sp. Leaf48**		0	0			
**183**	DGPf_4F	LMLH01000021.1	*P. jessenii*	-	-	0	-	-
	DGPf_4R	LMLH01000032.1		-	-			
	DGPf_5F			-	-	0	-	-
	DGPf_5R			-	-			
	DGPf_6F			-	-	0	-	-
	DGPf_6R			-	-			
	DGPf_7F			-	-	0	-	-
	DGPf_7R			-	-			
	DGPf_8F			-	-	0	-	-
	DGPf_8R			-	-			
	DGPf_0F			2	0	612	34583	33972
	DGPf_0R			0	0			
	DGPf_1F			-	-	0	-	-
	DGPf_1R			-	-			
	DGPf_2F			1	0	1001	21319	22319
	DGPf_2R			0	0			
	DGPf_3F			1	0	681	4903	5583
	DGPf_3R	***P.* sp. GM78**		0	0			
**184**	DGPf_4F	AKJF01000088.1	*P. jessenii*	-	-	0	-	-
	DGPf_4R	AKJF01000095.1		-	-			
	DGPf_5F	AKJF01000164.1		-	-	0	-	-
	DGPf_5R			-	-			
	DGPf_6F			-	-	0	-	-
	DGPf_6R			-	-			
	DGPf_7F			-	-	0	-	-
	DGPf_7R			-	-			
	DGPf_8F			-	-	0	-	-
	DGPf_8R			-	-			
	DGPf_0F			1	0	612	4715200	4714589
	DGPf_0R			0	0			
	DGPf_1F			-	-	0	-	-
	DGPf_1R			-	-			
	DGPf_2F			1	0	**1013**	1234214	1233202
	DGPf_2R			0	0			
	DGPf_3F			1	0	681	2715543	2716223
	DGPf_3R	***P.* sp. 11/12A**		3	0			
**185**	DGPf_4F	JUGV01000001.1	*P. jessenii*	-	-	0	-	-
	DGPf_4R	JUGV01000001.1		-	-			
	DGPf_5F	JUGV01000001.1		-	-	0	-	-
	DGPf_5R			-	-			

	DGPf_6F			-	-	0	-	-
	DGPf_6R			-	-			
	DGPf_7F			-	-	0	-	-
	DGPf_7R			-	-			
	DGPf_8F			-	-	0	-	-
	DGPf_8R			-	-			
	DGPf_0F			2	0	612	24145	23534
	DGPf_0R			0	0			
	DGPf_1F			-	-	0	-	-
	DGPf_1R			-	-			
	DGPf_2F			1	0	1001	199333	200333
	DGPf_2R			0	0			
	DGPf_3F			0	0	681	34683	35363
	DGPf_3R	***P. fluorescens* C2**		1	0			
186	DGPf_4F	JSAK01000011.1	*P. jessenii*	-	-	0	-	-
	DGPf_4R	JSAK01000017.1		-	-			
	DGPf_5F	JSAK01000029.1		-	-	0	-	-
	DGPf_5R			-	-			
	DGPf_6F			-	-	0	-	-
	DGPf_6R			-	-			
	DGPf_7F			-	-	0	-	-
	DGPf_7R			-	-			
	DGPf_8F			-	-	0	-	-
	DGPf_8R			-	-			
	DGPf_0F			1	0	612	286358	285747
	DGPf_0R			0	0			
	DGPf_1F			-	-	0	-	-
	DGPf_1R			-	-			
	DGPf_2F			1	0	1001	14166	13166
	DGPf_2R			1	0			
	DGPf_3F			1	0	681	7484	6804
	DGPf_3R	***P. putida* CBB5**		0	0			
187	DGPf_4F	JTEN01000014.1	*P. jessenii*	-	-	0	-	-
	DGPf_4R	JTEN01000065.1		-	-			
	DGPf_5F	JTEN01000031.1		-	-	0	-	-
	DGPf_5R			-	-			
	DGPf_6F			-	-	0	-	-
	DGPf_6R			-	-			
	DGPf_7F			-	-	0	-	-
	DGPf_7R			-	-			
	DGPf_8F			-	-	0	-	-
	DGPf_8R			-	-			
	DGPf_0F			1	0	612	11282	10671
	DGPf_0R			0	0			
	DGPf_1F			-	-	0	-	-
	DGPf_1R			-	-			
	DGPf_2F			1	0	?	707 - 571<br>JOJW01000916.1	424 - 1296<br>JOJW01000179.1
	DGPf_2R			0	0			
	DGPf_3F	***P. putida* MC4-5222**		1	0	681	13261	12581
	DGPf_3R			1	0			
188	DGPf_4F	JOJW01001060.1	*P. jessenii*	-	-	0	-	-
	DGPf_4R	**JOJW01000916.1**		-	-			
	DGPf_5F	**JOJW01000179.1**		-	-	0	-	-
	DGPf_5R	JOJW01000617.1		-	-			
	DGPf_6F			-	-	0	-	-
	DGPf_6R			-	-			
	DGPf_7F			-	-	0	-	-
	DGPf_7R			-	-			
	DGPf_8F			-	-	0	-	-
	DGPf_8R			-	-			
	DGPf_0F			1	0	612	21666	22277
	DGPf_0R			0	0			
	DGPf_1F			-	-	0	-	-
	DGPf_1R			-	-			
	DGPf_2F			1	0	1001	10024	11024
	DGPf_2R			0	0			
	DGPf_3F			1	0	681	9030	9710
	DGPf_3R	***P.* sp. G5 (2012)**		1	0			
189	DGPf_4F	APIO01000016.1	*P. jessenii*	-	-	0	-	-
	DGPf_4R	APIO01000083.1		-	-			
	DGPf_5F	APIO01000022.1		-	-	0	-	-
	DGPf_5R			-	-			
	DGPf_6F			-	-	0	-	-
	DGPf_6R			-	-			
	DGPf_7F			-	-	0	-	-
	DGPf_7R			-	-			
	DGPf_8F			-	-	0	-	-
	DGPf_8R			-	-			
	DGPf_0F			2	0	612	307819	307208
	DGPf_0R			0	0			
	DGPf_1F			-	-	0	-	-
	DGPf_1R			-	-			
	DGPf_2F			1	0	1001	206635	205635
	DGPf_2R			1	0			
	DGPf_3F			3	0	681	364517	365197
	DGPf_3R	***P.* sp. Root562**		0	0			
190	DGPf_4F	LMGK01000023.1	*P. jessenii*	-	-	0	-	-
	DGPf_4R	LMGK01000006.1		-	-			
	DGPf_5F	LMGK01000025.1		-	-	0	-	-
	DGPf_5R			-	-			

	DGPf_6F			-	-	0	-	-
	DGPf_6R			-	-			
	DGPf_7F			-	-	0	-	-
	DGPf_7R			-	-			
	DGPf_8F			-	-	0	-	-
	DGPf_8R			-	-			
	DGPf_0F			1	0	612	308619	308008
	DGPf_0R			0	0			
	DGPf_1F			-	-	0	-	-
	DGPf_1R			-	-			
	DGPf_2F			-	-	0	-	-
	DGPf_2R			-	-			
	DGPf_3F			2	0	681	5603336	5604016
	DGPf_3R			0	0			
**191**	DGPf_4F	***P. fluorescens* NCIMB 11764**	*P. mandelii*	-	-	0	-	-
	DGPf_4R	CP010945.1		-	-			
	DGPf_5F			-	-	0	-	-
	DGPf_5R			-	-			
	DGPf_6F			-	-	0	-	-
	DGPf_6R			-	-			
	DGPf_7F			-	-	0	-	-
	DGPf_7R			-	-			
	DGPf_8F			-	-	0	-	-
	DGPf_8R			-	-			
	DGPf_0F			1	0	612	3746756	3747367
	DGPf_0R			0	0			
	DGPf_1F			-	-	0	-	-
	DGPf_1R			-	-			
	DGPf_2F			-	-	0	-	-
	DGPf_2R			-	-			
	DGPf_3F			0	0	681	5493125	5492445
	DGPf_3R			0	0			
**192**	DGPf_4F	***P.* sp. URMO17WK12:I11**	*P. mandelii*	-	-	0	-	-
	DGPf_4R	LN854573.1		-	-			
	DGPf_5F			-	-	0	-	-
	DGPf_5R			-	-			
	DGPf_6F			-	-	0	-	-
	DGPf_6R			-	-			
	DGPf_7F			-	-	0	-	-
	DGPf_7R			-	-			
	DGPf_8F			-	-	0	-	-
	DGPf_8R			-	-			
	DGPf_0F			1	0	612	4684738	4685349
	DGPf_0R			0	0			
	DGPf_1F			-	-	0	-	-
	DGPf_1R			-	-			
	DGPf_2F			-	-	0	-	-
	DGPf_2R			-	-			
	DGPf_3F			0	0	681	1255989	1256669
	DGPf_3R			1	0			
**193**	DGPf_4F	***P. mandelii* JR-1**	*P. mandelii*	-	-	0	-	-
	DGPf_4R	CP005960.1		-	-			
	DGPf_5F			-	-	0	-	-
	DGPf_5R			-	-			
	DGPf_6F			-	-	0	-	-
	DGPf_6R			-	-			
	DGPf_7F			-	-	0	-	-
	DGPf_7R			-	-			
	DGPf_8F			-	-	0	-	-
	DGPf_8R			-	-			
	DGPf_0F			1	0	612	29341	28730
	DGPf_0R			0	0			
	DGPf_1F			-	-	0	-	-
	DGPf_1R			-	-			
	DGPf_2F			-	-	0	-	-
	DGPf_2R			-	-			
	DGPf_3F			0	0	681	7624	8304
	DGPf_3R	***P.* sp. GM60**		0	0			
**194**	DGPf_4F	AKJI01000177.1	*P. mandelii*	-	-	0	-	-
	DGPf_4R	AKJI01000110.1		-	-			
	DGPf_5F			-	-	0	-	-
	DGPf_5R			-	-			
	DGPf_6F			-	-	0	-	-
	DGPf_6R			-	-			
	DGPf_7F			-	-	0	-	-
	DGPf_7R			-	-			
	DGPf_8F			-	-	0	-	-
	DGPf_8R			-	-			
	DGPf_0F			1	0	612	26467	25856
	DGPf_0R			0	0			
	DGPf_1F			-	-	0	-	-
	DGPf_1R			-	-			
	DGPf_2F			-	-	0	-	-
	DGPf_2R			-	-			
	DGPf_3F			0	0	681	12784	13464
	DGPf_3R	***P.* sp. GM67**		0	0			
**195**	DGPf_4F	AKJH01000100.1	*P. mandelii*	-	-	0	-	-
	DGPf_4R	AKJH01000049.1		-	-			
	DGPf_5F			-	-	0	-	-
	DGPf_5R			-	-			

	DGPf_6F			-	-	0	-	-
	DGPf_6R			-	-			
	DGPf_7F			-	-	0	-	-
	DGPf_7R			-	-			
	DGPf_8F			-	-	0	-	-
	DGPf_8R			-	-			
	DGPf_0F			1	0	612	87540	88151
	DGPf_0R			0	0			
	DGPf_1F			-	-	0	-	-
	DGPf_1R			-	-			
	DGPf_2F			-	-	0	-	-
	DGPf_2R			-	-			
	DGPf_3F			0	0	681	620309	619629
	DGPf_3R			0	0			
**196**	DGPf_4F	***P. umsongensis* UNC430CL58Col**<br>JHVT01000005.1<br>JHVT01000004.1	*P. mandelii*	-	-	0	-	-
	DGPf_4R			-	-			
	DGPf_5F			-	-	0	-	-
	DGPf_5R			-	-			
	DGPf_6F			-	-	0	-	-
	DGPf_6R			-	-			
	DGPf_7F			-	-	0	-	-
	DGPf_7R			-	-			
	DGPf_8F			-	-	0	-	-
	DGPf_8R			-	-			
	DGPf_0F			1	0	612	84235	84846
	DGPf_0R			0	0			
	DGPf_1F			-	-	0	-	-
	DGPf_1R			-	-			
	DGPf_2F			-	-	0	-	-
	DGPf_2R			-	-			
	DGPf_3F			0	0	681	1570527	1569847
	DGPf_3R			0	0			
**197**	DGPf_4F	***P.* sp. 45MFCol3.1**<br>ARMZ01000016.1<br>ARMZ01000022.1	*P. mandelii*	-	-	0	-	-
	DGPf_4R			-	-			
	DGPf_5F			-	-	0	-	-
	DGPf_5R			-	-			
	DGPf_6F			-	-	0	-	-
	DGPf_6R			-	-			
	DGPf_7F			-	-	0	-	-
	DGPf_7R			-	-			
	DGPf_8F			-	-	0	-	-
	DGPf_8R			-	-			
	DGPf_0F			1	0	612	201067	200456
	DGPf_0R			0	0			
	DGPf_1F			-	-	0	-	-
	DGPf_1R			-	-			
	DGPf_2F			-	-	0	-	-
	DGPf_2R			-	-			
	DGPf_3F			0	0	681	1041833	1041153
	DGPf_3R			0	0			
**198**	DGPf_4F	***P. mandelii* 36MFCvi1.1**<br>ARLP01000027.1<br>ARLP01000033.1	*P. mandelii*	-	-	0	-	-
	DGPf_4R			-	-			
	DGPf_5F			-	-	0	-	-
	DGPf_5R			-	-			
	DGPf_6F			-	-	0	-	-
	DGPf_6R			-	-			
	DGPf_7F			-	-	0	-	-
	DGPf_7R			-	-			
	DGPf_8F			-	-	0	-	-
	DGPf_8R			-	-			
	DGPf_0F			1	0	612	86033	86644
	DGPf_0R			0	0			
	DGPf_1F			-	-	0	-	-
	DGPf_1R			-	-			
	DGPf_2F			-	-	0	-	-
	DGPf_2R			-	-			
	DGPf_3F			0	0	681	27250	27930
	DGPf_3R			0	0			
**199**	DGPf_4F	***P.* sp. 35MFCvi1.1**<br>ARKL01000025.1<br>ARKL01000009.1	*P. mandelii*	-	-	0	-	-
	DGPf_4R			-	-			
	DGPf_5F			-	-	0	-	-
	DGPf_5R			-	-			
	DGPf_6F			-	-	0	-	-
	DGPf_6R			-	-			
	DGPf_7F			-	-	0	-	-
	DGPf_7R			-	-			
	DGPf_8F			-	-	0	-	-
	DGPf_8R			-	-			
	DGPf_0F			1	0	612	86033	86644
	DGPf_0R			0	0			
	DGPf_1F			-	-	0	-	-
	DGPf_1R			-	-			
	DGPf_2F			-	-	0	-	-
	DGPf_2R			-	-			
	DGPf_3F			0	0	681	59559	58879
	DGPf_3R			0	0			
**200**	DGPf_4F	***P. umsongensis* 20MFCvi1.1**<br>ARIW01000029.1<br>ARIW01000011.1	*P. mandelii*	-	-	0	-	-
	DGPf_4R			-	-			
	DGPf_5F			-	-	0	-	-
	DGPf_5R			-	-			

	DGPf_6F			-	-	0	-	-
	DGPf_6R			-	-			
	DGPf_7F			-	-	0	-	-
	DGPf_7R			-	-			
	DGPf_8F			-	-	0	-	-
	DGPf_8R			-	-			
	DGPf_0F			1	0	612	34785	34174
	DGPf_0R			0	0			
	DGPf_1F			-	-	0	-	-
	DGPf_1R			-	-			
	DGPf_2F			-	-	0	-	-
	DGPf_2R			-	-			
	DGPf_3F			0	0	681	7706	8386
	DGPf_3R	***P.* sp. GM18**		0	0			
**201**	DGPf_4F	AKJT01000005.1	*P. mandelii*	-	-	0	-	-
	DGPf_4R	AKJT01000102.1		-	-			
	DGPf_5F			-	-	0	-	-
	DGPf_5R			-	-			
	DGPf_6F			-	-	0	-	-
	DGPf_6R			-	-			
	DGPf_7F			-	-	0	-	-
	DGPf_7R			-	-			
	DGPf_8F			-	-	0	-	-
	DGPf_8R			-	-			
	DGPf_0F			1	0	612	232761	232150
	DGPf_0R			0	0			
	DGPf_1F			-	-	0	-	-
	DGPf_1R			-	-			
	DGPf_2F			-	-	0	-	-
	DGPf_2R			-	-			
	DGPf_3F			0	0	681	235358	236038
	DGPf_3R	***P.* sp. URMO17WK12:I12**		0	0			
**202**	DGPf_4F	AZVV01000011.1	*P. mandelii*	-	-	0	-	-
	DGPf_4R	AZVV01000013.1		-	-			
	DGPf_5F			-	-	0	-	-
	DGPf_5R			-	-			
	DGPf_6F			-	-	0	-	-
	DGPf_6R			-	-			
	DGPf_7F			-	-	0	-	-
	DGPf_7R			-	-			
	DGPf_8F			-	-	0	-	-
	DGPf_8R			-	-			
	DGPf_0F			1	0	612	90787	91398
	DGPf_0R			0	0			
	DGPf_1F			-	-	0	-	-
	DGPf_1R			-	-			
	DGPf_2F			-	-	0	-	-
	DGPf_2R			-	-			
	DGPf_3F			1	0	681	32694	32014
	DGPf_3R	***P. fluorescens* C3**		0	0			
**203**	DGPf_4F	LACD01000019.1	*P. mandelii*	-	-	0	-	-
	DGPf_4R	LACD01000013.1		-	-			
	DGPf_5F			-	-	0	-	-
	DGPf_5R			-	-			
	DGPf_6F			-	-	0	-	-
	DGPf_6R			-	-			
	DGPf_7F			-	-	0	-	-
	DGPf_7R			-	-			
	DGPf_8F			-	-	0	-	-
	DGPf_8R			-	-			
	DGPf_0F			1	0	612	37513	38124
	DGPf_0R			0	0			
	DGPf_1F			-	-	0	-	-
	DGPf_1R			-	-			
	DGPf_2F			-	-	0	-	-
	DGPf_2R			-	-			
	DGPf_3F			3	0	681	116321	115641
	DGPf_3R	***P. fluorescens* PA3G8**		0	0			
**204**	DGPf_4F	JBOO01000028.1	*P. mandelii*	-	-	0	-	-
	DGPf_4R	JBOO01000028.1		-	-			
	DGPf_5F			-	-	0	-	-
	DGPf_5R			-	-			
	DGPf_6F			-	-	0	-	-
	DGPf_6R			-	-			
	DGPf_7F			-	-	0	-	-
	DGPf_7R			-	-			
	DGPf_8F			-	-	0	-	-
	DGPf_8R			-	-			
	DGPf_0F			1	0	612	188990	188379
	DGPf_0R			0	0			
	DGPf_1F			-	-	0	-	-
	DGPf_1R			-	-			
	DGPf_2F			-	-	0	-	-
	DGPf_2R			-	-			
	DGPf_3F			3	0	681	106124	106804
	DGPf_3R			0	0			
**205**	DGPf_4F	***P. lini* DSM 16768**	*P. mandelii*	-	-	0	-	-
	DGPf_4R	JYLB01000010.1		-	-			
	DGPf_5F			-	-	0	-	-
	DGPf_5R			-	-			

	DGPf_6F			-	-	0	-	-
	DGPf_6R			-	-			
	DGPf_7F			-	-	0	-	-
	DGPf_7R			-	-			
	DGPf_8F			-	-	0	-	-
	DGPf_8R			-	-			
206	DGPf_0F	***P. lini*** **ZBG1**<br>LFQO01000013.1<br>LFQO01000014.1	*P. mandelii*	2	0	612	19006	19617
	DGPf_0R			0	0			
	DGPf_1F			-	-	0	-	-
	DGPf_1R			-	-			
	DGPf_2F			-	-	0	-	-
	DGPf_2R			-	-			
	DGPf_3F			4	0	681	15296	14616
	DGPf_3R			0	0			
	DGPf_4F			-	-	0	-	-
	DGPf_4R			-	-			
	DGPf_5F			-	-	0	-	-
	DGPf_5R			-	-			
	DGPf_6F			-	-	0	-	-
	DGPf_6R			-	-			
	DGPf_7F			-	-	0	-	-
	DGPf_7R			-	-			
	DGPf_8F			-	-	0	-	-
	DGPf_8R			-	-			
207	DGPf_0F	***P. fluorescens*** **H24**<br>LACH01000050.1<br>LACH01000049.1	*P. mandelii*	1	0	612	63356	62745
	DGPf_0R			0	0			
	DGPf_1F			-	-	0	-	-
	DGPf_1R			-	-			
	DGPf_2F			-	-	0	-	-
	DGPf_2R			-	-			
	DGPf_3F			0	0	681	1874	2554
	DGPf_3R			0	0			
	DGPf_4F			-	-	0	-	-
	DGPf_4R			-	-			
	DGPf_5F			-	-	0	-	-
	DGPf_5R			-	-			
	DGPf_6F			-	-	0	-	-
	DGPf_6R			-	-			
	DGPf_7F			-	-	0	-	-
	DGPf_7R			-	-			
	DGPf_8F			-	-	0	-	-
	DGPf_8R			-	-			
208	DGPf_0F	***P.*** **sp. RIT-PI-q**<br>LHPC01000007.1<br>LHPC01000048.1<br>**LHPC01000054.1**	*P. mandelii*	1	0	612	241079	240468
	DGPf_0R			0	0			
	DGPf_1F			-	-	0	-	-
	DGPf_1R			-	-			
	DGPf_2F			-	-	0	-	-
	DGPf_2R			-	-			
	DGPf_3F			1	0	681	9524	8844
	DGPf_3R			0	0			
	DGPf_4F			-	-	0	-	-
	DGPf_4R			-	-			
	DGPf_5F			-	-	0	-	-
	DGPf_5R			-	-			
	DGPf_6F			-	-	0	-	-
	DGPf_6R			-	-			
	DGPf_7F			**4**	**0**	**756**	**153323**	**154078**
	DGPf_7R			**5**	**0**			
	DGPf_8F			-	-	0	-	-
	DGPf_8R			-	-			
209	DGPf_0F	***P. mandelii*** **PD30**<br>AZQQ01000060.1<br>AZQQ01000074.1	*P. mandelii*	1	0	612	4900	5511
	DGPf_0R			0	0			
	DGPf_1F			-	-	0	-	-
	DGPf_1R			-	-			
	DGPf_2F			-	-	0	-	-
	DGPf_2R			-	-			
	DGPf_3F			0	0	681	97922	98602
	DGPf_3R			0	0			
	DGPf_4F			-	-	0	-	-
	DGPf_4R			-	-			
	DGPf_5F			-	-	0	-	-
	DGPf_5R			-	-			
	DGPf_6F			-	-	0	-	-
	DGPf_6R			-	-			
	DGPf_7F			-	-	0	-	-
	DGPf_7R			-	-			
	DGPf_8F			-	-	0	-	-
	DGPf_8R			-	-			
210	DGPf_0F	***P.*** **sp. GM41 (2012)**<br>AKJN02000007.1<br>AKJN02000006.1	*P. mandelii*	1	0	612	879294	879905
	DGPf_0R			0	0			
	DGPf_1F			-	-	0	-	-
	DGPf_1R			-	-			
	DGPf_2F			-	-	0	-	-
	DGPf_2R			-	-			
	DGPf_3F			0	0	681	1381179	1381859
	DGPf_3R			0	0			
	DGPf_4F			-	-	0	-	-
	DGPf_4R			-	-			
	DGPf_5F			-	-	0	-	-
	DGPf_5R			-	-			

	DGPf_6F			-	-	0	-	-
	DGPf_6R			-	-			
	DGPf_7F			-	-	0	-	-
	DGPf_7R			-	-			
	DGPf_8F			-	-	0	-	-
	DGPf_8R			-	-			
211	DGPf_0F	***P.* sp. Root329**<br>LMCV01000029.1<br>LMCV01000045.1	*P. mandelii*	1	0	612	29830	29219
	DGPf_0R			0	0			
	DGPf_1F			-	-	0	-	-
	DGPf_1R			-	-			
	DGPf_2F			-	-	0	-	-
	DGPf_2R			-	-			
	DGPf_3F			0	0	681	122909	123589
	DGPf_3R			0	0			
	DGPf_4F			-	-	0	-	-
	DGPf_4R			-	-			
	DGPf_5F			-	-	0	-	-
	DGPf_5R			-	-			
	DGPf_6F			-	-	0	-	-
	DGPf_6R			-	-			
	DGPf_7F			-	-	0	-	-
	DGPf_7R			-	-			
	DGPf_8F			-	-	0	-	-
	DGPf_8R			-	-			
212	DGPf_0F	***P.* sp. GM21**<br>AKJS01000110.1<br>AKJS01000051.1	*P. mandelii*	2	0	612	7203	7814
	DGPf_0R			0	0			
	DGPf_1F			-	-	0	-	-
	DGPf_1R			-	-			
	DGPf_2F			-	-	0	-	-
	DGPf_2R			-	-			
	DGPf_3F			0	0	681	4999	4319
	DGPf_3R			1	0			
	DGPf_4F			-	-	0	-	-
	DGPf_4R			-	-			
	DGPf_5F			-	-	0	-	-
	DGPf_5R			-	-			
	DGPf_6F			-	-	0	-	-
	DGPf_6R			-	-			
	DGPf_7F			-	-	0	-	-
	DGPf_7R			-	-			
	DGPf_8F			-	-	0	-	-
	DGPf_8R			-	-			
213	DGPf_0F	***P.* sp. GM102**<br>AKJB01000113.1<br>AKJB01000112.1	*P. mandelii*	1	0	612	2346	1735
	DGPf_0R			0	0			
	DGPf_1F			-	-	0	-	-
	DGPf_1R			-	-			
	DGPf_2F			-	-	0	-	-
	DGPf_2R			-	-			
	DGPf_3F			1	0	681	6425	7105
	DGPf_3R			0	0			
	DGPf_4F			-	-	0	-	-
	DGPf_4R			-	-			
	DGPf_5F			-	-	0	-	-
	DGPf_5R			-	-			
	DGPf_6F			-	-	0	-	-
	DGPf_6R			-	-			
	DGPf_7F			-	-	0	-	-
	DGPf_7R			-	-			
	DGPf_8F			-	-	0	-	-
	DGPf_8R			-	-			
214	DGPf_0F	***P. fluorescens* C1**<br>LACE01000007.1<br>LACE01000011.1	*P. mandelii*	1	0	612	231724	231113
	DGPf_0R			0	0			
	DGPf_1F			-	-	0	-	-
	DGPf_1R			-	-			
	DGPf_2F			-	-	0	-	-
	DGPf_2R			-	-			
	DGPf_3F			0	0	681	188301	188981
	DGPf_3R			1	0			
	DGPf_4F			-	-	0	-	-
	DGPf_4R			-	-			
	DGPf_5F			-	-	0	-	-
	DGPf_5R			-	-			
	DGPf_6F			-	-	0	-	-
	DGPf_6R			-	-			
	DGPf_7F			-	-	0	-	-
	DGPf_7R			-	-			
	DGPf_8F			-	-	0	-	-
	DGPf_8R			-	-			
215	DGPf_0F	***P.* sp. QTF5**<br>AZRW01000055.1<br>AZRW01000054.1	*P. mandelii*	1	0	612	254981	255592
	DGPf_0R			0	0			
	DGPf_1F			-	-	0	-	-
	DGPf_1R			-	-			
	DGPf_2F			-	-	0	-	-
	DGPf_2R			-	-			
	DGPf_3F			4	0	681	9679	8999
	DGPf_3R			0	0			
	DGPf_4F			-	-	0	-	-
	DGPf_4R			-	-			
	DGPf_5F			-	-	0	-	-
	DGPf_5R			-	-			

	DGPf_6F			-	-	0	-	-
	DGPf_6R			-	-			
	DGPf_7F			-	-	0	-	-
	DGPf_7R			-	-			
	DGPf_8F			-	-	0	-	-
	DGPf_8R			-	-			
	DGPf_0F			1	0	612	28156	27545
	DGPf_0R			0	0			
	DGPf_1F			-	-	0	-	-
	DGPf_1R			-	-			
	DGPf_2F			-	-	0	-	-
	DGPf_2R			-	-			
	DGPf_3F			1	0	681	45281	45961
	DGPf_3R	***P.* sp. GM79**		0	0			
**216**	DGPf_4F	AKJE01000081.1	*P. mandelii*	-	-	0	-	-
	DGPf_4R	AKJE01000036.1		-	-			
	DGPf_5F			-	-	0	-	-
	DGPf_5R			-	-			
	DGPf_6F			-	-	0	-	-
	DGPf_6R			-	-			
	DGPf_7F			-	-	0	-	-
	DGPf_7R			-	-			
	DGPf_8F			-	-	0	-	-
	DGPf_8R			-	-			
	DGPf_0F			1	0	612	24135	23524
	DGPf_0R			0	0			
	DGPf_1F			-	-	0	-	-
	DGPf_1R			-	-			
	DGPf_2F			-	-	0	-	-
	DGPf_2R			-	-			
	DGPf_3F			3	0	681	64317	64997
	DGPf_3R	***P.* sp. GM50**		0	0			
**217**	DGPf_4F	AKJK01000044.1	*P. mandelii*	-	-	0	-	-
	DGPf_4R	AKJK01000087.1		-	-			
	DGPf_5F			-	-	0	-	-
	DGPf_5R			-	-			
	DGPf_6F			-	-	0	-	-
	DGPf_6R			-	-			
	DGPf_7F			-	-	0	-	-
	DGPf_7R			-	-			
	DGPf_8F			-	-	0	-	-
	DGPf_8R			-	-			
	DGPf_0F			1	0	612	53529	52918
	DGPf_0R			1	0			
	DGPf_1F			-	-	0	-	-
	DGPf_1R			-	-			
	DGPf_2F			-	-	0	-	-
	DGPf_2R			-	-			
	DGPf_3F			3	0	681	37602	36922
	DGPf_3R	***P. syringae* Riq4**		0	0			
**218**	DGPf_4F	LFQK01000058.1	*P. mandelii*	-	-	0	-	-
	DGPf_4R	LFQK01000045.1		-	-			
	DGPf_5F			-	-	0	-	-
	DGPf_5R			-	-			
	DGPf_6F			-	-	0	-	-
	DGPf_6R			-	-			
	DGPf_7F			-	-	0	-	-
	DGPf_7R			-	-			
	DGPf_8F			-	-	0	-	-
	DGPf_8R			-	-			

False possitives

	DGPf_0F			1	0	612	24135	23524
	DGPf_0R			0	0			
	DGPf_1F			-	-	0	-	-
	DGPf_1R			-	-			
	DGPf_2F			2	0	1001	17234	18234
	DGPf_2R			2	0			
	DGPf_3F			3	0	681	64317	64997
	DGPf_3R			0	0			
1	DGPf_4F	***P.* sp. ML96**	*P. jessenii*	-	-	0	-	-
	DGPf_4R	AKJK0100		-	-			
	DGPf_5F			-	-	0	-	-
	DGPf_5R			-	-			
	DGPf_6F			-	-	0	-	-
	DGPf_6R			-	-			
	DGPf_7F			-	-	0	-	-
	DGPf_7R			-	-			
	DGPf_8F			-	-	0	-	-
	DGPf_8R			-	-			
	DGPf_0F			-	-	0	-	-
	DGPf_0R			-	-			

	DGPf_1F			-	-	0	-	-
	DGPf_1R			-	-			
	DGPf_2F			3	0	1001	1515	515
	DGPf_2R			1	0			
	DGPf_3F			6	0	681	10406	11086
	DGPf_3R			1	0			
	DGPf_4F	***P. pseudoalcaligenes* KF707**		-	-	0	-	-
2	DGPf_4R	AJMR01000037.1	*P. jessenii*	-	-			
	DGPf_5F	AJMR01000096.1		-	-	0	-	-
	DGPf_5R			-	-			
	DGPf_6F			-	-	0	-	-
	DGPf_6R			-	-			
	DGPf_7F			-	-	0	-	-
	DGPf_7R			-	-			
	DGPf_8F			-	-	0	-	-
	DGPf_8R			-	-			
	DGPf_0F			-	-	0	-	-
	DGPf_0R			-	-			
	DGPf_1F			-	-	0	-	-
	DGPf_1R			-	-			
	DGPf_2F			2	0	1001	48551	49551
	DGPf_2R			1	0			
	DGPf_3F			4	0	681	51035	50355
	DGPf_3R			1	0			
	DGPf_4F	***P. alcaligenes* NBRC 14159**		-	-	0	-	-
3	DGPf_4R	BATI01000040.1	*P. jessenii*	-	-			
	DGPf_5F	BATI01000028.1		-	-	0	-	-
	DGPf_5R			-	-			
	DGPf_6F			-	-	0	-	-
	DGPf_6R			-	-			
	DGPf_7F			-	-	0	-	-
	DGPf_7R			-	-			
	DGPf_8F			-	-	0	-	-
	DGPf_8R			-	-			
	DGPf_0F			-	-	0	-	-
	DGPf_0R			-	-			
	DGPf_1F			-	-	0	-	-
	DGPf_1R			-	-			
	DGPf_2F			3	0	1001	54657	55657
	DGPf_2R			1	0			
	DGPf_3F			2	0	681	213322	214002
	DGPf_3R			3	0			
	DGPf_4F	***P.* sp. LFM046**		-	-	0	-	-
4	DGPf_4R	JYKO01000020.1	*P. jessenii*	-	-			
	DGPf_5F	JYKO01000031.1		-	-	0	-	-
	DGPf_5R			-	-			
	DGPf_6F			-	-	0	-	-
	DGPf_6R			-	-			
	DGPf_7F			-	-	0	-	-
	DGPf_7R			-	-			
	DGPf_8F			-	-	0	-	-
	DGPf_8R			-	-			
	DGPf_0F			-	-	0	-	-
	DGPf_0R			-	-			
	DGPf_1F			-	-	0	-	-
	DGPf_1R			-	-			
	DGPf_2F			5	0	1001	20728	19728
	DGPf_2R			1	0			
	DGPf_3F			3	0	681	934	1614
	DGPf_3R			1	0			
	DGPf_4F	***P. thermotolerans* J53**		-	-	0	-	-
5	DGPf_4R	AZUT01000007.1	*P. jessenii*	-	-			
	DGPf_5F	AZUT01000051.1		-	-	0	-	-
	DGPf_5R			-	-			
	DGPf_6F			-	-	0	-	-
	DGPf_6R			-	-			
	DGPf_7F			-	-	0	-	-
	DGPf_7R			-	-			
	DGPf_8F			-	-	0	-	-
	DGPf_8R			-	-			
	DGPf_0F			4	0	612	30690	31301
	DGPf_0R			4	0			
	DGPf_1F			-	-	0	-	-
	DGPf_1R			-	-			
	DGPf_2F			1	0	1001	15784	16784
	DGPf_2R			2	0			
	DGPf_3F			-	-	0	-	-
	DGPf_3R			-	-			
	DGPf_4F	***P. alcaligenes* OT 69**		-	-	0	-	-
6	DGPf_4R	ATCP01000068.1	*P. koreensis*	-	-			
	DGPf_5F	ATCP01000013.1		-	-	0	-	-
	DGPf_5R			-	-			
	DGPf_6F			-	-	0	-	-
	DGPf_6R			-	-			
	DGPf_7F			-	-	0	-	-
	DGPf_7R			-	-			
	DGPf_8F			-	-	0	-	-
	DGPf_8R			-	-			
	DGPf_0F			4	0	612	15350	14739
	DGPf_0R			4	0			

7	DGPf_1F<br>DGPf_1R	***P. alcaligenes* MRY13-0052**<br>BATO01000091.1<br>BATO01000093.1	*P. koreensis*	-<br>-	-<br>-	0	-	-
	DGPf_2F<br>DGPf_2R			1<br>2	0<br>0	1001	11025	12025
	DGPf_3F<br>DGPf_3R			-<br>-	-<br>-	0	-	-
	DGPf_4F<br>DGPf_4R			-<br>-	-<br>-	0	-	-
	DGPf_5F<br>DGPf_5R			-<br>-	-<br>-	0	-	-
	DGPf_6F<br>DGPf_6R			-<br>-	-<br>-	0	-	-
	DGPf_7F<br>DGPf_7R			-<br>-	-<br>-	0	-	-
	DGPf_8F<br>DGPf_8R			-<br>-	-<br>-	0	-	-
